# Toward better prevention of physician burnout: insights from individual participant data using the MD-specific Occupational Stressor Index and organizational interventions

**DOI:** 10.3389/fpubh.2025.1514706

**Published:** 2025-03-19

**Authors:** Karen Belkić

**Affiliations:** ^1^Department of Oncology/Pathology, Karolinska Institute, Stockholm, Sweden; ^2^Department of Medical Radiation Physics and Nuclear Medicine, Karolinska University Hospital, Stockholm, Sweden; ^3^School of Community and Global Health, Claremont Graduate University, Claremont, CA, United States; ^4^Institute for Health Promotion and Disease Prevention Research, University of Southern California School of Medicine, Los Angeles, CA, United States

**Keywords:** work conditions, physicians, occupational health, burnout, participatory action research

## Abstract

**Background:**

Physician burnout has become a public-health crisis. The need is dire for robust organizational solutions, focusing on reduction of specific stressors. The physician-specific Occupational Stressor Index (OSI) based on cognitive ergonomics can help. Individual-participant data (IPD) from different studies addressing physician burnout are lacking.

**Aims:**

To perform IPD analysis regarding job stressors and their relation to physician burnout and to utilize the IPD results to inform a systematic review of the stressors that show an association with physician burnout, focusing on intervention studies.

**Methods:**

PRISMA guidelines are followed for the IPD analysis and systematic review of intervention studies on the implicated stressors, taking the COVID-19 pandemic into consideration. The IPD analysis is performed on studies using the physician-specific OSI vis-à-vis burnout assessed by the Copenhagen Burnout Inventory (CBI). Odds ratios (OR) ± 95% confidence-intervals (CI) are reported, adjusting for age, gender and caring for patients with suspected COVID-19 infection.

**Results:**

Three studies fulfilled the inclusion criteria, providing complete IPD data for 95 physicians. Thirty-two (33.7%) physicians had total OSI scores >88, for which intervention is urgently needed. Unit-change in the total stressor burden assessed via OSI yielded OR = 1.11 (95%CI: 1.03–1.18) (*p* = 0.003) for personal burnout, OR = 1.17 (95%CI: 1.08–1.26) (*p* = 0.0001) for work-related burnout and OR = 1.07 (95%CI: 1.01–1.15) (*p* = 0.03) for patient-related burnout. Caring for patients with suspected COVID-19 infection showed significant multivariable results (*p* = 0.04) only for personal burnout. Twenty distinct work stressors revealed multivariable associations with CBI. Systematic examination via PUBMED, CINAHL and OVID Medline yielded 33 publications mitigating those stressors among physicians. Adequate staffing was pivotal. Clerical staff off-loaded administrative burden. Information-technology staff helped diminish interruptions, enhancing workflow. Cross-coverage reduced time constraints, ensured separate periods for non-clinical tasks, and ≥1 work-free day/week. Several interventions impacted physician burnout, as did recognition of physicians' efforts/achievements. Other OSI-identified stressors were insufficiently examined in intervention studies: e.g. vacation; appropriately-timed, cross-covered restbreaks; and counter-measures for emotionally-disturbing aspects of MD's work, particularly during the pandemic.

**Conclusions:**

Further participatory-action research is needed in well-controlled intervention trials to alleviate physician burnout.

## 1 Introduction

High rates of burnout have been reported among physicians for many years (1–3). In the most recent period, physician burnout has aroused even more attention, with the added burden of the COVID-19 pandemic (4–7). According to major health care organizations, physician burnout represents a public health crisis. Implicit in this clarion call is a call for action, especially given that burnout is reversible and preventable (8).

Having been rigorously selected to be mentally healthy and after finishing long, arduous preparation prior to starting actual professional life, a “super-healthy worker effect” is anticipated in physicians. Namely, compared to other populations, physicians would be expected to have far lower prevalence of untoward psychological outcomes (9). The fact that the situation is just the contrary, i.e., that burnout and even more serious mental health disorders, including risk of suicide, are actually more widespread among physicians (2, 10), strongly implicates exposure to deleterious work conditions. Consequently, identification of the contributory job stressors and, whenever possible, their alleviation, become the priority.

Of late, cognitive ergonomics and how it could inform interventions have received renewed attention directed toward physicians (11–13). Yet, there exists a comprehensive work stressor model based upon cognitive ergonomics and brain research (14), which has been effectively applied among several occupational groups, including physicians. That theory-based model, the Occupational Stressor Index (OSI), has been fully-operationalized specifically for physicians (15). The latter was achieved through “participatory action research” (16), reflecting hands-on experience and was presented to colleagues as “for physicians, by physicians” (15).

The overall stressor load of a given work environment is reflected in the total OSI score. When that exceeds the clinical cutpoint of 88, intervention is urgently required (17, 18). The OSI model includes major dimensions of the generic work stressor models, e.g., “high demands” (14, 19), operationalizing these concretely regarding time and allocation of mental resources, as germane to the specific occupation. Informed by cognitive ergonomics, the OSI model also considers other dimensions. In particular, the possibility of encountering harm contributes heavily to the stressor burden (14), with the nervous system selectively attending to threatening stimuli (20, 21). The term “threat avoidant vigilance” denotes having to follow such stimuli, responding quickly, with errors or delays having serious, potentially fatal consequences (14, 20, 22, 23). Without consideration of threat avoidant vigilance, the stressor burden of physicians, as well as that of nurses, airline pilots, professional drivers, police and firefighters, among others, is markedly underestimated (15, 18, 23). Importantly, the OSI includes stressor aspects such as threat avoidant vigilance, missing from the sociological models (14).

The OSI affords an in-depth qualitative and quantitative description of the stressor burden (15, 18, 23), similar to theory-guided, on-site evaluation of the mental structure of job tasks (24, 25). Since the OSI is questionnaire-based, it does not require on-the-job analysis, but its diagnostic accuracy can be thereby further enhanced. This is especially helpful for design and implementation of interventions.

The OSI questionnaires are constructed to be relevant and succinct, with the queries presented in an order which is logical for the participants from a given occupation. The responses are then coded according to the OSI model, such that all the specific OSI's are numerically and theoretically compatible. The OSI for physicians questionnaire and the OSI score sheet are available at Supplemental Digital Content, http://links.lww.com/SMJ/A230 and http://links.lww.com/SMJ/A231. Further details can be found in Belkić and Savić (18).

Burnout has been assessed with a number of validated instruments (26–29), as well as by some explicit, single-item queries (30–33). The Maslach Burnout Inventory (MBI) is based upon the original definition of burnout as a syndrome of “emotional exhaustion, depersonalization, and reduced personal accomplishment” (26). The Copenhagen Burnout Inventory (CBI) has been widely used in the international setting, being directly available without charge. According to its Authors, the CBI appropriately focuses on the key attribute of fatigue and exhaustion applied to specific domains of one's life. The CBI avoids queries concerning depersonalization that may elicit negative reactions in many cultures (29). There are three components in the CBI: (A) personal burnout, as physical and emotional exhaustion not explicitly connected to work; (B) work-related burnout, assessing how much the former is actually associated with one's job and (C) querying about the linkage between physical and emotional exhaustion while working with patients or other “clients” (29). The CBI is especially appropriate for physicians, and has been used in many health care settings internationally, prior to as well as during the height of the COVID-19 pandemic (3, 4, 6, 9, 29, 34–40).

There have been a number of published reviews addressing physician burnout, suggesting that individual-focused and organizational measures could help mitigate or prevent its occurrence (13, 41–46). However, the evidence is considered overall of low quality. In particular, organizational interventions that “focus on reduction of specific stressors” are sparse (45) (p. 2). The physician-specific OSI, with its basis in cognitive ergonomics, and objective, comprehensive, quantifiable assessment of work-conditions, could contribute to this goal. It also warrants note that, to the best of our knowledge after extensive searching, the published reviews on physician burnout have relied upon aggregate data from various publications. In other words, the original data on each participating physician have not been jointly analyzed across studies. Assessing individual-level data from all studies that address a specific clinical-research question is “considered a gold-standard approach to evidence synthesis” (47) (p. 1657). This is one of the aims of the present study, namely to perform individual participant data (IPD) analysis regarding job stressors and their relation to physician burnout. The physician-specific OSI will be the method of analysis for work conditions, with the CBI as the assessment measure of outcomes. The second aim is to utilize the results of the IPD to inform a systematic review of each of the stressors that show an association with physician burnout. The main focus of the latter will be to identify and assess published intervention studies targeting at the mitigation or elimination of the implicated work stressor among physicians. The impact of the COVID-19 pandemic will be taken into consideration throughout.

## 2 Methods

### 2.1 Eligibility criteria for the IPD

For inclusion in the IPD, study participants should have been full-time employed physicians, with the physician-specific OSI used to evaluate their work conditions and burnout assessed via the CBI, as the outcome, and basic demographic data (age and gender) available. There was no restriction on study design nor year(s) when conducted.

### 2.2 Identification of studies

**Figure 1:**
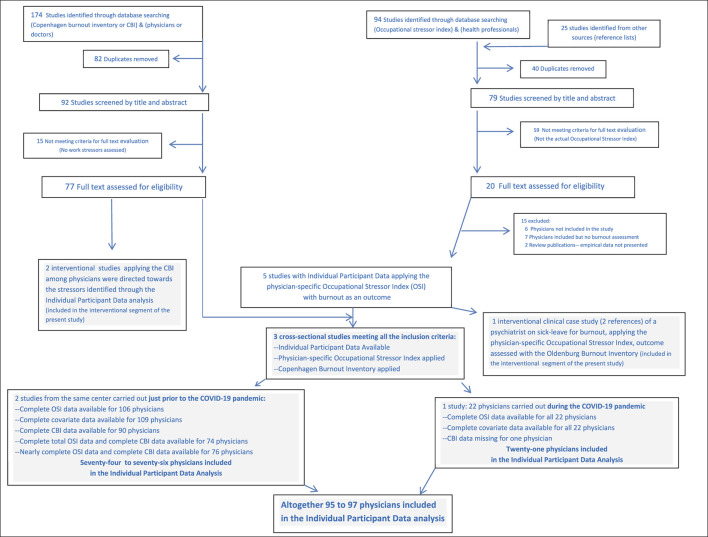
Flow chart for selection of studies addressing physician burnout, included in the individual participant data analysis as per PRISMA guidelines.

The electronic search engines in this and all the other searches for this article were: PUBMED, CINAHL, and Ovid Medline, conducted through June 2024. Studies identified from other sources such as reference lists were also considered. As summarized in Figure 1 for the IPD studies, two search branches were implemented, with details in Supplementary material 1. The first search combined (physicians or doctors) with (Copenhagen Burnout Inventory or CBI). The second search combined (Occupational Stressor Index) with (health professionals). After merging the two branches, five studies were found applying the physician-specific OSI with burnout as an outcome. Two of these described a clinical interventional for a psychiatrist on sick-leave for burnout (18, 48). However, the Oldenburg Burnout Inventory had been used therein, such that these did not fully meet the requirements for the IPD. They were included in part II of the present paper, focusing on interventions. Three studies from two centers fulfilled all the inclusion criteria, yielding IPD data for 95–97 physicians (9, 34, 39). These fully anonymized data were made available for the present analysis. As per Goyal et al. (9), Belkić and Rustagi (34), and Nedić and Belkić (39), all the research, as well as the treatment of the current anonymized data are in full accordance with the World Medical Association's Declaration of Helsinki.

### 2.3 Analysis of the individual participant data

The available data for the IPD were all cross-sectional, i.e., baseline only. Two of the studies had been carried out in English in which all the participants were entirely fluent, on the level of a first-language (9, 34). All materials for the third study (39) were in Serbian, the primary language of all the participating physicians. Translation-back-translation was used to verify the equivalence of the Serbian language versions of all the instruments with respect to the originals in English.

Studies (9, 34) from the All India Institute of Medical Sciences in Jodhpur were carried out among physicians in 2018–2019, i.e., just prior to the outbreak of the COVID-19 pandemic. The participation rate was 43%. Data for Nedić and Belkić (39) were collected from November 2021 through April 2022 among primary care physicians and nurses working at the COVID-19 Outpatient Respiratory Center in Novi Sad, Serbia, with 100% participation. The present IPD analysis from Nedić and Belkić (39) includes only the physicians.

The data from the 3 studies were placed into a single data set. Therein, each study was labeled, on the basis of which a variable was introduced as to whether the data were collected during or prior to the onset of the COVID-19 pandemic. The OSI data were complete for 106 physicians from Jodhpur and for all 22 participating physicians from Novi Sad. Complete CBI data were available for 90 physicians from Jodhpur and for 21 of the 22 physicians from Novi Sad. Covariates were age, gender and work-years as a physician, complete for 109 Jodhpur physicians and all 22 of those from Novi Sad. Altogether there were complete data for 95 physicians (74 from Jodhpur and 21 from Novi Sad) and nearly complete data for 97 physicians (76 from Jodhpur and 21 from Novi Sad).

Firstly, extensive univariate analysis was performed. Age and working years as a physician were strongly correlated (Pearson *r* = 0.85, *p* = 0.000). To avoid multicollinearity, age was selected as the covariate for inclusion in all the multivariable analysis. Gender and work during vs. prior to the COVID-19 pandemic were the other two covariables included in all the multivariable analyses.

The outcome measures [personal (A), work-related (B) and patient-related burnout (C)] were dichotomized at their respective median cutpoints for multiple logistic regression, which was used to compute odds ratios (OR) and 95% confidence intervals (CI). Multiple logistic regression (M Log R) was applied to assess the effect of unit change in the total OSI on burnout (A), (B), and (C). In addition, M Log R was carried out with total OSI scores dichotomized at the clinically-determined cutpoint of 88, above which urgent intervention has been deemed to be needed (17, 18). Next, the relation between work stressors as evaluated using the OSI questionnaire and each of the burnout components was assessed via M Log R. Results with *p* < 0.05 for the OSI stressor are displayed, as are trends, generally with 0.05 ≤ *p* ≤ 0.08.

The internal consistency, examined by the Standardized Cronbach alpha was 0.84 for the total OSI, 0.90 for burnout (A), 0.87 for burnout (B), and 0.92 for burnout (C). We tested for the potential random effect of work during vs. prior to the COVID-19 pandemic via Variance Components Analysis, with respect to all three CBI outcome variables. The *p* values were identical or very close to those obtained in M Log R analysis.

Only complete data were used in the analyses, with no imputation whatsoever. According to the outlined search strategies, all potentially eligible studies were included for the IPD analysis. We consider this single-stage IPD analysis to be complete for the investigated questions. Statistica software (14.0.0.15, 2021 TIBCO version) was used throughout for the IPD analysis.

### 2.4 Systematic literature review

A systematic literature review was undertaken for each of the OSI stressors for which there was a multivariable association with one or more of the burnout indices. A very brief presentation of observational investigations is given in the main text, highlighting a few of the most salient findings, with the search strategies and further details in Supplementary material 2. A major focus in the Results Section will be on intervention studies impacting the job stressor and carried out among physicians, with particular attention to burnout when assessed as an outcome.

## 3 Results

### 3.1 Univariate findings and multiple logistic regression for total OSI among the physicians with IPD

Table 1 provides a summary of the major univariate findings for the physicians included in the IPD analysis. These encompass key demographic data, the total OSI scores, the totals for the OSI levels and OSI aspects, and CBI personal, work-related and patient-related burnout scores. There were quite similar percentages of practice areas: General practice/community medicine, Surgery/anesthesia/emergency medicine and Internal medicine/pediatrics/psychiatry/dermatology/combined specialties. Thirty-two (33.7%) of the 95 physicians included in the IPD analysis for whom these data were available had a total OSI score above the cutoff point of 88, for which intervention is urgently needed.

**Table 1:** Major univariate findings for the physicians included in the individual participant data analysis.

	***N***	**Mean**	**Sd**	**Median**	**IQR**
Age	97	32.1	7.6	29	11.0
Working years as a physicians (0 = <1 y, 1 = 1–5 y, 2 = 5–10 y, 3 > 10 y)	97	1.47	1.0	1.0	1.0
Total Occupational Stressor Index (OSI)	95	85.3	8.3	84.9	10.6
**OSI level totals**					
Input/incoming signals	96	22.2	2.3	22.4	3.75
Central decision-making	97	18.2	1.1	18.1	1.9
Output/task performance	97	14.9	2.3	15.3	2.75
General	95	30.0	4.6	30.0	6.0
**OSI aspect totals**					
Underload	96	3.55	1.4	3.5	1.5
High demand	96	30.4	3.0	31.1	4.6
Strictness	96	16.9	2.5	16.5	3.9
External time pressure	97	7.74	1.1	7.8	1.5
Noxious exposures	97	1.83	1.3	1.5	1.5
Threat avoidance/symbolic aversiveness	97	10.2	2.1	10.0	3.0
Conflict/uncertainty	96	14.6	2.4	14.0	3.0
**Copenhagen Burnout Index**					
Personal Burnout (A)	97	48.8	18.9	45.8	25.0
Work-related Burnout (B)	97	43.3	20.2	46.4	32.1
Patient-related Burnout (C)	97	34.2	23.6	33.3	33.3
**Gender**		**%**			
Female	44	45.4			
Male	53	54.6			
**Assessed when working during the COVID-19 pandemic**					
Yes (in direct contact with patients suspected to be infected with COVID-19 virus)	21	21.6			
No	76	78.4			
**Area of practice**					
General practice/community medicine	34	35.0			
Surgical/anesthesia/emergency medicine	32	33.0			
Internal medicine/pediatrics/psychiatry/dermatology/combined specialties and subspecialties	31	32.0			

IQR, Interquartile range; Sd, standard deviation; y, years.

Altogether about 22% of the physicians included in the IPD analysis were working in direct contact with patients suspected of being infected with COVID-19 virus, when they completed the surveys. The full univariate data from the physician-specific OSI questionnaire for all the physicians included in the IPD analysis are given in Supplementary material 3.

For those included in the IPD analysis, the total OSI scores were higher ($\bar{x}$ = 87.0 ± 7.8) among the 74 physicians working prior to the COVID-19 pandemic compared to ($\bar{x}$ = 79.2 ± 7.3) among the 21 physicians working during the pandemic (2-sample *t*-test, *p* = 0.000). Full univariate details on each of the stressors assessed via the OSI questionnaire for the physicians working during the pre-pandemic studies and for those who worked during the pandemic, are presented in the Supplements from Belkić and Rustagi (34) and Nedić and Belkić (39), respectively.

The M Log R findings are presented in Table 2 for the total OSI scores in relation to CBI personal, work-related and patient-related burnout scores, with the three covariates: age, gender and whether or not assessment was when working during the COVID-19 pandemic. For personal burnout above the median integer cutpoint of 46, both working during the COVID-19 pandemic and unit change in OSI yielded significant ORs, with an order of magnitude greater *p*-value for unit change in total OSI scores. The OR for work-related burnout above the median integer cutpoint of 46 was more strongly associated with a unit change in total OSI. Albeit not as strong as for age and gender, a unit change in total OSI scores was significantly associated with patient-related burnout surpassing the integer median cutpoint of 33. The ORs were nearly 4 and over 8 for total OSI scores above 88 being related, respectively, to personal and work-related burnout in the adjusted M Log R analysis of Table 2.

**Table 2:** The total Occupational Stressor Index in relation to the Copenhagen Burnout Indices among the physicians with individual participant data: multivariable logistic regression analysis.

	**Personal burnout (A)** > **46** ***N =*** **95**			**Work-related burnout (B)** > **46** ***N =*** **95**			**Patient-related burnout (C)** > **33** ***N =*** **95**		
	**OR**	−**95% CI**	+**95% CI**	**OR**	−**95% CI**	+**95% CI**	**OR**	−**95% CI**	+**95% CI**
Age	1	0.92	1.08	1.06	0.97	1.16	1.11 (*p* = 0.02)	1.01	1.22
Gender	1.67	0.57	4.93	3.24 (*p* = 0.045)	1.01	10.4	5.56 (*p* = 0.004)	1.7	18.2
Assessed when working during the COVID-19 pandemic	6.24 (*p* = 0.04)	1.09	36	3.24	0.51	20.4	3.04	0.52	18
Total OSI (unit change)	1.11 (*p* = 0.003)	1.03	1.18	1.17 (*p* = 0.0001)	1.08	1.26	1.07 (*p* = 0.03)	1.01	1.15
Model χ^2^	12.5 (*p* = 0.01)			21.9 (*p* = 0.0002)			18.1 (*p* = 0.001)		
Age	1.01	0.93	1.1	1.08	0.99	1.18	1.10 (*p* = 0.04)	1	1.21
Gender	1.71	0.57	5.13	3.48 (*p* = 0.04)	1.03	11.8	4.70 (*p* = 0.009)	1.45	15.2
Assessed when working during the COVID-19 pandemic	3.93	0.76	20.3	1.88	0.34	10.5	2.1	0.38	11.6
Total OSI > 88 (cutpoint for urgent intervention)	3.75 (*p* = 0.01)	1.28	11	8.17 (*p* = 0.0008)	2.36	28.3	1.61	0.54	4.78
Model χ^2^	8.40 (*p* = 0.08)			15.2 (*p* = 0.004)			14.0 (*p* = 0.007)		

Gender is coded as 1 = female, 2 = male; Assessed when working during the COVID-19 pandemic 0 = no, 1 = yes, in direct contact with patients suspected to be infected with COVID-19 virus.

CI, confidence interval; OR, odds ratio; OSI, Occupational Stressor Index.

### 3.2 Queries from the OSI questionnaire yielding multivariable associations with burnout

In Table 3, the queries from the OSI questionnaire yielding multivariable associations with each of the three categories of burnout are displayed. From the OR, 95% CI and *p*-values, the stressors most highly associated with each of the three categories of burnout are identified. These can be used to rank the stressors most highly associated with each of the three categories of burnout.

**Table 3:** Questions from the physician-specific Occupational Stressor Index showing significant or near significant adjusted odds-ratios with personal, work-related and/or patient-related burnout for the physicians included in the individual participant data analysis.

	**Personal burnout (A)** > **46** ***N =*** **97**			**Work-related burnout (B)** > **46** ***N =*** **97**			**Patient-related burnout (C)** > **33** ***N =*** **97**		
	**OR**	−**95% CI**	+**95% CI**	**OR**	−**95% CI**	+**95% CI**	**OR**	−**95% CI**	+**95% CI**
**C. Work hours and scheduling**									
Usual # workdays/week				3.46 (*p* = 0.01)	1.32	9.03			
Usual # work hours/week				1.03 (*p* = 0.04)	1.002	1.05			
Called/emailed during free time about patients or other work				1.98 (*p* = 0.003)	1.26	3.12	1.75 (*p* = 0.02)	1.10	2.79
Insufficient work-free, paid vacation (GH6)	1.71 (*p* = 0.086)	0.92	3.17	1.84 (*p* = 0.048)	1.00	3.40			
Infrequent rest breaks				3.26 (*p* = 0.03)	1.11	9.57			
Number of hours without even a short rest break	2.77 (*p* = 0.05)	0.98	7.89	1.87 (*p* = 0.088)	0.90	3.86			
	**Personal burnout (A)** > **46** ***N** =* **97**			**Work-related burnout (B)** > **46** ***N** =* **97**			**Patient-related burnout (C)** > **33** ***N** =* **97**		
	**OR**	−**95% CI**	+**95% CI**	**OR**	−**95% CI**	+**95% CI**	**OR**	−**95% CI**	+**95% CI**
**D. Salary, possibilities for advancement and recognition**									
Lacks recognition of good work (GU4)				2.97 (*p* = 0.03)	1.07	8.25			
	**Personal burnout (A)** > **46**			**Work-related burnout (B)** > **46**			**Patient-related burnout (C)** > **33** ***N** =* **97**		
	**OR**	−**95% CI**	+**95% CI**	**OR**	−**95% CI**	+**95% CI**	**OR**	−**95% CI**	+**95% CI**
**E. Work conditions**									
	*N =* 92			*N =* 92					
Percent time in office	1.01 (*p* = 0.059)	1.00	1.03	1.02 (*p* = 0.046)	1.00	1.03			
	*N =* 97			*N =* 97					
Radiation exposure	1.91 (*p* = 0.009)	1.17	3.14	1.55 (*p* = 0.076)	0.95	2.53			
Listens to emotionally-disturbing accounts	1.89 (*p* = 0.088)	0.90	3.97						
	**Personal burnout (A)** > **46** ***N** =* **97**			**Work-related burnout (B)** > **46** ***N** =* **97**			**Patient-related burnout (C)** > **33** ***N** =* **97**		
	**OR**	−**95% CI**	+**95% CI**	**OR**	−**95% CI**	+**95% CI**	**OR**	−**95% CI**	+**95% CI**
**F. Mishaps at work**									
Attempted/completed suicide [patient(s) and/or person(s) at work] (GAVOI4)	1.61 (*p* = 0.05)	0.99	2.61	1.62 (*p* = 0.05)	0.99	2.64			
Official complaint against the physician				8.31 (*p* = 0.066)	0.84	81.7			
	**Personal burnout (A)** > **46** ***N** =* **97**			**Work-related burnout (B)** > **46** ***N** =* **97**			**Patient-related burnout (C)** > **33** ***N** =* **97**		
	**OR**	−**95% CI**	+**95% CI**	**OR**	−**95% CI**	+**95% CI**	**OR**	−**95% CI**	+**95% CI**
**G. Time pressure at work**									
Time constraints preclude completion of work tasks	7.73 (*p* = 0.003)	1.95	30.6				3.24 (*p* = 0.07)	0.90	11.7
	**Personal burnout (A)** > **46** ***N** =* **97**			**Work-related burnout (B)** > **46** ***N** =* **97**			**Patient-related burnout (C)** > **33** ***N** =* **97**		
	**OR**	−**95% CI**	+**95% CI**	**OR**	−**95% CI**	+**95% CI**	**OR**	−**95% CI**	+**95% CI**
**H. Problems, restrictions/constraints**									
Problems/deficiencies hinder patient care (OCNFL2)				3.61 (*p* = 0.05)	0.97	13.4			
Understaffing, specifically, hinders patient care				1.98 (*p* = 0.10)	0.86	4.56			
Interruptions from people hamper task performance (OCNFL3)	5.30 (*p* = 0.0006)	2.01	14.0	7.82 (*p* = 0.0002)	2.63	23.3			
	**Personal burnout (A)** > **46** ***N** =* **97**			**Work-related burnout (B)** >**46** ***N** =* **97**			**Patient-related burnout (C)**> **33** ***N** =* **97**		
	**OR**	−**95% CI**	+**95% CI**	**OR**	−**95% CI**	+**95% CI**	**OR**	−**95% CI**	+**95% CI**
**I. Interpersonal interactions and social climate**									
Lacking redress of grievances (GCNFL5)	2.36 (*p* = 0.02)	1.17	4.77	2.06 (*p* = 0.04)	1.03	4.13			
	**Personal burnout (A)** > **46** ***N** =* **97**			**Work-related burnout (B)** > **46** ***N** =* **97**			**Patient-related burnout (C)** > **33** ***N** =* **97**		
	**OR**	−**95% CI**	+**95% CI**	**OR**	−**95% CI**	+**95% CI**	**OR**	−**95% CI**	+**95% CI**
**J. Workload and activities**									
Handling patients who cannot give a history	2.56 (*p* = 0.01)	1.25	5.25	2.59 (*p* = 0.008)	1.27	5.29	2.40 (*p* = 0.03)	1.10	5.24
	*N =* 89			*N =* 89			*N =* 89		
No separate time for non-clinical duties	3.40 (*p* = 0.03)	1.11	10.4	3.25 (*p* = 0.029)	1.11	9.51			
	*N =* 97			*N =* 97			*N =* 97		
Performing tasks that seem pointless (GCNFL7)	3.11 (*p* = 0.038)	1.05	9.21	4.71 (*p* = 0.01)	1.41	15.7			

Multiple logistic regression analysis with age, gender and working or not during the COVID-19 pandemic in direct contact with patients suspected to be infected with COVID-19 virus included as covariates throughout.

CI, confidence interval; GAVOI, threat avoidance on the general level; GCNFL, conflict/uncertainty on the general level; GH, high demand on the general level; GU, underload on the general level; OCNFL, conflict/uncertainty on the output level; OR, odds ratio.

Table 3 begins with sub-section C focusing on Work Hours and Scheduling.

### 3.3 Work hours and scheduling (OSI Sub-section C)

#### 3.3.1 Usual number of work days

The median number of usual workdays per week was 6 for the 97 physicians included in the IPD analysis (Supplementary material 3). Thirty-two (33%) had no weekly free days, i.e., they worked 7 days/week. This variable was associated with an adjusted OR of ~3.5 (95% CI 1.3–9.0) for work-related burnout above the median (>46) (Table 3).

Although there were many cross-sectional studies regarding work hours and physician burnout, most of which did show an association (Supplementary material 2), few of them addressed the number of workdays, *per se*. Among those that did, an investigation carried out in 2009 from Japan of 494 physicians of various specialties, indicated that having only 2–4 days off/month (compared to >8 days off/month) yielded an OR = 3.61 (95% CI 1.09–12.5) for burnout assessed via the MBI, adjusting for gender, marital status, clinical experience, place of work, location and specialty (49). In late 2020, among 226 general practitioners (GPs) from the U.K. who worked >3 days per week, a single-item measure of burnout was marginally higher than for 180 GPs who worked 3 or fewer days/week (32).

As summarized in Table 4, five interventional investigations included a reduction in the number of workdays/week (30, 50–53). A cluster-randomized trial introducing an interrupted schedule with weekend cross-coverage for medical intensivist physicians yielded an adjusted mean decrease in burnout (*p* = 0.003) compared to working continuously for a half-month. Work-home life imbalance and job distress were also significantly lowered with the interrupted schedule. Notwithstanding diminished continuity of care by the intensivists, patient outcomes were not worsened (50). Among the Duty Hour Restrictions for Post-Graduate Medical Education in the U.S., were those implemented in 2011. In three institutions in which this included 1 day off every 7 days, as well as shorter night call shifts, first-year residents in internal medicine showed trends toward diminution in mean incident burnout, emotion exhaustion and depersonalization compared to controls examined in 2008–2009 (53). However, the Authors conclude: “this multi-institutional study found that the implementation of 2011 duty hours standards was not associated with significantly lower rates of burnout among first-year IM (internal medicine) residents, and that unacceptably high rates of burnout persist” (p. 498). Among emergency medicine (EM) and IM residents working in a Minneapolis, Minnesota urban safety net hospital, a number of interventions were implemented in a 5-year cohort study (30). One of these was providing an extra day off for senior residents on ward duty. Significant improvements in empathy perception as a component of burnout, as well as sleep, peer support and nurture of personal relationships were reported during the follow-up period. A single item explicitly assessing burnout *per se*, oscillated between 25% and 35% during the follow-up period. No burnout assessment was reported in retrospective study of surgical residents (51) which indicated that providing a free day from clinical work after night call and other work hour limitations did not diminish their operative case volume. As a simulation study aimed at reducing physician burnout, Geva et al. (52) indicated that shared service could provide days off.

**Table 4:** Intervention studies on work stressors associated with physician burnout.

**1st Author, year published, country**	**Year(s) conducted**	**Participants (N, medical area, level)**	**Study design**	**Work stressor intervention**	**Individual intervention**	**Burnout impact**	**Other impact on MDs**	**Comments/ conclusions**
**C. Work hours and scheduling**								
↓**# Workdays/week**								
Ali et al. (50), 2011, USA	2005–6	45 intensivists: residents, fellow and board certified MDs (39 completed the survey, 13 completed for the 2 conditions)	Cluster randomized	Interrupted weekend cross coverage (standard: continuous ICU responsibility for $\frac{1}{2}$ month)	No	↓ Adjusted mean difference 2.8 (^**^) (“Job burnout score” assessed via scales derived from the US National Study of the Changing Workforce)	↓ Adjusted mean difference: -Work-home life imbalance (^*^) -Job distress (^***^)	“Work schedules where intensivists received weekend breaks were better for the physicians and, despite lower continuity of intensivist care, did not worsen outcomes for medical ICU patients.” (p. 803)
Fergusen et al. (51), 2005, USA	2002–3	Number not explicitly stated, surgery, residents	Retrospective	Free day from clinical work after night call, ↓ mean work hours	No	Not reported	Not reported	“Work-hour limitation can be devised to maximize resident education, optimize patient care, and maintain resident operative volume.” (p. 535)
Geva et al. (52), 2017, USA	Unspecified	Intensive care	Simulation	“2 daytime teams each covered by a different attending &both covered by one over-night on-call attending.” (p. 1138)	No	Not addressed	Not addressed	“A shared service schedule is predicted to improve continuity of care while increasing free weekends and continuity of uninterrupted nonclinical weeks for attendings.” (p. 1138)
Quirk et al. (30), 2021, USA	2014–2019	40-62, IM and 10 EM- IM residents (61–85% response rate)	Cohort	Package of interventions including an extra day off for senior residents on ward duty	Resiliency training, mental health support, wellness 1°care	Varied from 25 to 35% (Single-item measure) ↑ empathy perception ^**^	Improved sleep^*^ peer support^*^ and nurture of personal relationships^***^	This intervention may have impacted the improved outcomes, but its actual contribution cannot be assessed within the package of implemented changes
Ripp et al. (53), 2015, USA	2008–2009, 2011–2012	IM 1st y residents 128 post (68% RR) 111 pre (62% RR)	Pre vs. post resident DHR (different individuals)	DHR with 1 day off every 7 days in 2 of the 3 included hospitals, single days off in the 3^rd^ hospital rather than previous “golden weekends” Shorter night shifts	No	Overall incident BU ↓ 13% ^ε^;Incident EE ↓ 11% NS; Incident DP↓ 14% ^ε^	Not reported	Authors conclude: “this multi-institutional study found that the implementation of 2011 duty hours standards was not associated with significantly lower rates of burnout among first-year IM residents, and that unacceptably high rates of burnout persist.” (p. 498)
**Only** ↓**# work hours/week**								
Barrack et al. (54), 2006, USA	2002 and 2005	55 residents in orthopedic surgery Pre: 21; Post: 34 (RR not known)	Pre vs. post resident WHL (different individuals)	↓ resident work hours (80-94/week pre, 62-73/week post)	No	Post vs. pre: ↓ EE ^ε^ ↓ DP NS; ↑ PA^**^	Post vs. pre: GHQ ≥ 4: 15% vs. 33% NS	“It does seem likely implementing the duty hour standards has been associated with a positive effect on the incidence of burnout among orthopedic surgery residents.” (p. 137)
Gelfand et al. (55), 2004, USA	2003	64, surgery, residents and faculty(RR varied for different groups and portions from 89% to 18.5 %)	Cohort (6-month follow-up)	Limitation to 80 work hours/week	No	Δ EE, DP, PA NS	Not reported	Although resident work hours decreased from 100.7 to 82.6 (*p* < 0.05), significant changes in burnout measures were not seen at follow-up
Goitein et al. (56), 2005, USA	2001 and 2004	118 IM residents post (73% RR post)(~116 pre, RR unspecified)	Pre vs. post resident WHL (different individuals)	WHL	No	Post vs. pre: ↓ High EE by 13% ^ε^; Δ High DP, PA NS	Post vs. pre:↑ Career satisfaction^*^	“Internal medicine residents approve of WHLs overall and report benefits to their wellbeing…they also report negative effects on patient care and resident education.” (p. 2601)
Gopal et al. (57), 2005, USA	2003 and 2004	IM residents: Pre: 121 (87% RR), Post: 106 (74% RR)	Pre vs. post resident WHL (different individuals)	↓ WHL Self-reported: $\bar{x}=$74.6 pre, $\bar{x}=$67.1 post ^**^	No	Post vs. pre: ↓ High EE by 7.5%^**^, Δ High DP NS	Post vs. pre: ↓ Career satisfaction^*^	“Reducing hours may be the first step to reduce burnout but may also affect education and quality of care.” (p. 2601)
Hutter et al. (58), 2006, USA	2003–2004	58 surgical residents, 58 surgical attending physicians (61% RR)	Cohort, Pre vs. post resident WHL	WHL: $\bar{x}=$99.5 pre, $\bar{x}=$78.9 post	No	Post vs. pre: (residents): ↓ High EE by 6% (^*^), ↓ High DP by 3%(ε), Δ PA (NS)	↑Desired achievement and work satisfaction (^*^) (residents)	“Although the mandated restriction of resident duty hours has had no measurable impact on the quality of patient care and has led to improvements for the current quality of life of residents, there are many concerns with regards to the training of professional, responsible surgeons for the future.” (p. 864)
Martini et al. (59), 2006, USA	2003–2005	118, all medical areas, residents (31% RR)	Cohort	WHL	No	41% > MBI cutoff at follow-up (50% prior to implementation) (1st year residents 42.9% vs. 77.3% prior to implementation)	Not reported	“Somewhat lower burnout prevalence was reported among residents after implementation of work hour limits compared with the rates prior to the implementation period. The decrease in burnout prevalence occurred primarily among 1st year residents. Prevalence of burnout increased with hours worked…. Implementing work hour limits appeared to reduce burnout prevalence.” (p. 352)
Isaksson Rø et al. (60) 2008, Norway	2003–2005	185, non-specialists and various specialties (81% participation at follow-up)	Cohort (1-year follow-up)	↓ 1.6 ± 11.4 work hours, ↓ Perceived job stress (Not explicit interventions)	Group or individual counseling	↓ EE and DP (^***^), PA NS	↓ fulltime sick leave (35% at baseline) (6% 1y f/u)	The counseling session is likely to have impacted on work hours and other job stressors. Work hour diminution was independently associated with the reduced emotional exhaustion component of burnout
Shoureshi et al. (61), 2021, USA	2020 (explicitly during the COVID-19 pandemic)	440, urologists (26% response rate)	Cross-sectional	53% ↓ or Δ work hours to manage burnout Other workplace changes are noted but cannot assess simultaneity	Several are noted but cannot assess simultaneity	45% reported that ↓ or Δ work hours was “very effective” to ↓ burnout	Not reported	Reducing work hours was the 4^th^ most effective of 9 interventions for reducing burnout
↓**Emails/calls during free time**								
Quirk et al. (30), 2021, USA	2014–2019	40–62 IM and 10 EM- IM residents (61–85% response rate)	Cohort	Package of interventions including removing after-hours consult pager calls to residents to shift some of their responsibilities to faculty	Resiliency training, mental health support, wellness 1°care	Varied from 25 to 35% (Single-item measure), ↑ empathy perception (^**^)	Improved sleep (^*^), peer support(^*^), nurture of personal relationships (^***^)	This intervention may have impacted the improved outcomes, but its actual contribution cannot be assessed within the package of implemented changes
↑**Rest breaks**								
Hutter et al. (58), 2006, USA	2003–2004	58 surgical residents, 58 surgical attending physicians (61% RR)	Cohort Pre vs. post resident WHL	↓ WHL, $\bar{x}=$99.5 pre, $\bar{x}=$78.9 post, Residents ↑ apt to have lunch ^*^	No	Post vs. pre: (residents) ↓ High EE by 6% (^*^), ↓ High DP by 3%(ε), Δ PA (NS)	↑ Desired achievement and work satisfaction(^*^) (residents)	Together with ↓ work hours, the ↑ likelihood of eating lunch may have contributed to the improved burnout profile and motivation among the residents
Belkić and Savić, Belkić and Nedić (18, 48), 2013 and 2019, Unspecified	Unspecified	Case study, senior psychiatrist at an academic medical center	Clinical intervention	10-min undisturbed break time between patients	Consultation with occupational psychiatrist	Mini-Olbi Exhaustion assessed at baseline only	Returns to work, ↑ self confidence and self-care	Along with other interventions as per the occupational psychiatrist's recommendation, overall clinical improvement
Shea et al. (68), 2014, USA	2009–2010	106 IM, interns	RCT, 4-week blocks with vs. without	5-hour protected sleep 12:30-5:30 (no phone/beeper) (*N =* 88) (covered by night float resident) vs. control (*N =* 91)	No	NS differences in end of rotation Δ EE, DP, PA (MBI) for INT vs. control blocks	↑ Sleep oncall, if protected (^**^), ↑ Sleep oncall ∝↓EE, DP (^**^)	These rest breaks are quite different (longer, scheduled at a fixed time foreseen at “protected sleep” during night shift), from those during usual working hours
Ireland et al. (38), 2017, Australia	Not explicitly stated	44 EM rotation (10 weeks), interns, (100% response rate)	RCT	Extra hour of break time in the middle of the day/week(treated as “control”) (*N =* 21)	Mindfulness education and practice (*N =* 23) Intervention	Δ Mean CBI A&B: ↑0.16 (controls), ↓0.2 (Intervention)	Δ Mean PSS: ↑0.06 (controls), ↓0.36(Intervention)	It would have been of interest to assess the combined effect of the mindfulness intervention and the extra break time
**D. Salary, possibilities for advancement**								
↑**Recognition of good work**								
Angelopoulou and Panagopoulou (70), 2020, Greece	Not explicitly stated	36, 1° care, residents, senior physicians and nurses (volunteers recruited from advertisement)	Cohort (Pre- and post intervention)	2-h group professional recognition session: self: identify and share management of a clinical case; positive feedback from group and from patients	No	Not reported	↑ positive emotions (^***^)	“First [reported] intervention targeting professional recognition implemented in health-care settings” (p. 1)
Chang et al. (8), 2023, USA	2021–2022	84, EM, residents (response rate: 100% pre, 86% post)	Cohort (Pre- and 6M post intervention)	Peer-to-peer recognition program: “Bonusly” platform with rewards	Recognition tokens could be redeemed as gifts, personalized prizes	–PFI BU NS; –Recognized for accomplishments (^*^)	Residency fosters supportive WE(^*^)	The Authors recommend further longitudinal study examining peer-to-peer recognition during the entire 4 years of residency training
Quirk et al. (30), 2021, USA	2014–2019	40–62 IM and 10 EM- IM residents (61–85% response rate)	Cohort	Package of interventions including a newsletter “celebrating resident achievements” (p. 690)	Resiliency training, mental health support, wellness 1°care	Varied from 25 to 35% (Single-item measure), ↑ empathy perception (^**^)	Improved sleep (^*^), peer support(^*^) and nurture of personal relationships(^***^)	This intervention may have impacted the improved outcomes, but its actual contribution cannot be assessed within the package of implemented changes
**E. Work conditions**								
**Improved office conditions**								
Belkić and Savić, Belkić and Nedić (18, 48), 2013 and 2019, Unspecified	Unspecified	Case study, senior psychiatrist at an academic medical center	Clinical intervention	Pre = shares windowless office, looks for another office to see patients, Post = own office with window	Consultation with occupational psychiatrist	Mini-Olbi Exhaustion assessed at baseline only	Returns to work, ↑ self confidence and self-care	Along with other interventions as per the occupational psychiatrist's recommendation, overall clinical improvement
Larsen et al. (74), 2021, USA	2020 (explicitly addresses the COVID-19 pandemic)	98, radiologists and referring clinicians, attendings and trainees	On-going participation/ Feedback from focus groups	Process of redesign of radiology reading rooms with “purposeful space” and separate areas for various work activities	No	Of concern in the redesign process, but not explicitly reported	Various needs met in part or completely	Conclusions are limited regarding the actual impact of this on-going redesign project on the radiologists and referring clinicians
Quirk et al. (30), 2021, USA	2014–2019	40–62 IM and 10 EM- IM residents (61–85% response rate)	Cohort	Package of interventions including attention to lighting in rooms used by residents	Resiliency training, mental health support, wellness 1°care	Varied from 25 to 35% (Single-item measure), ↑ empathy perception (^**^)	Improved sleep (^*^), peer support(^*^), nurture of personal relationships (^***^)	This intervention may have impacted the improved outcomes, but its actual contribution cannot be assessed within the package of implemented changes
**Addressing exposure to that which is emotionally disturbing**								
Monette et al. (78), 2020, USA	2020 (explicitly addresses the COVID-19 pandemic)	EM clinicians, 29 attending MDs, 6 residents, 33 non-physician practitioners (76% response rate to survey)	Invitational	Role-based, weekly 1-h debriefings via Zoom. Focus: empathy and normalizing reactions		Not reported	98% endorsement: “facilitators created a safe environment”	Although no actual burnout measures, these are the kinds of emotional amelioration that are sought vis-à-vis the emotional burden. Such interventions are needed when physicians are faced with heavily disturbing occurrences
Funding et al. (37), 2023, Denmark	2021	43, hematology-oncology, residents (56% participation rate)	1st participants vs. “waiting list” baseline and 12-weeks later	2-day course–formal training with communication exercises, focused on serious illness		CBI A, B and C: Pre-vs-post Int: NS, Post Int vs. controls: NS, Post Int vs. controls: Moderate-severe BU: 38% vs. 31%	2 items in Self-efficacy: INT vs. controls (^*^) -work with own barriers to communicating existential issues and uncertainty with patient	“A mandatory course of formal training can increase physician self-efficacy in serious illness communication and alter clinical practice and perception of roles. The high level of burnout among physicians in hemato-oncology calls for institutional interventions in addition to training” (p. 547)
Belkić and Savić, Belkić and Nedić (18, 48), 2013 and 2019, Unspecified	Unspecified	Case study, senior psychiatrist at an academic medical center	Clinical intervention	Temporary relief from EM duty to ↓ exposure to patients at high suicide risk	Consultation with occupational psychiatrist	Mini-Olbi Exhaustion assessed at baseline only	Returns to work, ↑ self confidence and self-care	Along with other interventions as per the occupational psychiatrist's recommendation, overall clinical improvement
Soto and Rosen (79), 2003, USA	1999	Case study, pediatric anesthesia, resident	“Pediatric death: Guidelines for the Grieving Anesthesiologist”: Military strategy “BICEPS”: *Brevity:* Dealing with the stressor will be brief and focused. *Immediacy:* Feelings of grief and/or guilt should be confronted soon after the traumatic event or as soon as symptoms are recognized. *Centrality:* Discussions should take place with all affected health care staff in a central location in an organized fashion. *Expectancy:* It should be clear that the expectation will be that affected individuals will be returned to work, and that a means of returning to normal productivity should be outlined (for instance, increasing supervision or decreasing patient acuity). *Proximity:* Discussions and treatment should take place near the place of work to maintain friendships and bonding. Sending a worker home for a week can increase feelings of guilt and alienation. *Simplicity:* Discuss and treat only the current problem, and avoid medications or complicated recovery regimens. (pp. 276–277)					
**F. Mishaps at work**								
**Support with patient suicide**								
Belkić and Savić, Belkić and Nedić (18, 48), 2013 and 2019, Unspecified	Unspecified	Case study, senior psychiatrist at an academic medical center	Clinical intervention	Temporary relief from EM duty to ↓ exposure to patients at high suicide risk	Consultation with occupational psychiatrist	Mini-Olbi Exhaustion assessed at baseline only	Returns to work, ↑ self confidence and self-care	Along with other interventions as per the occupational psychiatrist's recommendation, overall clinical improvement
Agrawal et al. (82), 2021, USA	2020	57, psychiatry, residents 10 had patient suicide (97% initial response rate baseline, lower for various portions)	Cohort	Postvention protocol (adherence in 9 of 10 residents): Notify supervisor, discuss emotional impact, coverage of work duties if needed, offer psychotherapy, offer to meet with peer/faculty with similar experience (most often deemed “extremely helpful”) and longer term f/u prn (see paper for full details)		EE, DP, PA NS difference between those with vs. no patient suicide	NS difference between those with vs. no patient suicide for Work empowerment, general health.	“The postvention protocol was helpful to residents and potentially effective at mitigating the psychological and professional consequences of patient suicide. Study findings may inform standardization of postvention protocols among psychiatry training programs.” (p. 262)
**Support with official complaint against physician**								
Doehring et al. (33), 2023, USA	Not explicitly stated (explicitly addresses the COVID-19 pandemic)	17, academic and community EM departments, (61% participation rate) facing malpractice lawsuit	Cohort pilot study	Litigation peer support: discuss legal aspects, impact on work, support through shared experiences, coping strategies		Single-item MBI, NS pre vs. post Int	Acute distress symptoms, NS pre vs. post Int	“Despite increasing burnout in the specialty of emergency medicine…during the study time frame, burnout did not worsen in participants…[such that] formal peer support offered by EM groups can be an effective option to normalize the experience of being sued, promote wellness, and benefit physicians who endure the often long and stressful process of a medical malpractice lawsuit” (p. 205)
**G. Time pressure at work**								
↓**Time constraints that preclude completion of work tasks**								
Jantea et al. (96), 2018, USA	2014	208 IM residents, 39 core faculty members	Cohort	A 50/50 block schedule with “clinic buddy” during entire residency: alternates inpatient and outpatient rotations, focuses on continuity during outpatient rotations, ↓ conflicting responsibilities.	No	Not reported	^*^ Improved resident relation with clinic patients and with other staff (faculty assessment)	“This 50/50 model minimized inpatient distractions in clinic and increased perceived time for learning. Residents reported improved sense of patient ownership, relations within the multidisciplinary team, and integration into the clinic system. Intervisit continuity was preserved, visit continuity was slightly decreased, and patient outcomes were not impacted in this model” (p. 223)
Quirk et al. (30), 2021, USA	2014–2019	40–62 IM and 10 EM-IM residents (61-85% response rate)	Cohort	Package of interventions including allowing more time to residents for patient visits during clinic	Resiliency training, mental health support, wellness 1°care	Varied from 25 to 35% (Single-item measure) ↑ empathy perception (^**^)	Improved sleep (^*^), peer support(^*^), nurture of personal relationships (^***^)	This intervention may have impacted the improved outcomes, but its actual contribution cannot be assessed within the package of implemented changes
**H. Problems, restrictions/constraints**								
**Improved staffing**								
Gregory et al. (100), 2018, USA	Not explicitly stated	60–70 1° care physicians, (53–62% response rate)	8 centers: 4 Intervention and 4 control, 3M and 6M f/u	Replace dyad: physician + CMA, with 2 providers + 3 CMAs	No	–↓ EE ^*^ with Int; –High DP at 6M: 12% Int vs. 23% control	–High self-efficacy at 6M: 94% Int vs. 78% control	The Authors conclude: “Although difficult to implement and evaluate, organizational changes designed to reduce burnout have the potential to improve physicians' work experience.” (p. 348)
Quirk et al. (30), 2021, USA	2014–2019	40–62 Int Med and 10 EM-Int Med residents (61–85% response rate)	Cohort	Package of interventions including “jeopardy resident coverage,” house officer is called in to replace a resident at short notice for vital personal life events	Resiliency training, mental health support, wellness 1°care	Varied from 25 to 35% (Single-item measure), ↑ empathy perception (^**^)	Improved sleep (^*^), peer support(^*^), nurture of personal relationships (^***^)	This intervention may have impacted the improved outcomes, but its actual contribution cannot be assessed within the package of implemented changes
↓**Interruptions that hamper task performance**								
Kapoor et al. (104), 2020, USA	2018	11 physicians and advanced nurse practitioners (79% response rate)	Retrospective 12 m f/u	Geographical cohorting model for critical care physician rounding: ICU teams of staff physicians, residents, students geographically limited to 1 nursing unit according to expertise and interest	No	Not reported	80% agreed re: ↓ interruptions with geographical cohorting	At f/u: Nine of 11 providers agreed that geographical cohorting improved quality of care. Similar ICU utilization, ↓ central line, clostridial and urinary tract infection (^*^) “Geographical cohorting improves coordination of care, physician workflow, and critical care quality metrics in very large ICUs.” (p. 1)
Larsen et al. (74), 2021, USA	2020 (explicitly addresses the COVID-19 pandemic)	98, radiologists and referring clinicians, attendings and trainees	Descriptive	Redesign of radiology reading rooms → ↓ interruptions, especially the deep reading space for tasks that “require sustained periods of uninterrupted focus” (p. 111)	No	Not explicitly reported	Various needs met in part or completely	Conclusions are limited regarding the actual impact of this on-going redesign project on the radiologists and referring clinicians
Lapointe et al. (105), 2018, USA	Not explicitly stated	25, IM residents (random selection, % response rate not reported)	Cohort (Pre- and 4m post Intervention)	Text paging EHR-based system: ↓ Interruptions to patient care ^**^	No	Not reported	“Stress and frustration” ↓ in 22 (88%)	“Text paging among medical caregivers and internal medicine residents through EHR-associated communication reduced patient care and educational interruptions. It saved time spent sending pages, answering unnecessary pages and it improved resident's subjective stress and satisfaction levels.” (p. 1)
Luu et al. (31), 2023, USA	2019	17, EM residents (71% response rate)	Cohort 4m f/u	EHR messaging system for non-urgent communication	No	Reported impact of # non-urgent calls on BU (single query): NS	↓ # non-urgent calls ^***^	The system diminishes unnecessary non-urgent calls from other HP, improving workflow in the ED
**J. Workload and activities**								
**Separate time for non-clinical activities**								
Jones et al. (108), 2022, USA	2020 (explicitly during the COVID-19 pandemic)	Eight acute care surgeons at a trauma and tertiary care center	Cohort 1-y f/u	Protected academic time [plus continuous call ↓ to 12h (pre-Int 24h)]	No	MBI ↓ 12.5%, EE ↓ 28%, DP ↓ 38%, PA↑ 12.5%	Improved sense of control, reward, fairness, values	“Improvements were noted in surgeon and family groups alike, signifying both subjective improvements and observed change in the surgeons' behavior, without compromising clinical productivity.” (p. 439)
Quirk et al. (30), 2021, USA	2014–2019	40–62 IM and 10 EM- IM residents (61–85% response rate)	Cohort	Package of interventions including “improved residents' research skills by: adding protected time in residents' schedules to complete human subjects training, matching residents with research mentors, and making it easier for them to access online research resources. “ (p. 692)	Resiliency training, mental health support, wellness 1°care	Varied from 25 to 35% (Single-item measure), ↑ empathy perception (^**^)	Improved sleep (^*^), peer support(^*^), nurture of personal relationships (^***^)	This intervention may have impacted the improved outcomes, but its actual contribution cannot be assessed within the package of implemented changes
↓**Seemingly pointless/illegitimate tasks**								
Contratto et al. (110), 2017, USA	2014–2015	Seven IM 1° care attendings at academic center, 100% participation	Cohort 4 m f/u	Clerical support person hired and trained to enter physician orders in the EHR and conducted previsit plan	No	~30% ↓ burnout ≥ weekly, 28% ↓ callousness weekly or monthly	~30% ↑ satisfaction with personal balance and good QoL	Among the representative statements from the participating physicians in focus group: “I feel like I'm taking better care of my patients because I'm not doing everything.” (p. 366)
Mishra et al. (111), 2017, USA	2016–2017	12 IM, 6 family practice, 1° care (75% participation)	RCT dual-balanced crossover	Contracted medical scribes to assist with EHR	No	Not reported	Scribed periods ∝↓ self-reported after-hours EHR documentation and ↑ spending > 75% of visit interacting with the patient	Although not explicitly assessed in the present paper, the Authors foresee that by reducing the EHR burden faced by primary care physicians, burnout could potentially be reduced
Shoureshi et al. (61), 2021, USA	2020 (explicitly during the COVID-19 pandemic)	440, urologists (26% response rate)	Cross-sectional	11% hired a scribe, other workplace interventions are noted but cannot assess simultaneity	Several are noted but cannot assess simultaneity	63% reported that hiring a scribe was “very effective” to ↓ burnout	Not reported	Hiring a scribe was the most effective of 9 interventions for reducing burnout

Significance levels: ε: 0.05 < p < 0.10, ^*^ 0.01 < p < 0.05, ^**^ 0.001 < p < 0.01, ^***^p < 0.001, NS, statistically non-significant &/or p ≥ 0.10.

CBI, Copenhagen Burnout Inventory (Personal burnout = A), (Work-related burnout = B), (Patient-related burnout = C); CMA, certified medical assistant; DHR, duty hour restrictions; DP, depersonalization; EE, emotional exhaustion; EHR, electronic health record; EM, emergency medicine (or emergency work); f/u, follow-up; h, hour(s); GHQ, General Health Questionnaire; HP, health providers; ICU, intensive care unit; IM, Internal Medicine; INT, intervention; M, month(s); MBI, Maslach Burnout inventory; PA, personal accomplishment; prn, as needed; PFI, Professional Fulfillment Index; PSS, Perceived Stress Scale; QoL, Quality of life; RCT, randomize controlled trial; RR, response rate; WHL, work hour limitations (as per the U.S. Accreditation Council for Graduate Medical Education); $\bar{x}$, mean.

#### 3.3.2 Work hours per week

Altogether 37 of the 97 physicians included in the IPD worked over 80 h/week, which was also the mean (Supplementary material 3). With each hour of increased weekly work time, there was a greater likelihood of work-related burnout above the median (*p* = 0.04) (Table 3).

In 2003, Work Hour Restrictions (WHR) were mandated to be 80 h maximum for Post-Graduate Medical Education in the U.S. Several publications assessed burnout among residents in relation to number of work hours/week (54–59). As indicated in Table 4, some of these studies assessed different groups of residents prior to and after the WHR were implemented. Diminution in burnout rates and its components using the MBI was seen in nearly all the studies. Two investigations among practicing physicians outside post-graduate training (60, 61) also suggested that work hour limitations helped diminish burnout.

#### 3.3.3 Contacted during free time

The majority of the 97 physicians included in the IPD analysis were contacted occasionally or frequently by phone and/or email outside duty time about clinical care of patients or other job-related issues. This was associated with an almost two-fold increased likelihood of work-related burnout >46 and nearly as high a chance for patient-related burnout scores above the median, as per the adjusted analysis (Table 3).

The searched literature on this topic among physicians mainly yielded publications regarding electronic health records (EHR) and other documentation issues. These will be considered in Section 3.10 regarding workload and activities. One study (62) carried out among 106 palliative care providers captured the essence of this stressor. Namely, “work intrusiveness” when with family or friends or attending to domestic obligations. This was correlated (*p* = 0.005) with work-related burnout. Physicians, however, were not included in that study.

Among the interventions implemented for IM and EM residents in the 5-year cohort study (30) was removal of after-hours consult pager calls, with transfer of some responsibilities to faculty during that time. As noted, significantly improved empathy perception as a component of burnout, as well as improved sleep, peer support and nurture of personal relationships were reported during the follow-up period (Table 4).

#### 3.3.4 Insufficient work-free, paid vacation

There was substantial variation in the scores regarding work-free paid vacation among the 97 physicians, with a median score of 1 (3–4 weeks/year). Insufficient work-free vacation was related to personal burnout and more strongly to work-related burnout in the adjusted multi-variable IPD analysis (Table 3).

Many cross-sectional publications prior to and during the COVID-19 pandemic concordantly reported a direct or indirect association between insufficient vacation time free from clinical and other work obligations and physician burnout (Supplementary material 2). Among the most recent studies (63), of 3,024 U.S. physicians, taking more than 3 weeks of vacation and having full EHR coverage had an adjusted OR = 0.66 (95% CI: 0.45–0.98) and 0.74 (95% CI: 0.63–0.88), respectively, for MBI. Progressively elevated adjusted OR's for MBI were found with greater vacation time spent on patient-related work, up to OR = 1.9 (95% CI: 1.36–2.73) if >90 min/day. Among 498 women physicians, 66.3% reported an increase in at least one work-related activity while on vacation during the COVID-19 pandemic. The most frequently endorsed strategy for reducing work engagement during vacation was formal discussions at the workplace/department (64). Vacation time was considered the highest reform priority in a study of 525 Canadian family physicians (65). Notwithstanding the importance of work-free vacation for physicians, no intervention studies were found addressing this issue.

#### 3.3.5 Insufficient rest breaks

Approximately two-thirds of the 97 physicians included in the IPD analysis lacked genuine rest breaks free from work obligations. The vast majority had only short rest breaks during work hours. The more infrequent the rest breaks, the greater the likelihood of work-related burnout (OR = 3.26, 95%CI 1.1–9.6). The longer time without even a short rest break, the greater the likelihood not only for burnout (B), but also for personal burnout (OR = 2.77, 95% CI 0.98–7.89) (Table 3).

There are some cross-sectional studies in which rest-breaks were considered in relation to burnout (Supplementary material 2). Among emergency department health professionals in a French university hospital, including 28 physicians, lacking adequate time for eating or completely skipping meals was a significant risk factor for burnout (66). In the above-described study of GPs in the U.K., taking ≥2 breaks in sitting per hour was associated with a lower score on the single-item measure of burnout (*p* = 0.007) (32). During the COVID-19 pandemic, even when rest breaks were formally available, the chance to actually rest and recover during such times was often compromised. The latter was associated with work-related burnout (*p* < 0.01) in a study of National Health Service staff in the U.K. (67).

Published intervention studies directly impacting rest breaks and burnout among physicians are sparse. As indicated in Table 4, indirect evidence can be gleaned from Hutter et al. (58) that the greater likelihood of eating lunch after implementation of WHR could have contributed to improved burnout profile and indices of motivation among surgical residents. One of the immediately implemented measures that may have contributed to overall clinical improvement in the case study of an exhausted psychiatrist was at least 10 min of undisturbed break time between outpatients (18, 48). A randomized, controlled trial providing an undisturbed 5-h sleep period to internal medicine interns was associated with longer sleep which, in turn, was associated with lowered emotional exhaustion and depersonalization (*p* < 0.01). However, there were no significant differences in MBI burnout indices at the end of the 4-week intervention and control periods (68). An extra hour of rest breaks per week was not associated with changes in CBI at 10-week follow-up among interns during their emergency department rotation (38).

#### 3.3.6 Total OSI high demand

Most of the queries in Section 3.3 of the OSI questionnaire contribute to the General Level of the High Demand aspect of the OSI. For the present IPD analysis, with age, gender and working during the COVID-19 pandemic included as covariates, total High Demand scores yielded an OR (± 95% CI) = 1.20 (1.01 – 1.42) (*p* = 0.03) for Personal Burnout >46, and 1.25 (1.05 – 1.48) (*p* = 0.01) for Work-related Burnout >46.

### 3.4 Salary, possibilities for advancement and recognition (OSI Sub-section D)

#### 3.4.1 Lacks recognition of good work

The median for recognition of good work was 0.5, corresponding to “Yes, to some extent” for the 97 physicians for whom there are IPD. When such recognition is lacking, the likelihood of work-related burnout is nearly three-fold among these physicians (Table 3).

The importance of recognizing physicians' work has been widely acknowledged. A cross-sectional study of 2,145 general practitioners carried out during mid-2022 in Chongqing, China, revealed significant multivariable associations between receiving sufficient recognition from patients (as well as from the medical team) and lower scores on each of the three components of the MBI. These findings suggested that lack of recognition contributed to burnout among the physicians evaluated in the study (69).

Three publications were identified in which interventions were aimed at enhancing professional recognition among physicians. As noted in Table 4, in the first of these, a pilot study (70) carried out among 36 primary care professionals in Thessaloniki, Greece, the professional recognition intervention was associated with increased positive emotions and lower arousal emotions at follow-up. Burnout, *per se*, was not assessed. The Authors state that to their knowledge, “this is the first intervention targeting professional recognition implemented in health-care settings” (p. 950). A peer-recognition program completed by 72 EM residents in the U.S. yielded improved feelings of recognized accomplishments and perception of a comfortable, supportive work environment at 6-month follow-up. However, no significant changes in burnout as assessed by the Stanford Professional Fulfillment Index were noted in association with the intervention (8). For the third study (30), the “package of interventions” included a newsletter “celebrating resident achievements” (p. 690). As noted, empathy perception was significantly improved, as were sleep, peer support and nurture of personal relationships.

#### 3.4.2 Total OSI underload

Lack of recognition of good work, as well as the other items in Sub-section D of the OSI questionnaire for MDs are used to generate the total Underload score in the OSI. These queries are all part of the General level of the Underload aspect, and are quite akin to the low reward component of the Effort Reward Imbalance model (14, 71). With age, gender and working during the COVID-19 pandemic as covariates, for the present IPD analysis, total Underload scores showed an OR (± 95% CI) = 1.61 (1.11 – 2.34) (*p* = 0.01) for Work-related Burnout >46, and 1.54 (1.02 – 2.32) (*p* = 0.04) for Patient-related Burnout >33.

### 3.5 Work conditions (OSI Sub-section E)

#### 3.5.1 Office conditions

As noted in Supplementary material 3, there were many untoward office conditions including cramped office space, several people sharing an office, the majority not even having their own desk, with ten of the 97 physicians working in windowless offices. The greater the percent of work time spent in the office, the greater the likelihood of personal and work-related burnout in the multivariable analysis (Table 3).

Some cross-sectional investigations included assessment of office conditions in relation physician burnout. Among 1,498 anesthesiologists and intensivists in France carried out in 2018, having less “personal space” showed a strong relation (*p* = 0.000) with personal and work-related CBI scores >50 (35). The impact of untoward office conditions became even more salient during the COVID-19 pandemic. An investigation carried out in 2020 revealed a significant association between accessing daylight during work and reduced burnout in 406 physicians and nurses (72). The importance of the physical environment, in particular, separate space to “step away from work demands” was also highlighted in a mixed methods study of physicians and other emergency health care workers published in 2023 (73).

No larger-scale intervention studies among physicians regarding office conditions and burnout were identified. However, among the immediate interventions reported in the case study of the exhausted psychiatrist who had shared a windowless office with a colleague, was to provide her a separate office with a window. Thereby, she could also see her patients without having to search for another room, plus ensuring that she had the chance to take the above-described rest breaks (18, 48). In Larsen et al. (74), focus groups of radiologists, referring clinicians and trainees gave their qualitative and quantitative feedback during the process of redesigning radiology reading rooms with “purposeful space” and separate areas for various work activities. Burnout was a noted consideration, but its explicit assessment was not reported. In the above-cited multi-faceted intervention study (30), attention was given to lighting in rooms used by residents, with the mentioned improvements in empathy perception, sleep, peer support and nurture of personal relationships (Table 4).

#### 3.5.2 Radiation exposure

Approximately 30% of the 97 physicians reported being exposed to radiation during work, 19 (66%) of whom did not wear a radiation badge. Exposure to radiation was associated in multivariable analysis with work-related burnout and more strongly with personal burnout (Table 3).

Relatively few publications were found in which radiation exposure and burnout are addressed with regard to physicians, as listed in Supplementary material 2. Among these, in the most recent study (75) carried out among 215 residents in internal medicine, concerns about burnout and about radiation exposure were significantly greater among the female physicians. However, no assessment was reported in that or the other studies regarding the relation between radiation exposure and burnout.

The exposure queries in part E of the OSI questionnaire concerning work conditions refer not only to the purely physical aspects, but also to attendant hazards/broader aversiveness. Radiation exposure is scored within the OSI as part of hazardous task performance, within the threat avoidant vigilance/symbolic aversiveness aspect of the OSI.

#### 3.5.3 Listening to emotionally-disturbing accounts

The aversiveness of listening to emotionally-disturbing accounts is included on the input level of threat avoidant vigilance. The median response to this query among the 97 physicians was 1, corresponding to occasionally listening to such accounts. Twenty-three of these physicians answered that they frequently did so. Albeit with rather wide 95% CI, the adjusted OR was 1.89 for personal burnout associated with this stressor (Table 3).

Concordant findings were reported in other cross-sectional studies. Among 285 psychiatrists and 326 non-psychiatrist Paris hospital physicians, “emotional demands” (a dichotomous variable) were associated with personal and work-related burnout and with an expanded definition of burnout (C) referring to all interpersonal interactions (76). The OR's were between 3 and 4. Exposure to “high emotional demands” among 278 Sri Lankan post-graduate physicians also showed adjusted ORs between 3 and 4 for burnout (A), (B) and (C) (3). Oncologists, whose subspecialty area entails heavy exposure to that which is emotionally disturbing, are reported to be particularly vulnerable to burnout (77).

In the physician-specific OSI questionnaire, the query is intentionally left open as to what the respondent considers emotionally disturbing. This is scored according to the reported frequency of exposure, broadly reflecting intensity. The key is to recognize when the emotional toll has become excessive, and to intervene with protective measures (18). In Table 4, four different studies are reviewed in relation to this stressor. Therein, various interventional strategies are presented and their impact on burnout and other measures of physician wellbeing are described. Among these was a 2-day course for hematology-oncology residents focused on serious illness communication, which was not associated with a noteworthy decline in burnout (A), (B), or (C) compared to baseline nor compared to controls. Self-efficacy was, however, improved in relation to communicating existential issues and uncertainties with patients. The Authors conclude: “The high level of burnout among physicians in hemato-oncology calls for institutional interventions in addition to training” (37) (p. 547). Although burnout *per se* was not evaluated, a Zoom-based debriefing program implemented among 35 emergency department residents and attending physicians was considered “an acceptable and useful approach to support emotional wellbeing during the coronavirus pandemic” (78) (p. 88). Informed by successful strategies among infantry soldiers, recommendations have been put forward for anesthesiologists faced with pediatric deaths (79).

### 3.6 Mishaps at work (OSI Sub-section F)

The aversiveness which physicians may encounter becomes more explicit in Section 3.6 of the OSI questionnaire. The topic of suicide is broached therein, firstly among patients and then among colleagues and other coworkers. This combined exposure to suicide contributes to threat avoidant vigilance on the general level. Altogether, 35 (36%) of the 97 physicians had some work-related exposure to suicide, associated with an increased likelihood of personal and work-related burnout (Table 3).

#### 3.6.1 Attempted or actual patient suicide

Nineteen of the 97 (20%) of physicians either heard about or had patients who attempted or committed suicide. Eight physicians had actually cared for one or more such patients.

We found relatively few articles addressing the toll on physicians of caring for such patients. A cross-sectional investigation among 368 hospital healthcare providers in Malaysia (80) examined attitudes toward patients who are suicidal. Only the personal accomplishment component of the MBI showed an association with understanding and willingness to aid patients who had attempted suicide. A study of 90 psychiatrists attending a conference in the U.S. indicated that after the suicide of a patient, the majority sought support from a colleague and/or from a family member or friend (81).

The earlier-cited case study illustrates the potential impact of patient suicide even for an experienced psychiatrist (18, 48), who subsequently became so exhausted that she needed major help to be able to return to work. Among the implemented measures was a temporary relief from work in the emergency department to diminish her exposure to the most acutely-ill psychiatric patients (Table 4). A protocol was evaluated among psychiatry residents, ten of whom had experienced patient suicide during their residency (82). The program included a primary response of the supervisor to meet and discuss the physician's initial emotional response, medico-legal issues and coverage of work duties, if needed. A more involved secondary response from the training director offered referral psychotherapy and other further support, group reflections and meeting with other colleagues who had similar experience. Nine of the ten residents followed the protocol. The most helpful was to talk with attending physicians who had faced such adverse events. Findings on the MBI did not differ between the 39 residents who did not have patient suicide vs. the ten who did.

#### 3.6.2 Attempted or actual suicide of person(s) with whom one works

Nearly 25% of the 97 physician stated that there had been one or more suicide attempts or completed suicide among colleagues or staff at work. In the majority of cases, the person was known, and there were altogether sixteen actual suicides reported.

Notwithstanding the large body of literature on suicide among physicians (83), until recently there has been a surprising lack of attention to its impact on colleagues. As poignantly described nearly two decades ago: “The suicides that had already occurred were never discussed openly, no one undertook a publicly acknowledged serious analysis of the causes, and no other clear safeguards were put into place. The deaths were simply accepted as a fact of medical life” (84) (p. 2474). Moreover, it has been stated: “no silence is as profound as that which greets the news of a suicide in the medical community” (85) (p. 247).

In 2021, the American Medical Association published a comprehensive toolkit for responding to a physician's suicide (86), which includes support to close colleagues, insofar as the physician's family granted permission to share that information. The importance of help to coworkers after suicide is well-recognized, especially by nurses (87, 88). However, no intervention studies were found addressing physicians after an attempted or completed suicide of a colleague or staff at work. Two U.S. programs have been described that appear to improve physician-to-physician suicide risk assessment and support (89, 90).

#### 3.6.3 Official complaint against the physician

About 20% of the 97 physicians had ever testified in court in an official capacity and/or had received an official complaint about their work. The latter was associated with an elevated risk of work-related burnout in the IPD analysis (Table 3).

Many cross-sectional studies have indicated that untoward patient outcomes, especially if followed by medico-legal processes are linked to physician burnout (Supplementary material 2). These “adverse events” impact profoundly upon the physician, who has been termed the “second victim” and who is also in need of and reportedly helped by peer support (91–93). An intervention study included 17 EM physicians with active lawsuits who participated in Group Peer Support Sessions akin to those developed during the COVID-19 pandemic (33). The single item MBI assessment was essentially unchanged after compared to before the intervention, as were other indices of distress. The Authors underscore: “Despite increasing burnout in the specialty of emergency medicine…during the study time frame, burnout did not worsen in participants… [such that] formal peer support offered by EM groups can be an effective option to normalize the experience of being sued, promote wellness, and benefit physicians who endure the often long and stressful process of a medical malpractice lawsuit” (p. 205) (Table 4).

#### 3.6.4 Total OSI threat avoidant vigilance/symbolic aversiveness

Two of the questions in OSI Sub-section E on work conditions (exposure to visually-disturbing scenes and listening to emotionally-disturbing accounts) contribute to threat avoidant vigilance on the input level. The third threat avoidant vigilance input level element is the degree to which sustained alertness is required to avoid serious consequences. This element has a narrowed range (minimum of 1 for physicians without actual patient contact, such as is often the case for pathologists). The maximum score of 2 is given for clinicians with substantial emergency and/or inpatient work.

For all physicians, there is obviously potential injury or fatality that can be the consequence of a wrong decision. This is reflected on the central decision-making level of the OSI by a single threat avoidant vigilance stressor, which has a fixed score at the maximum (which is 2).

Hazardous task performance (output level) takes into account exposure to radiation, threat of violence and infection risk, also queried in OSI Sub-section E on work conditions. As seen in Supplementary material 3, altogether 84 of the 97 physicians included in the IPD stated that they faced risk of infection.

Sub-section F comes thereafter to inquire about more sensitive issues, termed “mishaps”, all of which contribute to threat avoidant vigilance, mainly on the general level. Among these are having witnessed or experienced accidents or injuries, litigation/testifying in court, including official complaints, suicide of patients and of colleagues or other persons at work, and lack of a system in place for non-medical emergencies. With the covariates (age, gender and working in direct contact with patients suspected to be infected with COVID-19 virus), total threat avoidant vigilance scores showed an OR (± 95% CI) = 1.25 (1.01–1.54) (*p* = 0.04) for Personal Burnout >46, and 1.31 (1.05–1.63) (*p* = 0.01) for Work-related Burnout >46 for 96 of the physicians in the IPD analysis.

### 3.7 Time pressure at work (OSI Sub-section G)

#### 3.7.1 Time constraints preclude completion of work tasks

Nearly half of the 97 physicians stated that sometimes or often it was not possible to complete their work tasks even with maximal effort. Time constraints precluding completion of work tasks were associated with a markedly higher likelihood of personal burnout, and a weaker relation to patient-related burnout in the IPD analysis (Table 3).

Workload/time pressure conflict has been associated directly or indirectly with physician burnout in some publications (Supplementary material 2). An observational study among hospital emergency professionals, including 34 physicians, underscored the heavy time pressure associated with that work environment (94). The need for multi-level solutions to address increased clinician overload/time pressure and burnout was highlighted in a study of 721 U.K. physicians, poignantly entitled: “It's like juggling fire daily” (95).

Although burnout *per se* was not reported, an intervention implemented among IM residents and faculty aimed to minimize conflicting responsibilities via a “clinic buddy” system (96). Time pressure was thereby reduced, and although continuity was also slightly diminished, there was no impact on patient outcomes. Of the many interventions implemented in the 5-year cohort study among IM and EM residents (30), more time was allowed for patient visits during clinic. Significantly improved empathy perception, sleep, peer support and nurture of personal relationships were reported during the follow-up period, as noted (Table 4).

### 3.8 Problems and restrictions (OSI Sub-section H)

#### 3.8.1 Problems/deficiencies hinder patient care—understaffing specifically hinders patient care

Over half of the 97 physicians indicated that problems/deficiencies occasionally or often hinder patient care. A greater frequency of such hindrances was associated with over three-fold likelihood of work-related burnout above 46 in the adjusted analysis. The most often cited hindrance was understaffing, reported by 44 of the 97 physicians. Albeit with a lower 95% CI of 0.86, when understaffing was indicated as a hindrance, the adjusted OR was nearly two for work-related burnout above the median of 46 (Table 3).

Among 1,925 European EM practitioners, 84% of whom were physicians, a survey carried out in early 2022 indicated an OR = 2.7 (95% CI 2.2–3.4) for abbreviated Maslach burnout associated with sometimes understaffing, and OR = 10.0 (95% CI 7.4–13.7) for often understaffing (97). Understaffing has been concordantly cited as a major stressor by physicians in various settings, who underscored the need for hiring more personnel (95, 98). In their open-ended responses, forty-eight (50%) of the 97 physicians included in the present IPD analysis suggested increased employment of staff as a needed work-place intervention (34, 39). It has been suggested that a “Chief Wellness Officer” could help alleviate these problems, e.g., by ensuring additional clinical staff teams during high demand seasons (99).

As summarized in Table 4, two studies implemented some type of increased clinician staffing. In (100), carried out in the primary care setting, a dyad of a physician and a certified medical assistant (CMA) was replaced in the intervention by a team of two health care providers and three CMAs who together were responsible for a panel of patients. At 6-month follow-up, emotional exhaustion and depersonalization were lowered among the physicians in the intervention group compared to baseline, and self-efficacy augmented, whereas a worsening trend was observed over this time period among the control group of physicians. In Quirk et al. (30), “jeopardy resident coverage” provided short-notice replacement for residents who needed time-off for vital personal life events. Together with the other interventions, improved outcomes were seen among the residents in that program. Interventions in which clerical staff are included to cover EHR-related tasks are reviewed in Section 3.10 on workload and activities.

#### 3.8.2 Interruptions from people hinder task performance

Thirty-one percent of the 97 physicians answered that interruptions from other people, including phone calls, occasionally prevented them from proceeding with their work. Another 17 physicians (18%) stated that such interruptions were frequent. The adjusted ORs for personal and work-related burnout above the median were, respectively, over 5 and nearly 8 in relation to these interruptions, which are the strongest multi-variable associations of all the examined work stressors (Table 3).

“Interruptions are [overall] considered one of the most common work stressors” (101) (p. 185), impinging on flow which is a key component of healthy work conditions (13, 14, 18, 31). Quite a few publications have emphasized the need to reduce interruptions for physicians (Supplementary material 2). Among 58 U.S. hospital physicians (102), frequent pages or interruptions for non-urgent matters showed the strongest bivariate relation of all the assessed job stressors to high MBI (*p* = 0.003).

Interventions to diminish physician interruptions in the outpatient setting suggested in the earlier-cited review article (13), included silencing phones when seeing patients, as well as during charting, and designating separate time to address non-urgent issues. A suggested intervention made by most of the 21 participating EM physicians (73) was to have “a separate space for themselves to reduce interruptions” (p. 270). Along these lines, as noted in Table 4, improved layout for radiology reading rooms appeared to help diminish interruptions, according to some of the physician statements as per (74). In particular, a deep reading space was designed for tasks that “require sustained periods of uninterrupted focus” (p. 111).

Implementing scheduling software for emergency surgery also reportedly reduced interruptions (103). “Geographical cohorting”, whereby physicians are limited to a single geographic location, was successfully carried out in the critical care setting (104), with several improved patient outcomes, as well as diminishing interruptions for the physicians. Residents together with the information technology (IT) staff developed an EHR-integrated text paging system which markedly diminished the number of interruptions, compared to traditional paging which required 100% telephone response. Although burnout was not explicitly assessed, “stress and frustration” were both reportedly reduced in 88% of the 25 participating residents (105). Only one publication was found assessing the impact of an intervention to reduce interruptions in actual relation to physician burnout (31). Among the 17 participating residents working in the emergency department, although the frequency of non-urgent calls decreased (*p* = 0.03), there was no change in reported degree of burnout from non-urgent calls (assessed via a single explicit query) (*p* = 0.84).

### 3.9 Interpersonal interactions and social climate (OSI Sub-section I)

#### 3.9.1 Lacking redress of grievances

Slightly fewer than half of the 97 physicians included in the IPD analysis stated that an efficient and confidential grievance procedure was available to them. Among the remaining physicians, either such a procedure, albeit available, was reportedly ineffective and/or without confidentiality guaranteed (42 physicians) or there was no possibility to redress grievances at work (seven physicians). In the adjusted logistic regression analysis, lack of an adequate procedure for redressing grievances was associated with over a two-fold likelihood of personal and work related burnout (Table 3).

Only one observational publication (106) was identified in which grievance redress was presented in relation to the MDs themselves. In three focus-groups, altogether 28 women physicians explored issues of gender inequity across career stages. Among the themes raised was the need for institutional transparency regarding grievances. Burnout *per se*, in specific relation to the lack of grievance procedures, did not appear to have been examined in that study.

None of the other queries in Section 3.9 of the OSI questionnaire concerning interpersonal issues showed any statistical relation to the burnout indices in the present IPD analysis. This may be due to the relatively favorable findings for the other queries in that Section, as seen in Supplementary material 3.

### 3.10 Workload and activities (OSI Sub-section J)

#### 3.10.1 Handling patients who cannot give a history

Only 25 of the 97 physicians stated that they rarely or never handled patients who could not give a history, while 22 stated that they frequently did so. This stressor was associated with over a two-fold increase in adjusted logistic regression analysis with all three types of burnout (Table 3). The burden of this stressor does not seem to have been included in other studies of physician burnout (Supplementary material 2).

#### 3.10.2 No separate time for non-clinical duties

Altogether 89 of the 97 physicians had other duties besides clinical work. Teaching in small groups and research were the most frequent of these, with administrative and other pedagogical tasks somewhat less common. Forty-six of these physicians had both pedagogical and research duties. Only 21 (24%) of the 89 physicians had separate time allocated for non-clinical assignments. Most were obliged to intersperse them with their clinical work. Adjusted multivariate logistic regression revealed over a three-fold elevated risk for personal and work-related burnout (>46) associated with lacking separate time allocated to non-clinical duties for those 89 physicians (Table 3).

A few studies have focused on the conflict between clinical and non-clinical duties, mainly among physicians in the academic setting for whom the need to separately allocate time for the multiple roles was emphasized (41). A publication from the Baskent University Ankara Hospital, Turkey, included 258 physicians for whom insufficient time for scientific research was associated with higher emotional exhaustion (*p* < 0.001) as assessed by the MBI (107). Concordantly, among 490 early-career “physician-scientists” in Japan, work-related burnout scores were lower among those with larger amounts of grant funding (*p* = 0.013), implying that more separate time could be allocated to scientific work (36).

An intervention study (108) of a cohort design was identified in which a faculty schedule change was instituted among eight acute care surgeons at a trauma and tertiary care center (Table 4). Protected academic time was one of the changes. In addition, continuous call was reduced from 24 to 12 h with no other clinical obligations during the on-call week. This ensured that the surgeon was free to leave as soon as the on-call was completed. Compared to baseline, at 1-year follow-up among the eight participating surgeons, overall MBI burnout was reduced by 12.5%, emotional exhaustion decreased by 28%, depersonalization was lowered by 38% and personal accomplishment increased by 12.5%. Clinical, administrative and academic parameters indicated that there was no diminution in the surgeons' productivity. Protected time for research was also one of the many interventions introduced among IM and EM residents, with improved empathy perception, sleep, peer support and nurture of personal relationships noted during the follow-up period (30).

#### 3.10.3 Performing tasks that seem pointless

Just over 20% of the 97 physicians answered that they were obliged to perform tasks they consider pointless. In the OSI model, this is a general level stressor within the conflict/uncertainty aspect, corresponding to lack of coherence. In adjusted analysis, the likelihood of personal burnout above the median was over three-fold higher, and of work-related burnout nearly five-fold higher among those 20 physicians (Table 3).

A closely-related concept is that of “illegitimate tasks” (109), that are not necessarily pointless, but are unreasonable and/or unfairly assigned. Such tasks may be within the domain of other personnel. For the present IPD, altogether 28 of the 97 physicians stated that they performed tasks outside the realm of a physician, i.e., duties of other personnel. Although there was an association between these two OSI queries (Pearson χ^2^ = 5.5, *p* = 0.02), only ten physicians answered that they performed seemingly pointless tasks as well as tasks of other personnel. There was no relation whatsoever between performing tasks of other personnel and any of the 3 burnout indices. These findings can be explained as follows: physicians may be called upon to perform tasks such as phlebotomy or recording electrocardiograms that are clinically essential, but should be performed by other personnel. For physicians, tasks that seem pointless are often of administrative/clerical (34). In the above-cited review (41), academic physicians reported that administrative responsibilities were the least meaningful. Moreover, spending <20% of work hours in the physician's perceived most meaningful activity reportedly showed the strongest association with burnout.

Many observational studies have addressed the burden on physicians of performing administrative tasks, especially regarding EHR, electronic health records (Supplementary material 2). Via the Berne Illegitimate Task scale (109), a strong relation (*p* < 0.001) was found between performing “illegitimate tasks” and personal, work-related and patient-related burnout among nearly 500 general practitioners in Germany (40).

An intervention to relieve administrative burden by providing clerical staff to cover some EHR tasks was carried out among seven academic internists in Birmingham, Alabama (110) (Table 4). At the 4-month follow-up, two physicians compared to four at baseline, reported feeling burned-out at least weekly from work, and none felt that they had become callous toward people (reflective of MBI depersonalization) compared to two of the seven who had felt so prior to the intervention. The Authors conclude that this intervention “allows physicians to spend more time focusing on patient care, resulting in improved patient interactions, increased productivity and improved physician satisfaction” (p. 363).

A year-long crossover study examined the impact of medical scribes among 18 primary-care physicians working at Kaiser Permanente in Northern California. The intervention was associated with diminished after-hours EHR documentation and greater chance of spending at least 75% of time interacting directly with the patient, rather than on computer. Although not explicitly assessed in their article, the Authors foresaw that reducing the EHR burden faced by primary care physicians could potentially diminish burnout (111). In the earlier described large-scale cross-sectional study (61) of urologists, explicitly carried out during the COVID-19 pandemic, hiring a scribe was, by self-report, considered the most effective strategy to reduce burnout. The Authors underscored the need for organizational support to “increase participation and effectiveness of burnout interventions” (p. 101).

#### 3.10.4 Total OSI conflict/uncertainty

The majority of the questions in sub-sections “Time pressure at work” (G) through “Workload and activities” (J) of the OSI questionnaire contribute to the Conflict/uncertainty aspect. The input level contains two elements that are scored maximally. Namely, signal/noise and signal/signal conflict are essential features for all physicians.

There is some variability regarding the degree to which there are conflicts/uncertainty at the central decision-making level. Handling patients who cannot give a history or who are severely disturbed, language barriers and delays/difficulties in obtaining medical records or lab are all potential sources of missing information needed for decision-making. The need to adjust plans due to unforeseen circumstances is scored higher with emergency and/or intensive care unit (ICU) responsibilities. However, one element of conflict/uncertainty on the central decision making level is scored maximally for all physicians, i.e., contradictory information.

Conflicting demands in time and space, to which time constraints contribute, problems/deficiencies that hinder patient care and interruptions are all elements of conflict/uncertainty on the output/task performance level. On the general level, conflict arises with the interpersonal issues addressed in Section 3.9 of the OSI questionnaire and some items in Section 3.10. As noted, lacking separate time for non-clinical duties and performing tasks that seem pointless were both associated in multivariable analysis with increased likelihood of personal and work-related burnout.

Of all the OSI aspects, the total conflict/uncertainty scores showed the most powerful associations with the three types of burnout, in the analyses that included covariates: age, gender and working in direct contact with patients suspected to be infected with COVID-19 virus. The OR (± 95% CI) was 1.45 (1.16–1.81) (*p* = 0.001) for personal burnout >46, 1.76 (1.32–2.34) (*p* = 0.0001) for work-related burnout >46 and 1.25 (1.003–1.55) (*p* = 0.04) for patient-related burnout >33 among the 96 physicians in the IPD analysis.

## 4 Discussion

The total stressor burden as assessed by the OSI showed a powerful multivariable association with all three burnout indices among the physicians included in the present IPD analysis. Working in direct contact with patients suspected to be infected with COVID-19 virus was yet an additional burden, whose impact appeared to be strongest for personal burnout. When the total OSI surpassed 88, the clinical cutpoint for urgent intervention, the likelihood of work-related burnout being above the median of 46, was over eight-fold. These findings for the total OSI justified the identification of potentially contributory job stressors, as performed herein for the IPD. Altogether there were 20 distinct work stressors showing multivariable association with one or more of the burnout indices.

The next step was to search for published interventions in which the implicated stressors were diminished among physicians. Altogether 33 publications were found. Burnout was explicitly assessed as an outcome in 21 (63.6%) investigations, with a favorable impact at least to the level of *p* < 0.10 for one or more indices observed in 13 (62%) of these studies. Two other intervention studies, each with fewer than ten physicians, showed a favorable, albeit statistically non-significant, impact on burnout (108, 110).

Six of the 33 intervention studies (37, 38, 50, 68, 100, 111) were of a randomized controlled design. In contrast, in 4 reports different physicians were examined prior to and after the intervention (53, 54, 56, 57). Two case studies (18, 48, 79) were included, as well. All but 5 of the 33 studies were explicitly conducted in the U.S.

For several of the implicated stressors, no published intervention studies to reduce the burden were found. These stressors were: insufficient work-free paid vacation, radiation exposure, suicide attempt or completed suicide of person(s) at work, lack of grievance procedure, and handling patients who cannot give a history. On the other hand, 8 of the 33 (24.2%) intervention studies addressed weekly work hours, mainly in relation to the 2003 mandated WHR in the U.S. for Post-Graduate Medical Education. All but one (55) of those reports showed an impact on burnout or other indices of physician wellbeing.

Of all the 33 identified intervention studies, the cluster-randomized investigation of 45 intensive care physicians at various levels of training (50) is deemed the most rigorous. Not only was the impact of diminishing the number of consecutive work days assessed in relation burnout and other indices of physician wellbeing, patient outcomes were also considered. Compared to standard continuous ICU responsibility for ~15 days, interrupted weekend cross-coverage yielded significantly diminished physician burnout without adversely affecting patient care.

Some other of the identified interventions (30, 96, 100) also implemented cross-coverage, effectively lowering time constraints (96). Emotional exhaustion was significantly reduced among the primary care physicians in the centers with cross-coverage, compared to those working in the status quo centers (100). In (30), cross-coverage provided short-notice replacement for urgent personal needs of the residents, as well as transferring after-hours paging to faculty. Cross-coverage among physicians was certainly also necessary to guarantee separate time for non-clinical activities. In the small intervention study of acute care surgeons carried out during the COVID-19 pandemic, favorable changes were observed in all three MBI indices a year after they were guaranteed protected academic time, as well as having assured coverage at the conclusion of their scheduled on-call duty (108).

Besides cross-coverage among physicians, staffing issues are also relevant for other team members in the health care system. In particular, administrative tasks often extend work hours. This impinges on the physician's scheduled free time, including vacations (63), as well as compromising attention to patients, and thereby undermining the meaningfulness of the physician's work (41). Assigning at least some administrative activity to clerical personnel, was beneficial for reducing burnout (61, 110), as well as diminishing after-hours worktime and allowing the physician to spend more time with patients (111). Collaboration between physicians and IT staff also appeared to be an effective strategy against interruptions (105). That stressor showed the most powerful multivariable associations with personal and work-related burnout for the physicians included in the IPD analysis. Partnership with IT staff could help develop other strategies to reduce the physician stressor load. Besides reducing administrative burden, measures could be instituted e.g., to safeguard against medication dosage errors through computer programming (18).

Rigorously-designed studies focused on a single stressor to which physicians are exposed are obviously vital for guiding evidence-based intervention strategies. However, such interventions may be insufficient to genuinely impact physician burnout, particularly if the overall stressor load is very heavy, e.g., with the total OSI score above the cutpoint of 88. Comprehensive organizational interventions were carried out among residents in (30). These would have lowered the scores for underload (recognition of good work), high demand (more days off plus after-workhour coverage) and conflict/uncertainty (diminished time constraints, improved staffing with cross-coverage, protected time for research). The total OSI would have been diminished by several points. In addition, there were interventions on the individual level: resiliency training, mental health support plus primary wellness care (30). Although the single-item explicit burnout measure did not change notably during the 5-years with implementation of these organizational and personal interventions, there was a significant increase in empathy perception, sleep, peer support and nurturing of personal relationships (30). Several other studies also reported interventions on the personal level (8, 18, 33, 38, 48, 60, 61, 78, 79, 82). Reduction in work hours together with counseling were associated with a nearly 30% diminution in full-time sick leave at 1-year follow-up for practicing physicians in Norway, as well as significantly lowered emotional exhaustion and depersonalization (60).

For physicians with full-blown burnout syndrome, or even more serious mental health disorders, return to work can be facilitated by care from a clinician with multifaceted expertise in psychiatry/psychology as well as occupational medicine (23). This was illustrated by the case study (18, 48) included herein. Multiple interventions were implemented on the organizational and individual level, coordinated by the occupational psychiatrist. With the immediate steps, some of which were temporary, the total OSI was lowered by 17 points [from 106 (in the “acute danger level”) to 89 (just above the cutpoint for urgent intervention needed)]. The physician, a psychiatrist herself, was thereby able to return to work, albeit, at first, in a limited capacity. With temporary release from emergency duty, threat avoidant vigilance was slightly diminished, as she was somewhat protected from visually disturbing scenes such as suicide and trauma, as well as from listening to the most intensely emotionally-disturbing accounts. The case study of the grieving anesthesiology resident faced with the death of the pediatric patient (79) concordantly underscores the vital importance of interventions that address the threat avoidant vigilance burden.

The heightened acute infection hazard associated with the COVID-19 pandemic intensified the threat avoidant vigilance burden for physicians. This, in turn, impacted other stressors. For example, the extra time needed to properly use personal protective equipment often compromised restbreaks, such that even when a hot cooked meal was provided free of charge, “adequate time to eat and digest such a meal was often lacking” (39) (p. 524). Cross-coverage by a “float” physician who is well informed about the clinical status of the patients for whom he/she is covering could have been implemented to ensure adequate rest breaks for colleagues (34). Unfortunately, none of the larger-scale intervention studies addressing this topic (38, 58, 68) were conducted during the COVID-19 pandemic. Moreover, studies (38, 58, 68) do not appear to have been designed to assess the impact of providing properly-covered restbreaks during active work hours for physicians. Such investigations are urgently needed, given the potential benefits of this intervention (112).

Quirk et al. (30) provide an example of the potential feasibility of implementing a “package” of organizational interventions among a fairly sizable group of resident physicians. Included therein are some initial approaches to providing cross coverage. The latter is an essential step for many of the needed interventions, including ensuring adequate rest breaks. A “float” physician is an excellent solution, which, during on-site evaluation, this author has observed to be very practical. An initial outlay may be needed to cover this extra staffing, which can be a barrier. In addition, close cooperation among the physicians is vital, and this entails effort and training. In the longer run, this strategy would be cost-effective in protecting the health and work capacity of the physicians. The role of “float” physician can be very rewarding, bolstering knowledge and skills. The potential social cohesion engendered thereby is an added benefit.

The emotional toll of physicians' work during the COVID-19 pandemic further contributed to the threat avoidant vigilance burden. Although burnout, *per se*, was not assessed in Monette et al. (78), the weekly debriefings for clinicians providing emergency services during that time, were seen to facilitate a “safe environment”. Further helpful countermeasures regarding personal accomplishments were seen in the peer-to-peer recognition program for EM residents carried out during 2021–2022 (8).

Recognition of the special service to the community was implemented through discounts at local shops for the health care providers working at the COVID-19 Outpatient Respiratory Center in Nedić and Belkić (39). As noted, nearly all the median scores on Section 3.9 Interpersonal interactions and social climate were favorable with narrow interquartile ranges for the physicians included in the IPD analysis. Thus, intervention studies regarding these topics were not assessed in the present review. On the other hand, the median night shift work scores were maximal, such that limited variance did not permit detection of the impact of this stressor on physician burnout.

Further, regarding the IPD, all the data were cross-sectional and were mainly self-reported. This precludes inferences about the temporal nature of the identified associations and common method bias cannot be excluded. Based on these findings from cross-sectional data, the relationships between exposure to specific stressors and burnout among the physicians included in the IPD analysis cannot be unequivocally viewed as causal. Longitudinal follow-up studies are needed to help to establish causality. Interventional designs, larger scale as well as clinical case studies, can provide convergent validation, insofar as diminished exposure to one or more specific stressors is associated with amelioration of burnout.

There was a limited number of fairly small studies fulfilling all the inclusion criteria for the IPD. Consequently, power limitations must be considered. These also reduce the generalizability of the IPD findings. The relatively small percentage of senior level, attending physicians further diminishes generalizability.

The IPD data were collected from physicians working at an Academic Medical Institute in South Asia (India) and an ambulatory medical center in SouthEast Europe (Serbia). While potentially enhancing the generalizability of the IPD findings, these geographic and institutional factors require attention.

The identified intervention studies were from the U.S. and Western Europe. Since physician burnout is a global concern, a much broader international perspective is needed. Particular attention is warranted regarding the cultural setting, with greater appreciation of the needs of physicians working in developing countries.

Notwithstanding the limitations of the present work, the individual participant data analysis by the MD-specific Occupational Stressor Index provides substantial clues as to how physician burnout could be better prevented. Several of the identified contributory stressors had been previously underappreciated. The systematic review of intervention studies on the OSI-identified stressors further helps fill the gap, by focusing on the called-for attention to reduction of specific stressors. Together, these two facets of the present paper help pave the way toward more effective strategies to combat physician burnout, contributing to the following conclusions.

## 5 Conclusions

The strength and consistency of the reported evidence support the implementation of cross-coverage, so that physicians are guaranteed, at the very least, one day per week or alternating weekends entirely free from work obligations. Concordantly, an upper limit to weekly work hours including constraints on intrusions into free-time outside work hours is recommended. Work-free, paid vacation of adequate duration as well as appropriately timed and cross-covered rest breaks need to be examined as interventions to protect against physician burnout. The stressors related to work hours and scheduling contribute to high demands among physicians and are associated with increased personal and work-related burnout.

Recognition of the physician's efforts and achievements is a vital and easily implementable intervention. This would counteract underload, thereby diminishing work-related and patient-related burnout.

Radiation exposure and prolonged time spent in inadequate office/workspace adversely impact physician burnout. With the heightened infection risk during the COVID-19 pandemic, untoward physical conditions increased the hazards of physicians' work. An overly heavy exposure to that which is emotionally-disturbing further contributes to the burden. Suicide, whether of a patient, colleague or other coworker, is particularly devastating, as are medical errors that eventuate in lawsuits or other official complaints, for which the physician is often the “second victim”. Supportive counter-measures are indispensable. All these exposures add to the threat avoidant vigilance load, increasing the risk of personal and work-related burnout.

Although conflicts and uncertainty are part and parcel of physicians' work, modifiable stressors exacerbate this burden. Adequate staffing would be pivotal for mitigating many of these. Clerical staff can help offload administrative burden, allowing physicians to focus attention more directly on patients. Information technology staff can aid in devising ways to diminish interruptions, thereby enhancing work flow. Cross coverage among physicians can lower time constraints and ensure separate periods for vital non-clinical tasks. The total conflict/uncertainty score, assessed via the physician-specific OSI impacts heavily on all three types of burnout, as assessed by the CBI.

The total OSI score is a powerful gauge of personal, work-related and patient-related burnout risk among physicians, even when accounting for work directly with patients suspected of acute infection with COVID-19 virus. In clinical practice, lowering the total OSI score has facilitated return to healthier work for physicians suffering from burnout. The MD-specific OSI, developed “for physicians, by physicians” and based upon cognitive ergonomics is useful for guiding participatory action research. Well-controlled trials, with appropriately tailored interventions (77) would thereby help alleviate the scourge of burnout among this profession.

## Data Availability

The original contributions presented in the study are included in the article/Supplementary material, further inquiries can be directed to the corresponding author.
