# Supplementary material for: Toward better prevention of physician burnout: insights from individual participant data using the MD-specific Occupational Stressor Index and organizational interventions
Source: Front Public Health. 2025 Mar 19;13:1514706. doi: 10.3389/fpubh.2025.1514706 (PMC11961930; doi:10.3389/fpubh.2025.1514706)
Supplement: Supplementary file 1 [file Data_Sheet_1.docx]

**Supplement 1: Identification of Potential Studies for the Individual Participant Data Analysis**

**Search Branch 1: Studies on Physician Burnout applying the Copenhagen Burnout Inventory**

Search strategy: (physicians or doctors) AND (Copenhagen Burnout Inventory or CBI) with work stressors assessed

1.Abdalla RN, Ansari SA, Hurley MC, Attarian H, Fargen KM, Hirsch JA, et al. [Correlation of call burden and sleep deprivation with physician burnout, driving crashes, and medical errors among US neurointerventionalists.](https://pubmed-ncbi-nlm-nih-gov.proxy.kib.ki.se/36007952/) AJNR Am J Neuroradiol. 2022;43(9):1286-91. doi: 10.3174/ajnr.A7606.

----------------------

2.Aljabri D, Alshatti F, Alumran A, Al-Rayes S, Alsalman D, Althumairi A, et al. Sociodemographic and occupational factors associated with burnout: a study among frontline healthcare workers during the COVID-19 pandemic. Front. Public Health. 2022; 10: 54687. doi: 10.3389/fpubh.2022.854687

----------------------

3.Alsulimani LK, Farhat AM, Borah RA, AlKhalifah JA, Alyaseen SM, Alghamdi SM, et al. Health care worker burnout during the COVID-19 pandemic: A cross-sectional survey study in Saudi Arabia. Saudi Med J. 2021; 42(3):306-14.

----------------------

4.Ashraf F, Ahmad H, Aftab S. [Interactive role of personal and work related factors in psychological burnout: A study of Pakistani doctors.](https://pubmed-ncbi-nlm-nih-gov.proxy.kib.ki.se/32794497/) J Pak Med Assoc. 2020;70(8):1413-7.

----------------------

5.Bagaajav A, Myagmarjav S, Nanjid K, Otgon S, Chae YM. Burnout and job stress among Mongolian doctors and nurses. Ind Health. 2011;49(5):582-8. doi: 10.2486/indhealth.ms1256

----------------------

6.Baptista S, Teixeira A, Castro L, Cunha M, Serrão C, Rodrigues A, et al. Physician burnout in primary care during the COVID-19 Pandemic: A cross-sectional study in Portugal. J Prim Care Community Health. 2021;12:1-9 doi: 10.1177/2150132721100843.

------------------------------

7. Belkić K, Rustagi N. Job stressors in relation to burnout and compromised sleep among academic physicians in India. Work. 2024; 78: 505–525.

------------------------------

8. Benson S, Sammour T, Neuhaus SJ, Findlay B, Hill AG. [Burnout in Australasian younger fellows.](https://pubmed-ncbi-nlm-nih-gov.proxy.kib.ki.se/19895513/) ANZ J Surg. 2009;79(9):590-7. doi: 10.1111/j.1445-2197.2009.05012.x.

------------------------------

9. Berger B, Cungi PJ, Arzalier S, Lieutaud T, Velly L, Simeone P, et al. [Incidence of burnout syndrome among anesthesiologists and intensivists in France: The REPAR Study.](https://pubmed-ncbi-nlm-nih-gov.proxy.kib.ki.se/36767139/) Int J Environ Res Public Health. 2023;20(3):1771

------------------------------

10. Bhembe LQ, Tsai FJ. [Occupational stress and burnout among health care workers caring for people living with HIV in Eswatini.](https://pubmed-ncbi-nlm-nih-gov.proxy.kib.ki.se/30865060/) J Assoc Nurses AIDS Care. 2019;30(6):639-47. doi: 10.1097/JNC.0000000000000068

------------------------------

11. Biksegn A, Kenfe T, Matiwos S, Eshetu G. [Burnout status at work among health care professionals in a tertiary hospital.](https://pubmed-ncbi-nlm-nih-gov.proxy.kib.ki.se/27222622/) Ethiop J Health Sci. 2016;26(2):101-8. doi: 10.4314/ejhs.v26i2.3.

------------------------------

12. Bodendieck E, Jung FU, Luppa M, Riedel-Heller SG. [Burnout and work-privacy conflict - are there differences between full-time and part-time physicians?](https://pubmed-ncbi-nlm-nih-gov.proxy.kib.ki.se/36002851/) BMC Health Serv Res. 2022;22(1):1082. doi: 10.1186/s12913-022-08471-8.

------------------------------

13. Boegelund Kristensen T, Kelstrup Hallas M, Høgsted R, Groenvold M, Sjøgren P, et al. [Burnout in physicians: A survey of the Danish society for palliative medicine.](https://pubmed-ncbi-nlm-nih-gov.proxy.kib.ki.se/34187876/) Support Palliat Care. 2021:bmjspcare-2021-003237. doi: 10.1136/bmjspcare-2021-003237.

------------------------------

14.Borritz M, Rugulies R, Bjorner JB, Villadsen E, Mikkelsen OA, Kristensen TS. [Burnout among employees in human service work: design and baseline findings of the PUMA study.](https://pubmed-ncbi-nlm-nih-gov.proxy.kib.ki.se/16449044/) Scand J Public Health. 2006;34(1):49-58. doi: 10.1080/14034940510032275.

------------------------------

15.Caesar B, Barakat A, Bernard C, Butler D.  [Evaluation of physician burnout at a major trauma centre using the Copenhagen burnout inventory: cross-sectional observational study.](https://pubmed-ncbi-nlm-nih-gov.proxy.kib.ki.se/32285375/) Ir J Med Sci. 2020;189(4):1451-6. doi: 10.1007/s11845-020-02223-5.

------------------------------

16.Chambers CN, Frampton CM, Barclay M, McKee M. [Burnout prevalence in New Zealand's public hospital senior medical workforce: A cross-sectional mixed methods study.](https://pubmed-ncbi-nlm-nih-gov.proxy.kib.ki.se/27881531/) BMJ Open. 2016; 6(11):e013947. doi: 10.1136/bmjopen-2016-013947.

--------------------------

17.Chou LP, Li CY, Hu SC. [Job stress and burnout in hospital employees: comparisons of different medical professions in a regional hospital in Taiwan.](https://pubmed-ncbi-nlm-nih-gov.proxy.kib.ki.se/24568961/) BMJ Open. 2014;4(2):e004185. doi: 10.1136/bmjopen-2013-004185.

--------------------------

18.Chu WM, Ho HE, Lin YL, Li JY, Lin CF, Chen CH, et al.  [Risk factors surrounding an increase in burnout and depression among health care professionals in Taiwan during the COVID-19 pandemic.](https://pubmed-ncbi-nlm-nih-gov.proxy.kib.ki.se/36592937/) J Am Med Dir Assoc. 2023;24(2):164-70.e3. doi: 10.1016/j.jamda.2022.12.010.

--------------------------

19.Correia C, Teixeira R, de Almeida NMP, Morais S, Figueiredo P. [Burnout in gastrenterologists: a national-level analysis.](https://pubmed-ncbi-nlm-nih-gov.proxy.kib.ki.se/34403305/) Scand J Gastroenterol. 2021;56(11):1271-8. doi: 10.1080/00365521.2021.1961308.

--------------------------

20.Dev V, Fernando AT 3rd, Consedine NS. [Self-compassion as a stress moderator: A cross-sectional study of 1700 doctors, nurses, and medical students.](https://pubmed-ncbi-nlm-nih-gov.proxy.kib.ki.se/32435318/) Mindfulness (N Y). 2020;11(5):1170-81. doi: 10.1007/s12671-020-01325-6.

--------------------------

21.Doppia MA, Estryn-Béhar M, Fry C, Guetarni K, Lieutaud T; comité de pilotage de l'enquête SESMAT. [[Burnout in French doctors: a comparative study among anaesthesiologists and other specialists in French hospitals (SESMAT study)].](https://pubmed-ncbi-nlm-nih-gov.proxy.kib.ki.se/21981850/) Ann Fr Anesth Reanim. 2011;30(11):782-94. French doi: 10.1016/j.annfar.2011.05.011.

--------------------------

22.Estephan L, Pu C, Bermudez S, Waits A. Burnout, mental health, physical symptoms, and coping behaviors in healthcare workers in Belize amidst COVID-19 pandemic: A nationwide cross-sectional study.Int J Soc Psychiatry. 2023;69(4):1033-1042. doi: 10.1177/00207640231152209

--------------------------

23.Fernando BMS, Samaranayake DL.[Burnout among postgraduate doctors in Colombo: Prevalence, associated factors and association with self-reported patient care.](https://pubmed-ncbi-nlm-nih-gov.proxy.kib.ki.se/31619216/) BMC Med Educ. 2019;19(1):373. doi: 10.1186/s12909-019-1810-9.

--------------------------

24.Ferry AV, Wereski R, Strachan FE, Mills NL. Predictors of UK healthcare worker burnout during the COVID-19 pandemic. QJM. 2021; 114(6): 374–80.

--------------------------

25.Fiabane E, Margheritti S, Aiello EN, Magnone S, Miglioretti M, Gabanelli P, et al. [Prevalence and determinants of Italian physicians' burnout in the "post-COVID-19" era.](https://pubmed-ncbi-nlm-nih-gov.proxy.kib.ki.se/36335513/) Int Arch Occup Environ Health. 2023;96(3):377-87. doi: 10.1007/s00420-022-01929-6

--------------------------

26.Frajerman A, Colle R, Hozer F, Deflesselle E, Rotenberg S, Chappell K, et al. [Psychological distress among outpatient physicians in private practice linked to COVID-19 and related mental health during the second lockdown.](https://pubmed-ncbi-nlm-nih-gov.proxy.kib.ki.se/35447507/) J Psychiatr Res. 2022;151:50-6. doi: 10.1016/j.jpsychires.2022.04.003.

--------------------------

27.Frajerman A, Deflesselle E, Colle R, Corruble E, Costemale-Lacoste JF. [[Burnout, anxiety, insomnia and depressive symptoms among French outpatient physicians in the second wave of COVID-19: Comparison between general practitioners and specialists].](https://pubmed-ncbi-nlm-nih-gov.proxy.kib.ki.se/37121809/) Encephale. 2023:S0013-7006(23)00043-X. French

--------------------------

28.Fteropoulli T, Kalavana T, Yiallourou A, Karaiskakis M, Koliou Mazeri M, Vryonides S, et al. Beyond the physical risk: Psychosocial impact and coping in healthcare professionals during the COVID-19 pandemic. J Clin Nurs. 2021; doi:10.111jocn.15938 Online ahead of print

------------------------

29. Funding E, Viftrup DT, Knudsen MB, Haunstrup LM, Tolver A, Clemmensen SN. [Impact of training in serious illness communication and work life balance on physicians' self-efficacy, clinical practice and perception of roles.](https://pubmed-ncbi-nlm-nih-gov.proxy.kib.ki.se/37283658/) Adv Med Educ Pract. 2023;14:547-55. doi: 10.2147/AMEP.S406570.

------------------------

30.Gemine R, Davies GR, Tarrant S, Davies RM, James M, Lewis K. Factors associated with work‐related burnout in NHS staff during COVID‐19: a cross‐sectional mixed methods study. BMJ Open. 2021;11(1):e042591.

-----------------------------

31.Gonçalves JV, Castro L, Nunes R, Rêgo G. [Burnout among physicians working in palliative care during the COVID-19 Pandemic in Portugal: A cross-sectional study.](https://pubmed-ncbi-nlm-nih-gov.proxy.kib.ki.se/36898212/) Acta Med Port. 2023 Mar 1;36(3):183-92. doi: 10.20344/amp.18361.

-----------------------------

32.Goyal P, Rustagi N, Belkić K. Physicians’ total burden of occupational stressors: Over three-fold increased odds of burnout. South Med J. 2021; 114(7): 409-415.

-----------------------------

33.Haffizulla F, Newman C, Kaushai S, Williams C, Haffizulla A, Hardigan P, et al. Assessment of burnout: A pilot study of international women physicians. Perm J. 2020; 24:20.028. doi.org/10.7812/TPP/20.028

-----------------------------

34.Hajebi A, Abbasinejad M, Zafar M, Hajebi A, Taremian F.  [Mental Health, burnout, and job stressors among healthcare workers during the COVID-19 pandemic in Iran: A cross-sectional survey.](https://pubmed-ncbi-nlm-nih-gov.proxy.kib.ki.se/35633777/) Front Psychiatry. 2022;13:891430. doi: 10.3389/fpsyt.2022.891430

-----------------------------

35.Hardy P, Costemale-Lacoste JF, Trichard C, Butlen-Ducuing F, Devouge I, Cerboneschi V, et al. Comparison of burnout, anxiety and depressive syndromes in hospital psychiatrists and other physicians: Results from the ESTEM study. Psychiatry Res. 2020;284:112662. doi: 10.1016/j.psychres.2019.112662.

-----------------------------

36.Harkin D, Alzayyat S, Kiernan A, Ryan Á, Boland F, Renton S, et al. [Vascular surgeon burnout and resilience in the United Kingdom: A Report from the Vascular Society Workforce Committee.](https://pubmed-ncbi-nlm-nih-gov.proxy.kib.ki.se/35248742/) Ann Vasc Surg. 2022;84:169-78. doi: 10.1016/j.avsg.2022.01.032.

-----------------------------

37.Heinke W, Dunkel P, Brähler E, Nübling M, Riedel-Heller S, Kaisers UX. [Burnout in anesthesiology and intensive care : is there a problem in Germany?](https://pubmed-ncbi-nlm-nih-gov.proxy.kib.ki.se/22071874/) Anaesthesist. 2011;60(12):1109-18. [German] doi: 10.1007/s00101-011-1947-3

-----------------------------

38.Hussain M, Amjad MB, Ahsan J, Minhas SO. Implementation of National Institute of Health guidelines and other factors contributing to work-related burnout in COVID isolation ward and ICU physicians. J Ayub Med Coll Abbottabad 2021;33(2):283–8.

-----------------------------

39.Ilić IM, Arandjelović MŽ, Jovanović JM, Nešić MM. [Relationships of work-related psychosocial risks, stress, individual factors and burnout - Questionnaire survey among emergency physicians and nurses.](https://pubmed-ncbi-nlm-nih-gov.proxy.kib.ki.se/28345677/) Med Pr. 2017 Mar 24;68(2):167-78. doi: 10.13075/mp.5893.00516.

------------------------

40.Ireland MJ, Clough B, Gill K, Langan F, O'Connor A, Spencer L. [A randomized controlled trial of mindfulness to reduce stress and burnout among intern medical practitioners.](https://pubmed-ncbi-nlm-nih-gov.proxy.kib.ki.se/28379084/) Med Teach. 2017;39(4):409-414.

------------------------

41.Jung FU, Bodendieck E, Bleckwenn M, Hussenoeder FS, Luppa M, Riedel-Heller SG. [Burnout, work engagement and work hours - how physicians' decision to work less is associated with work-related factors.](https://pubmed-ncbi-nlm-nih-gov.proxy.kib.ki.se/36793035/) BMC Health Serv Res. 2023;23(1):157. doi: 10.1186/s12913-023-09161-9.

------------------------

42.Kassam A, Horton J, Shoimer I, Patten S. [Predictors of well-being in resident physicians: A descriptive and psychometric study.](https://pubmed-ncbi-nlm-nih-gov.proxy.kib.ki.se/26217426/) J Grad Med Educ. 2015;7(1):70-4. doi: 10.4300/JGME-D-14-00022.1.

------------------------

43.Klein J, Grosse Frie K, Blum K, von dem Knesebeck O. [Burnout and perceived quality of care among German clinicians in surgery.](https://pubmed-ncbi-nlm-nih-gov.proxy.kib.ki.se/20935011/) Int J Qual Health Care. 2010;22(6):525-30. doi: 10.1093/intqhc/mzq056.

------------------------

44.Kurzthaler I, Kemmler G, Holzner B, Hofer A. [Physician's burnout and the COVID-19 Pandemic-A nationwide cross-sectional study in Austria.](https://pubmed-ncbi-nlm-nih-gov.proxy.kib.ki.se/34950073/) Front Psychiatry. 2021;12:784131.

-----------------------------

45.La Torre G, Sestili C, Imeshtari V, Masciullo C, Rizzo F, Guida G, et al. [Association of health status, sociodemographic factors and burnout in healthcare professionals: results from a multicentre observational study in Italy.](https://pubmed-ncbi-nlm-nih-gov.proxy.kib.ki.se/34029976/) Public Health. 2021;195:15-7. doi: 10.1016/j.puhe.2021.04.004.

-----------------------------

46.Lapa T, Carvalho S, Viana J, Ferreira PL, Pinto-Gouveia J, Cabete AB. [Development and evaluation of a Global Burnout Index derived from the use of the Copenhagen Burnout Inventory in Portuguese physicians.](https://pubmed-ncbi-nlm-nih-gov.proxy.kib.ki.se/30387421/) Acta Med Port. 2018;31(10):534-41. doi: 10.20344/amp.10407.

-----------------------------

47.Lazari S, Zaitoon H, Shofaniyeh I, Sheikh-Ahmad M, Agbarya A.  [Bnai-Zion Medical Center healthcare workers' levels of professional burnout during the COVID-19 pandemic.](https://pubmed-ncbi-nlm-nih-gov.proxy.kib.ki.se/37226338/) Am J Health Behav. 2023;47(2):397-407. doi: 10.5993/AJHB.47.2.19

-----------------------------

48.Lue BH, Chen HJ, Wang CW, Cheng Y, Chen MC.  [Stress, personal characteristics and burnout among first postgraduate year residents: A nationwide study in Taiwan.](https://pubmed-ncbi-nlm-nih-gov.proxy.kib.ki.se/20423259/) Med Teach. 2010;32(5):400-7. doi: 10.3109/01421590903437188.

-----------------------------

49.Majeed F, Liaqat N, Hussain MM, Iqbal A, Hashim I, Saleem M. [Burnout among postgraduate residents](https://pubmed-ncbi-nlm-nih-gov.proxy.kib.ki.se/36377157/)  using Copenhagen Burnout Inventory. J Ayub Med Coll Abbottabad. 2022;34(3):463-7. doi: 10.55519/JAMC-03-9594.

-----------------------------

50.Mari S, Meyen R, Kim B. [Resident-led organizational initiatives to reduce burnout and improve wellness.](https://pubmed-ncbi-nlm-nih-gov.proxy.kib.ki.se/31775717/) BMC Med Educ. 2019 Nov 27;19(1):437. doi: 10.1186/s12909-019-1756-y. doi: 10.1186/s12909-019-1756-y.

-----------------------------

51.McEntee K, Koenig H, Hattigangadi R, Loring M, Brockmeyer A, Dahlman M. Factors associated with burnout among minimally invasive gynecologic surgery fellows. AJOG Glob Rep. 2022; 2(3): 100074. doi.org/10.1016/j.xagr.2022.100074

-----------------------------

52.McNicholas F, Sharma S, Oconnor C, Barrett E. [Burnout in consultants in child and adolescent mental health services (CAMHS) in Ireland: A cross-sectional study.](https://pubmed-ncbi-nlm-nih-gov.proxy.kib.ki.se/31959602/) BMJ Open. 2020;10(1):e030354. doi: 10.1136/bmjopen-2019-030354.

-----------------------------

53.McNicholas F, Adamis D, Minihan E, Doody N, Gavin B. [Occupational stress in clinical and non-clinical staff in Child and Adolescent Mental Health Services (CAMHS): A cross-sectional study.](https://pubmed-ncbi-nlm-nih-gov.proxy.kib.ki.se/35403597/) Ir J Psychol Med. 2022:1-7. doi: 10.1017/ipm.2022.

-----------------------------

54.Messias E, Gathright MM, Freeman ES, Flynn V, Atkinson T, Thrush CR, et al. [Differences in burnout prevalence between clinical professionals and biomedical scientists in an academic medical centre: a cross-sectional survey.](https://pubmed-ncbi-nlm-nih-gov.proxy.kib.ki.se/30782882/) BMJ Open. 2019; 9(2):e023506. doi: 10.1136/bmjopen-2018-023506.

-----------------------------

55.Mijić Marić A, Palameta M, Zalihić A, Bender M, Mabić M, Berberović M, et al. Prevalence of burnout among health care workers in the Federation of Bosnia and Herzegovina during the coronavirus disease-2019 pandemic: A cross-sectional study.Croat Med J. 2022; 63(5):482-9. doi: 10.3325/cmj.2022.63.482.

-------------------

56.Nedić O, Belkić K. Job stressors and burnout among nurses and primary-care physicians working at a dedicated outpatient respiratory center for patients with suspected or confirmed COVID-19. Am J Ind Med. 2023; 66 (6): 510-28.

-----------------------------

57.Nimer A, Naser S, Sultan N, Alasad RS, Rabadi A, Abu-Jubba M, et al. [Burnout syndrome during residency training in Jordan: prevalence, risk factors, and implications.](https://pubmed-ncbi-nlm-nih-gov.proxy.kib.ki.se/33562100/) Int J Environ Res Public Health. 2021 Feb 6;18(4):1557. doi: 10.3390/ijerph18041557.

-----------------------------

58.Ovalle Diaz J, Gorgen ARH, Teixeira da Silva AG, de Oliveira Paludo A, Timóteo de Oliveira R, Rosito N, et al. [Burnout syndrome in pediatric urology: A perspective during the COVID-19 pandemic - Ibero-American survey.](https://pubmed-ncbi-nlm-nih-gov.proxy.kib.ki.se/33602610/) J Pediatr Urol. 2021;17(3):402.e1-402.e7. doi: 10.1016/j.jpurol.2021.01.015.

-----------------------------

59.Parola V, Coelho A, Cardoso D, Sandgren A, Apóstolo J. [Prevalence of burnout in health professionals working in palliative care: A systematic review.](https://pubmed-ncbi-nlm-nih-gov.proxy.kib.ki.se/28708752/) JBI Database System Rev Implement Rep. 2017;15(7):1905-33. doi: 10.11124/JBISRIR-2016-003309.)

-----------------------------

60.Perumalswami CR, Takenoshita S, Tanabe A, Kanda R, Hiraike H, Okinaga H, et al. Workplace resources, mentorship, and burnout in early career physician-scientists: a cross sectional study in Japan. BMC Med Educ. 2020;20(1):178. doi: 10.1186/s12909-020-02072-x.

-----------------------------

61.Pius RE, Ajuluchukwu JN, Roberts AA. [Prevalence and correlates of burn-out among Nigerian medical doctors during the COVID-19 pandemic: a cross-sectional study.](https://pubmed-ncbi-nlm-nih-gov.proxy.kib.ki.se/37996233/) BMJ Open. 2023;13(11):e076673. doi: 10.1136/bmjopen-2023-076673.

-----------------------------

62.Portier G, Mathonnet M. [The workplace quality of life of university hospital digestive surgeons: Results of a 2019 nationwide survey.](https://pubmed-ncbi-nlm-nih-gov.proxy.kib.ki.se/34016570/) J Visc Surg. 2022;159(3):201-5. doi: 10.1016/j.jviscsurg.2021.03.010.

-----------------------------

63.Ratnakaran B, Prabhakaran A, Karunakaran V. [Prevalence of burnout and its correlates among residents in a tertiary medical center in Kerala, India: A cross-sectional study.](https://pubmed-ncbi-nlm-nih-gov.proxy.kib.ki.se/27320952/) J Postgrad Med. 2016;62(3):157-61. doi: 10.4103/0022-3859.184274

-----------------------------

64.Rogers E, Polonijo AN, Carpiano RM. [Getting by with a little help from friends and colleagues: Testing how residents' social support networks affect loneliness and burnout.](https://pubmed-ncbi-nlm-nih-gov.proxy.kib.ki.se/28661887/) Can Fam Physician. 2016;62(11):e677-e683

-----------------------------

65.Sato TO, de Faria BSF, Albuquerque BB, Silva FLD, Rohwedder LS, de Azevedo RT, et al. [Poor health conditions among Brazilian Healthcare Workers: The study design and baseline characteristics of the HEROES Cohort.](https://pubmed-ncbi-nlm-nih-gov.proxy.kib.ki.se/36292544/) Healthcare (Basel). 2022;10(10):2096. doi: 10.3390/healthcare10102096.

-----------------------------

66.Scheepers R, Silkens M, van den Berg J, Lombarts K. [Associations between job demands, job resources and patient-related burnout among physicians: results from a multicentre observational study.](https://pubmed-ncbi-nlm-nih-gov.proxy.kib.ki.se/32973063/) BMJ Open. 2020;10(9):e038466. doi: 10.1136/bmjopen-2020-038466.

-----------------------------

67.Sturm H, Rieger MA, Martus P, Ueding E, Wagner A, Holderried M, et al. [Do perceived working conditions and patient safety culture correlate with objective workload and patient outcomes: A cross-sectional explorative study from a German university hospital.](https://pubmed-ncbi-nlm-nih-gov.proxy.kib.ki.se/30608945/) PLoS One. 2019;14(1):e0209487. doi: 10.1371/journal.pone.0209487.

-----------------------------

68.Theodorou P, Georgantoni M, Maria-Elissavet P, Charalampos P, Thalia B. [Job Satisfaction and burnout levels of the human resources of a public oncology hospital during the COVID-19 pandemic.](https://pubmed-ncbi-nlm-nih-gov.proxy.kib.ki.se/37581808/) Adv Exp Med Biol. 2023;1425:345-52. doi: 10.1007/978-3-031-31986-0_33

-----------------------------

69.Thrush CR, Gathright MM, Atkinson T, Messias EL, Guise JB. [Psychometric properties of the Copenhagen Burnout Inventory in an academic healthcare institution sample in the U.S.](https://pubmed-ncbi-nlm-nih-gov.proxy.kib.ki.se/32539552/) Eval Health Prof. 2021;44(4):400-5. doi: 10.1177/0163278720934165

-----------------------------

70.Toyoshima M, Takenoshita S, Hasegawa H, Kimura T, Nomura K. E[xperiences of negotiations for improving research environment and burnout among young physician researchers in Japan.](https://pubmed-ncbi-nlm-nih-gov.proxy.kib.ki.se/32698340/) Int J Environ Res Public Health. 2020;17(14):5221. doi: 10.3390/ijerph17145221.

-----------------------------

71.Tsai YL, Tung YC, Cheng Y. [Surveys of burnout among physicians in Taiwan.](https://pubmed-ncbi-nlm-nih-gov.proxy.kib.ki.se/32995210/) J Acute Med. 2018;8(3):86-98. doi: 10.6705/j.jacme.201809_8(3).0002.

-----------------------------

72.Wagner A, Rieger MA, Manser T, Sturm H, Hardt J, Martus P, et al. [Healthcare professionals' perspectives on working conditions, leadership, and safety climate: a cross-sectional study.](https://pubmed-ncbi-nlm-nih-gov.proxy.kib.ki.se/30665401/) BMC Health Serv Res. 2019;19(1):53 doi: 10.1186/s12913-018-3862-7.

-----------------------------

73.Walker AL, Gamble J, Creedy DK, Ellwood DA.  [Impact of traumatic birth on Australian obstetricians: A pilot feasibility study.](https://pubmed-ncbi-nlm-nih-gov.proxy.kib.ki.se/31828770/) Aust N Z J Obstet Gynaecol. 2020;60(4):555-60. doi: 10.1111/ajo.13107.

-----------------------------

74.Werdecker L, Esch T. [Burnout, satisfaction and happiness among German general practitioners (GPs): A cross-sectional survey on health resources and stressors.](https://pubmed-ncbi-nlm-nih-gov.proxy.kib.ki.se/34143849/) PLoS One. 2021;18;16(6):e0253447. doi: 10.1371/journal.pone.0253447

-----------------------------

75.Wright JG, Khetani N, Stephens D. [Burnout among faculty physicians in an academic health science centre.](https://pubmed-ncbi-nlm-nih-gov.proxy.kib.ki.se/22851895/) Paediatr Child Health. 2011;16(7):409-13. doi: 10.1093/pch/16.7.409

-----------------------------

76.Youssef D, Abboud E, Abou-Abbas L, Hassan H, Youssef J. [Prevalence and correlates of burnout among Lebanese health care workers during the COVID-19 pandemic: a national cross-sectional survey.](https://pubmed-ncbi-nlm-nih-gov.proxy.kib.ki.se/36527056/) J Pharm Policy Pract. 2022;15(1):102. doi: 10.1186/s40545-022-00503-2

-----------------------------

77.Zheng Y, Tang PK, Lin G, Liu J, Hu H, Wu AMS, Ung COL. [Burnout among healthcare providers: Its prevalence and association with anxiety and depression during the COVID-19 pandemic in Macao, China.](https://pubmed-ncbi-nlm-nih-gov.proxy.kib.ki.se/36928867/) PLoS One. 2023;18(3):e0283239. doi: 10.1371/journal.pone.0283239.

**Study Branch 2: Studies applying the Occupational Stressor Index among Physicians**

Search strategy: (Occupational Stressor Index) AND (health professionals)

------------------------------

1.Belkić K, Nedić O. Workplace stressors and lifestyle-related cancer risk factors among female physicians: Assessment using the occupational stress index. J Occup Health 2007; 49 (1): 61-71. doi: 10.1539/joh.49.61

------------------------------

2.Belkić K, Nedić O. Night work, total occupational burden and cancer/cardiovascular risk factors in physicians. Med Pregl 2012; 65(11-12): 461-9.

------------------------------

3.Belkić K, Nedić O. Occupational medicine—then and now: Where we could go from here. Med Pregl 2014; 67(5-6): 139-147.

---------------------------

4.Belkić K, Nedić O. Physician health challenges and return to work—insights from participatory action research for physicians by physicians. Med Pregl. 2019; 72(11-12): 367–73.

---------------------------

5. Belkić K, Rustagi N. Job stressors in relation to burnout and compromised sleep among academic physicians in India. Work. 2024; 78: 505–525.

------------------------------

6.Belkić K, Savić C. Job stressors and mental health: A proactive clinical perspective. Toh Tuck (Singapore): World Scientific; 2013.

---------------------------

7.Djindjić N, Jovanović J, Djindjić B, Jovanović M, Jovanović JJ. Associations between the Occupational Stress Index and hypertension, type 2 diabetes mellitus, and lipid disorders in middle-aged men and women. Ann Occup Hyg 2012; 56(9): 1051 –62.

---------------------------

8.Emdad R, Belkić K, Theorell T, Cizinsky S, What prevents professional drivers from following physicians' cardiologic advice? Psychoth Psychosom 1998; 67: 226-240

-----------------------------

9.Goyal P, Rustagi N, Belkić K. Physicians’ total burden of occupational stressors: Over three-fold increased odds of burnout. South Med J. 2021; 114(7): 409-415.

-----------------------------

10. Jovanović J, Stefanović V, Stanković DN, Bogdanović D, Kocić B, Jovanović M, et al. [Serum lipids and glucose disturbances at professional drivers exposed to occupational stressors.](https://pubmed-ncbi-nlm-nih-gov.proxy.kib.ki.se/18661806/) Cent Eur J Public Health. 2008;16(2):54-8. doi: 10.21101/cejph.a3451.

-----------------------------

11.Khoa L, Nhat Quang T, Quang Vinh D, Thi Ngoc Anh N, Manh Tuong H, Foster K. The prevalence of job stressors among nurses in private in vitro fertilization (IVF) centres. Nursing Open. 2019; 6: 39–49

-----------------------------

11.Matić M, Jovanović J, Jovanović J, Macivanin N. [[Effects of occupational stress on working ability of patients suffering from arterial hypertension].](https://pubmed-ncbi-nlm-nih-gov.proxy.kib.ki.se/24575639/) Med Pregl. 2013;66(11-12):497-501. doi: 10.2298/mpns1312497m. [Serbian]

-----------------------------

13.Nedić O. Occupational stressors and physician health with a focus upon cardiovascular disease. Dissertation, University of Novi Sad. 2006.

-----------------------------

14.Nedić O, Belkić K, Filipović D, Jocić N, Job stressors among female physicians: Relation to having a clinical diagnosis of hypertension, Int J Occup Environ Med 2010, 16: 330-340.

-----------------------------

15.Nedić O, Belkić K, Filipović D, Jocić N, Work stressors among physicians with the acquired cardiovascular disorders. Assessment using the occupational stress index, Med Pregl 2008; 61: 226-234.

-----------------------------

16.Nedić O, Belkić K, Filipović D, Jocić N, Gender as an important effect modifier between exposure to work stressors among physicians of various specialties and the occurence of cardiovascular disease, Med Pregl 2008; 61: 343-349.

-----------------------------

17.Nedić O, Belkić K. Job stressors and burnout among nurses and primary-care physicians working at a dedicated outpatient respiratory center for patients with suspected or confirmed COVID-19. Am J Ind Med. 2023; 66 (6): 510-28.

-----------------------------

18.Othman CN, Lamin RAC, Othman N. Occupational stress index in the Malaysian university workplace. Procedia Social Behav Sci. 2014; 153: 700-10.

-----------------------------

19.Savić Č, Belkić K. Why are job stressors relevant for psychiatry? Br J Psychiatry. 2014; 205(6): 425-427.

-----------------------------

20.Soori H, Rahimi M, Mohseni H. [Occupational stress and work-related unintentional injuries among Iranian car manufacturing workers.](https://pubmed-ncbi-nlm-nih-gov.proxy.kib.ki.se/18720634/) East Mediterr Health J. 2008;14(3):697-703.
