# Supplementary material for: Toward better prevention of physician burnout: insights from individual participant data using the MD-specific Occupational Stressor Index and organizational interventions
Source: Front Public Health. 2025 Mar 19;13:1514706. doi: 10.3389/fpubh.2025.1514706 (PMC11961930; doi:10.3389/fpubh.2025.1514706)
Supplement: Supplementary file 2 [file Data_Sheet_2.docx]

**Supplement 2: Search for Publications on the OSI Stressors showing Multivariable Association with**

**the Copenhagen Burnout Index among Physicians in the Individual Participant Data Analysis**

**C. WORK HOURS & SCHEDULING**

**Workdays per week or Work hours per Week**

Search strategy: [(workdays per week) or (day free from work)] AND (Physician or Health care professional) AND (burnout)

----------------------------------

Ali NA, Hammersley J, Hoffmann SP, O'Brien JM Jr, Phillips GS, Rashkin M, Warren E, Garland A; Midwest Critical Care Consortium. [Continuity of care in intensive care units: a cluster-randomized trial of intensivist staffing.](https://pubmed-ncbi-nlm-nih-gov.proxy.kib.ki.se/21719756/) Am J Respir Crit Care Med. 2011;184(7):803-8. doi: 10.1164/rccm.201103-0555OC

----------------------------------

Aljabri D, Alshatti F, Alumran A, Al-Rayes S, Alsalman D, Althumairi A, et al. Sociodemographic and occupational factors associated with burnout: a study among frontline healthcare workers during the COVID-19 pandemic. Front. Public Health. 2022; 10: 54687 doi: 10.3389/fpubh.2022.854687

----------------------------------

Alrawashdeh HM, Al-Tammemi AB, Alzawahreh MK, Al-Tamimi A, Elkholy M, Al Sarireh F, et al. [Occupational burnout and job satisfaction among physicians in times of COVID-19 crisis: a convergent parallel mixed-method study.](https://pubmed-ncbi-nlm-nih-gov.proxy.kib.ki.se/33906619/) BMC Public Health. 2021 Apr 28;21(1):811. doi: 10.1186/s12889-021-10897-4.

----------------------------------

Alsulimani LK, Farhat AM, Borah RA, AlKhalifah JA, Alyaseen SM, Alghamdi SM, et al. Health care worker burnout during the COVID-19 pandemic: A cross-sectional survey study in Saudi Arabia. Saudi Med J. 2021; 42(3):306-14.

----------------------------------

Ashraf F, Ahmad H, Aftab S. [Interactive role of personal and work related factors in psychological burnout: A study of Pakistani doctors.](https://pubmed-ncbi-nlm-nih-gov.proxy.kib.ki.se/32794497/)

J Pak Med Assoc. 2020;70(8):1413-7.

----------------------------------

Barrack RL, Miller LS, Sotile WM, Sotile MO, Rubash HE. Effect of duty hour standards on burnout among orthopaedic surgery residents. Clin Orthop Relat Res. 2006; 449:134–7.

----------------------------------

Benson S, Sammour T, Neuhaus SJ, Findlay B, Hill AG. [Burnout in Australasian younger fellows.](https://pubmed-ncbi-nlm-nih-gov.proxy.kib.ki.se/19895513/) ANZ J Surg. 2009;79(9):590-7 doi: 10.1111/j.1445-2197.2009.05012.x.

----------------------------------

Berger B, Cungi PJ, Arzalier S, Lieutaud T, Velly L, Simeone P, et al. [Incidence of burnout syndrome among anesthesiologists and intensivists in France: The REPAR Study.](https://pubmed-ncbi-nlm-nih-gov.proxy.kib.ki.se/36767139/) Int J Environ Res Public Health. 2023;20(3):1771. doi: 10.3390/ijerph20031771.

----------------------------------

Biddle GJ, Thomas N, Edwardson CL, Clemes SA, Daley AJ. [Burnout, psychological wellbeing, and musculoskeletal complaints in UK GPs: an observational study.](https://pubmed-ncbi-nlm-nih-gov.proxy.kib.ki.se/37474254/) BJGP Open. 2023;7(4):BJGPO.2023.0007. doi: 10.3399/BJGPO.2023.0007.

----------------------------------

Blum AB, Shea S, Czeisler CA, Landrigan CP, Leape L. [Implementing the 2009 Institute of Medicine recommendations on resident physician work hours, supervision, and safety.](https://pubmed-ncbi-nlm-nih-gov.proxy.kib.ki.se/23616719/) Nat Sci Sleep. 2011;3:47-85. doi: 10.2147/NSS.S19649.

----------------------------------

Chambers CN, Frampton CM, Barclay M, McKee M. [Burnout prevalence in New Zealand's public hospital senior medical workforce: A cross-sectional mixed methods study.](https://pubmed-ncbi-nlm-nih-gov.proxy.kib.ki.se/27881531/) BMJ Open. 2016; 6(11):e013947. doi: 10.1136/bmjopen-2016-013947.

----------------------------------

Chu WM, Ho HE, Lin YL, Li JY, Lin CF, Chen CH, et al. Risk factors surrounding an increase in burnout and depression among health care professionals in Taiwan during the COVID-19 pandemic. Am Med Dir Assoc 2023; 24(2):164-70. https://doi.org/10.1016/j.jamda.2022.12.010

----------------------------------

Correia C, Teixeira R, de Almeida NMP, Morais S, Figueiredo P. [Burnout in gastrenterologists: a national-level analysis.](https://pubmed-ncbi-nlm-nih-gov.proxy.kib.ki.se/34403305/) Scand J Gastroenterol. 2021;56(11):1271-8. doi: 10.1080/00365521.2021.1961308.

----------------------------------

Dale J, Potter R, Owen K, Parsons N, Realpe A, Leach J. R[etaining the general practitioner workforce in England: what matters to GPs? A cross-sectional study.](https://pubmed-ncbi-nlm-nih-gov.proxy.kib.ki.se/26475707/) BMC Fam Pract. 2015;16:140 doi: 10.1186/s12875-015-0363-1.

----------------------------------

Estryn-Behar M, Fry C, Guetarni K, Aune I, Machet G, Doppia MA, et al. [Work week duration, work-family balance and difficulties encountered by female and male physicians: results from the French SESMAT study.](https://pubmed-ncbi-nlm-nih-gov.proxy.kib.ki.se/22112665/) Work. 2011;40 Suppl 1:S83-100. doi: 10.3233/WOR-2011-1270.

----------------------------------

Estephan L, Pu C, Bermudez S, Waits A. Burnout, mental health, physical symptoms, and coping behaviors in healthcare workers in Belize amidst COVID-19 pandemic: A nationwide cross-sectional study.Int J Soc Psychiatry. 2023;69(4):1033-1042. doi: 10.1177/00207640231152209

----------------------------------

Ferguson CM, Kellogg KC, Hutter MM, Warshaw AL. [Effect of work-hour reforms on operative case volume of surgical residents.](https://pubmed-ncbi-nlm-nih-gov.proxy.kib.ki.se/16125616/)Curr Surg. 2005;62(5):535-8. **(OSI** doi: 10.1016/j.cursur.2005.04.001.

----------------------------------

Fonseca M, Sanclemente G, Hernández C, Visiedo C, Bragulat E, Miró O. [Residents, duties and burnout syndrome.](https://pubmed-ncbi-nlm-nih-gov.proxy.kib.ki.se/20381031/)Rev Clin Esp. 2010;210(5):209-15. doi: 10.1016/j.rce.2009.12.006.

----------------------------------

Gelfand DV, Podnos YD, Carmichael JC,Saltzman DJ, Wilson SE, Williams RA. Effect of the 80-hour workweek on resident burnout. Arch Surg 2004;139(9):933–40.

----------------------------------

Geva A, Landrigan CP, van der Velden MG, Randolph AG. [Simulation of a novel schedule for Intensivist staffing to improve continuity of patient care and reduce physician burnout.](https://pubmed-ncbi-nlm-nih-gov.proxy.kib.ki.se/28362643/) Crit Care Med. 2017;45(7):1138-44. doi: 10.1097/CCM.0000000000002319.

----------------------------------

Goitein L, Shanafelt TD, Wipf JE, Slatore CG, Back AL. The effects of work-hour limitations on resident well-being, patient care, and education in an internal medicine residency program. Arch Intern Med. 2005;165(22):2601–6

----------------------------------

Gonçalves JV, Castro L, Nunes R, Rêgo G. [Burnout among physicians working in palliative care during the COVID-19 Pandemic in Portugal: A cross-sectional study.](https://pubmed-ncbi-nlm-nih-gov.proxy.kib.ki.se/36898212/) Acta Med Port. 2023;36(3):183-92. doi: 10.20344/amp.18361.

----------------------------------

Gopal R, Glasheen JJ, Miyoshi TJ, Prochazka AV. Burnout and internal medicine resident work-hour restrictions. Arch Intern Med. 2005;165(22):2595–600.

----------------------------------

Harkin D, Alzayyat S, Kiernan A, Ryan Á, Boland F, Renton S, et al. [Vascular surgeon burnout and resilience in the United Kingdom: A Report from the Vascular Society Workforce Committee.](https://pubmed-ncbi-nlm-nih-gov.proxy.kib.ki.se/35248742/) Ann Vasc Surg. 2022;84:169-78. doi: 10.1016/j.avsg.2022.01.032.

----------------------------------

Hutter MM, Kellogg KC, Ferguson CM, Abbott WM, Warshaw AL. The impact of the 80-hour resident workweek on surgical residents and attending surgeons. Ann Surg. 2006;243(6):864–71.

----------------------------------

Isaksson Rø KE, Gude T, Tyssen R, Aasland OG. Counseling for burnout in Norwegian doctors: One year cohort study. BMJ. 2008;337(7679):1146-9.

----------------------------------

Jones CE, Fox ED, Holsten SB, White CQ, Sayyid RK, O'Keeffe T, et al. [Burnout reduction in acute care surgeons: Impact of faculty schedule change at a level 1 trauma and tertiary care center.](https://pubmed-ncbi-nlm-nih-gov.proxy.kib.ki.se/35788582/)J Trauma Acute Care Surg. 2022;93(4):439-45. doi: 10.1097/TA.0000000000003736.

----------------------------------

Jung FU, Bodendieck E, Bleckwenn M, Hussenoeder FS, Luppa M, Riedel-Heller SG. [Burnout, work engagement and work hours - how physicians' decision to work less is associated with work-related factors.](https://pubmed-ncbi-nlm-nih-gov.proxy.kib.ki.se/36793035/) BMC Health Serv Res. 2023;23(1):157. doi: 10.1186/s12913-023-09161-9.

----------------------------------

Kurzthaler I, Kemmler G, Holzner B, Hofer A. [Physician's burnout and the COVID-19 Pandemic-A nationwide cross-sectional study in Austria.](https://pubmed-ncbi-nlm-nih-gov.proxy.kib.ki.se/34950073/) Front Psychiatry. 2021;12:784131. doi: 10.3389/fpsyt.2021.784131

----------------------------------

Lapa T, Carvalho S, Viana J, Ferreira PL, Pinto-Gouveia J, Cabete AB. [Development and evaluation of a Global Burnout Index derived from the use of the Copenhagen Burnout Inventory in Portuguese physicians.](https://pubmed-ncbi-nlm-nih-gov.proxy.kib.ki.se/30387421/) Acta Med Port. 2018;31(10):534-41.doi: 10.20344/amp.10407.

----------------------------------

Lefebvre DC. [Perspective: Resident physician wellness: a new hope.](https://pubmed-ncbi-nlm-nih-gov.proxy.kib.ki.se/22450179/) Acad Med. 2012;87(5):598-602. doi: 10.1097/ACM.0b013e31824d47ff.

----------------------------------

Lin RT, Lin YT, Hsia YF, Kuo CC. Long working hours and burnout in health care workers: Non-linear dose-response relationship and the effect mediated by sleeping hours-A cross-sectional study. J Occup Health. 2021;63(1):e12228. doi: 10.1002/1348-9585.12228.

----------------------------------

Lue BH, Chen HJ, Wang CW, Cheng Y, Chen MC.  [Stress, personal characteristics and burnout among first postgraduate year residents: A nationwide study in Taiwan.](https://pubmed-ncbi-nlm-nih-gov.proxy.kib.ki.se/20423259/) Med Teach. 2010;32(5):400-7. doi: 10.3109/01421590903437188.

----------------------------------

Martini S, Arfken CL, Balon R. Comparison of burnout among medical residents before and after the implementation of work hours limits. Acad Psychiatry. 2006; 30(4): 352–55. Doi: 10.1176/appi.ap.352.

----------------------------------

McEntee K, Koenig H, Hattigangadi R, Loring M, Brockmeyer A, Dahlman M. Factors associated with burnout among minimally invasive gynecologic surgery fellows. AJOG Glob Rep. 2022; 2(3): 100074. doi.org/10.1016/j.xagr.2022.100074

----------------------------------

McGowan Y, Humphries N, Burke H, Conry M, Morgan K. [Through doctors' eyes: A qualitative study of hospital doctor perspectives on their working conditions.](https://pubmed-ncbi-nlm-nih-gov.proxy.kib.ki.se/23480457/) Br J Health Psychol. 2013;18(4):874-91. doi: 10.1111/bjhp.12037.

----------------------------------

Nimer A, Naser S, Sultan N, Alasad RS, Rabadi A, Abu-Jubba M, et al. [Burnout syndrome during residency training in Jordan: Prevalence, risk factors, and implications.](https://pubmed-ncbi-nlm-nih-gov.proxy.kib.ki.se/33562100/) Int J Environ Res Public Health. 2021 Feb 6;18(4):1557. doi: 10.3390/ijerph18041557.

----------------------------------

Nitzsche A, Neumann M, Groß SE, Ansmann L, Pfaff H, Baumann W, et al. [Recovery opportunities, work-home conflict, and emotional exhaustion among hematologists and oncologists in private practice.](https://pubmed-ncbi-nlm-nih-gov.proxy.kib.ki.se/27652494/) Psychol Health Med. 2017;22(4):462-473**.**) doi: 10.1080/13548506.2016.1237666.

----------------------------------

O'Brien DC, Carr MM. [Current wellness practices among otolaryngology residencies.](https://pubmed-ncbi-nlm-nih-gov.proxy.kib.ki.se/29920216/) Otolaryngol Head Neck Surg. 2018;159(2):258-65. doi: 10.1177/0194599818782408.

----------------------------------

Ovalle Diaz J, Gorgen ARH, Teixeira da Silva AG, de Oliveira Paludo A, Timóteo de Oliveira R, Rosito N, et al. [Burnout syndrome in pediatric urology: A perspective during the COVID-19 pandemic - Ibero-American survey.](https://pubmed-ncbi-nlm-nih-gov.proxy.kib.ki.se/33602610/) J Pediatr Urol. 2021;17(3):402.e1-402.e7. doi: 10.1016/j.jpurol.2021.01.015.

----------------------------------

Pius RE, Ajuluchukwu JN, Roberts AA. [Prevalence and correlates of burn-out among Nigerian medical doctors during the COVID-19 pandemic: a cross-sectional study.](https://pubmed-ncbi-nlm-nih-gov.proxy.kib.ki.se/37996233/) BMJ Open. 2023;13(11):e076673. doi: 10.1136/bmjopen-2023-076673.

----------------------------------

Quirk R, Rodin H, Linzer M.  [Targeting causes of burnout in residency: An innovative approach used at Hennepin healthcare.](https://pubmed-ncbi-nlm-nih-gov.proxy.kib.ki.se/33496434/) Acad Med. 2021;96(5):690-4.

----------------------------------

Ripp JA, Bellini L, Fallar R, Bazari H, Katz JT, Korenstein D. The impact of duty hours restrictions on job burnout in internal medicine residents: A 3-institution comparison study. Acad Med. 2015;90(4):494–9.

----------------------------------

Saijo Y, Chiba S, Yoshioka E, Kawanishi Y, Nakagi Y, Itoh T, et al. [Effects of work burden, job strain and support on depressive symptoms and burnout among Japanese physicians.](https://pubmed-ncbi-nlm-nih-gov.proxy.kib.ki.se/25503892/) Int J Occup Med Environ Health. 2014;27(6):980-92. doi: 10.2478/s13382-014-0324-2.

----------------------------------

Shoureshi P, Guerre M, Seideman CA, Callejas DG, Amling CL, Bassale S, et al.  [Addressing Burnout in urology: A qualitative assessment of interventions.](https://pubmed-ncbi-nlm-nih-gov.proxy.kib.ki.se/37145567/) Urol Pract. 2022;9(1):101-7.

----------------------------------

Sturm H, Rieger MA, Martus P, Ueding E, Wagner A, Holderried M, et al. [Do perceived working conditions and patient safety culture correlate with objective workload and patient outcomes: A cross-sectional explorative study from a German university hospital.](https://pubmed-ncbi-nlm-nih-gov.proxy.kib.ki.se/30608945/) PLoS One. 2019;14(1):e0209487. doi: 10.1371/journal.pone.0209487.

----------------------------------

Toyoshima M, Takenoshita S, Hasegawa H, Kimura T, Nomura K. E[xperiences of negotiations for improving research environment and burnout among young physician researchers in Japan.](https://pubmed-ncbi-nlm-nih-gov.proxy.kib.ki.se/32698340/) Int J Environ Res Public Health. 2020;17(14):5221. doi: 10.3390/ijerph17145221.

----------------------------------

Tsai YL, Tung YC, Cheng Y. [Surveys of burnout among physicians in Taiwan.](https://pubmed-ncbi-nlm-nih-gov.proxy.kib.ki.se/32995210/) J Acute Med. 2018;8(3):86-98. doi: 10.6705/j.jacme.201809_8(3).0002.

----------------------------------

West CP, Dyrbye LN, Erwin PJ, Shanafelt TD. Interventions to prevent and reduce physician burnout: a systematic review and meta-analysis. Lancet 2016; 388: 2272–81.

Wright JG, Khetani N, Stephens D. [Burnout among faculty physicians in an academic health science centre.](https://pubmed-ncbi-nlm-nih-gov.proxy.kib.ki.se/22851895/) Paediatr Child Health. 2011;16(7):409-13. doi: 10.1093/pch/16.7.409.

----------------------------------

Youssef D, Abboud E, Abou-Abbas L, Hassan H, Youssef J. [Prevalence and correlates of burnout among Lebanese health care workers during the COVID-19 pandemic: a national cross-sectional survey.](https://pubmed-ncbi-nlm-nih-gov.proxy.kib.ki.se/36527056/) J Pharm Policy Pract. 2022;15(1):102. doi: 10.1186/s40545-022-00503-2

----------------------------------

Zink K, Clugston C, Regan L.  [Hopes and fears: A qualitative analysis of the intern perspective at the start of EM residency.](https://pubmed-ncbi-nlm-nih-gov.proxy.kib.ki.se/35774535/)AEM Educ Train. 2022;6(3):e10764. doi: 10.1002/aet2.10764.

----------------------------------------------------------------------------------------------------------------------------------------------------------------------------------------------------------------

----------------------------------------------------------------------------------------------------------------------------------------------------------------------------------------------------------------

**Called/emailed during free time about patients or other work**

Search strategy: (outside work hours) AND [(Physician or Health care professional)[ AND [(burnout)]

----------------------------------

Abduljabbar FH, Teles AR, Ouellet JA, Ferland CE, Wong CC, Barbagallo G,et al. [Spine surgeons burnout and quality of life: Results of a worldwide survey.](https://pubmed-ncbi-nlm-nih-gov.proxy.kib.ki.se/34559753/) Spine (Phila Pa 1976). 2021;46(20):1418-1927. doi: 10.1097/BRS.0000000000004038.

--------------------------

Akbar F, Mark G, Prausnitz S, Warton EM, East JA, Moeller MF, Reed ME, Lieu TA. [Physician stress during electronic health record inbox work: In Situ measurement with wearable sensors.](https://pubmed-ncbi-nlm-nih-gov.proxy.kib.ki.se/33908888/) JMIR Med Inform. 2021;9(4):e24014 doi: 10.2196/24014.

----------------------------------------------------------

Arndt BG, Beasley JW, Watkinson MD, et al. Tethered to the EHR: primary care physician workload assessment using EHR Event log data and time-motion

observations. Ann Fam Med. 2017; 15(5): 419-26. 10.1370/afm.2121

----------------------------------------------------------

Arndt BG, Micek MA, Rule A, Shafer CM, Baltus JJ, Sinsky CA. [More tethered to the EHR: EHR workload trends among academic primary care physicians, 2019-2023.](https://pubmed-ncbi-nlm-nih-gov.proxy.kib.ki.se/38253499/) Ann Fam Med. 2024;22(1):12-18. doi: 10.1370/afm.3047.

----------------------------------------------------------

Aziz F, Talhelm L, Keefer J, Krawiec C.  [Vascular surgery residents spend one fifth of their time on electronic health records after duty hours.](https://pubmed-ncbi-nlm-nih-gov.proxy.kib.ki.se/31010521/)

J Vasc Surg. 2019;69(5):1574-9. doi: 10.1016/j.jvs.2018.08.173.

----------------------------------------------------------

Bahr TJ, Ginsburg S, Wright JG, Shachak A.  [Technostress as source of physician burnout: An exploration of the associations between technology usage and physician burnout.](https://pubmed-ncbi-nlm-nih-gov.proxy.kib.ki.se/37517300/) Int J Med Inform. 2023;177:105147. doi: 10.1016/j.ijmedinf.2023.105147.

----------------------------------------------------------

Bali AS, Hashash JG, Picco MF, Kinnucan JA, Farraye FA. [Electronic health record burden among gastroenterology providers associated with subspecialty and training.](https://pubmed-ncbi-nlm-nih-gov.proxy.kib.ki.se/36940436/) Am J Gastroenterol. 2023 Jul 1;118(7):1282-4. doi: 10.14309/ajg.0000000000002254.

----------------------------------------------------------

Banerjee S, Lim KHJ, Murali K, Kamposioras K, Punie K, Oing C, et al. [The impact of COVID-19 on oncology professionals: Results of the ESMO Resilience Task Force survey collaboration.](https://pubmed-ncbi-nlm-nih-gov.proxy.kib.ki.se/33601295/)ESMO Open. 2021;6(2):100058.**)**  doi: 10.1016/j.esmoop.2021.100058.

----------------------------------------------------------

Brainch N, Schule P, Laurel F, Bodic M, Jacob T. [Psychiatric emergency services - can duty-hour changes help residents and patients?](https://pubmed-ncbi-nlm-nih-gov.proxy.kib.ki.se/29654393/) Psychiatr Q. 2018;89(4):771-8. doi: 10.1007/s11126-018-9579-2.

--------------------

Brennan J, McGrady A. [Designing and implementing a resiliency program for family medicine residents.](https://pubmed-ncbi-nlm-nih-gov.proxy.kib.ki.se/26130769/) Int J Psychiatry Med. 2015;50(1):104-14. doi: 10.1177/0091217415592369.

----------------------------------------------------------

Brown CVR, Joseph BA, Davis K, Jurkovich GJ. [Modifiable factors to improve work-life balance for trauma surgeons.](https://pubmed-ncbi-nlm-nih-gov.proxy.kib.ki.se/32925572/) J Trauma Acute Care Surg. 2021;90(1):122-8. doi: 10.1097/TA.0000000000002910.

----------------------------------------------------------

Budd J. [Burnout related to electronic health record use in primary care.](https://pubmed-ncbi-nlm-nih-gov.proxy.kib.ki.se/37073905/) J Prim Care Community Health. 2023;14:21501319231166921. doi: 10.1177/21501319231166921

----------------------------------------------------------

Cankurtaran CZ, Reddy S, Cen SY, Lei X, Walker DK.  [Work-life experience of academic radiologists: Food for thought.](https://pubmed-ncbi-nlm-nih-gov.proxy.kib.ki.se/36775667/) Acad Radiol. 2023;30(4):579-84. doi: 10.1016/j.acra.2023.01.011.

----------------------------------------------------------

Cheesborough JE, Gray SS, Bajaj AK.  [Striking a better integration of work and life: Challenges and solutions.](https://pubmed-ncbi-nlm-nih-gov.proxy.kib.ki.se/28125538/) Plast Reconstr Surg. 2017 Feb;139(2):495-500. doi: 10.1097/PRS.0000000000002955.

--------------------------

Cox ML, Farjat AE, Risoli TJ, Peskoe S, Goldstein BA, Turner DA, et al. [Documenting or operating: Where is time spent in general surgery residency?](https://pubmed-ncbi-nlm-nih-gov.proxy.kib.ki.se/30522828/)

J Surg Educ. 2018; 75(6):e97-e106doi: 10.1016/j.jsurg.2018.10.010. Migaly J.

--------------------------

Cross DA, Holmgren AJ, Apathy NC. [The role of organizations in shaping physician use of electronic health records.](https://pubmed-ncbi-nlm-nih-gov.proxy.kib.ki.se/37438938/) Health Serv Res. 2024;59(1):e14203. doi: 10.1111/1475-6773.14203.

--------------------

Demirel Öğüt N, Öğüt Ç, Eşme P. [The role of online consultation requests to personal social media accounts and instant messaging services of dermatologists in occupational burnout: An emerging problem.](https://pubmed-ncbi-nlm-nih-gov.proxy.kib.ki.se/34449960/) J Cosmet Dermatol. 2022;21(6):2542-9.**)**  doi: 10.1111/jocd.14417.

--------------------

Dyrbye LN, Gordon J, O'Horo J, Belford SM, Wright M, Satele DV, West CP. [Relationships between EHR-based audit log data and physician burnout and clinical practice process measures.](https://pubmed-ncbi-nlm-nih-gov.proxy.kib.ki.se/36868747/) Mayo Clin Proc. 2023;98(3):398-409. doi: 10.1016/j.mayocp.2022.10.027.

----------------------

Dyrbye LN, West CP, Sinsky CA, Trockel M, Tutty M, Satele D, et al.  [Physicians' experiences with mistreatment and discrimination by patients, families, and visitors and association with burnout.](https://pubmed-ncbi-nlm-nih-gov.proxy.kib.ki.se/35587344/) JAMA Netw Open. 2022;5(5):e2213080. doi: 10.1001/jamanetworkopen.2022.13080.

----------------------------

Fattori A, Pedruzzi M, Cantarella C, Bonzini M. [The burden in palliative care assistance: A comparison of psychosocial risks and burnout between inpatient hospice and home care services workers.](https://pubmed-ncbi-nlm-nih-gov.proxy.kib.ki.se/35078551/) Palliat Support Care. 2023;21(1):49-56. doi: 10.1017/S1478951521001887.

----------------------------

Ferrari L, Mari V, De Santi G, Parini S, Capelli G, Tacconi G, et al. [Early barriers to career progression of women in surgery and solutions to improve them: a systematic scoping review.](https://pubmed-ncbi-nlm-nih-gov.proxy.kib.ki.se/35797642/) Ann Surg. 2022;276(2):246-55. doi: 10.1097/SLA.0000000000005510.

--------------------

Fischer R, Mattos P, Teixeira C, Ganzerla DS, Rosa RG, Bozza FA.  [Association of burnout with depression and anxiety in critical care clinicians in Brazil.](https://pubmed-ncbi-nlm-nih-gov.proxy.kib.ki.se/33355676/)

JAMA Netw Open. 2020;3(12):e2030898. doi: 10.1001/jamanetworkopen.2020.30898.

---------------------------------

Gajra A, Bapat B, Jeune-Smith Y, Nabhan C, Klink AJ, Liassou D, Mehta S, Feinberg B. [Frequency and causes of burnout in US community oncologists in the era of electronic health records.](https://pubmed-ncbi-nlm-nih-gov.proxy.kib.ki.se/32275848/) JCO Oncol Pract. 2020;16(4):e357-e365. doi: 10.1200/JOP.19.00542.

----------------------

Garcia LC, Shanafelt TD, West CP, Sinsky CA, Trockel MT, Nedelec L, et al. [Burnout, depression, career satisfaction, and work-life integration by physician race/ethnicity.](https://pubmed-ncbi-nlm-nih-gov.proxy.kib.ki.se/32766802/) JAMA Netw Open. 2020; 3(8):e2012762. doi: 10.1001/jamanetworkopen.2020.12762.

---------------------------

Goyal P, Rustagi N, Belkić K.  [Physicians' total burden of occupational stressors: More than threefold increased odds of burnout.](https://pubmed-ncbi-nlm-nih-gov.proxy.kib.ki.se/34215893/)

South Med J. 2021;114(7):409-15. doi: 10.14423/SMJ.0000000000001277.

----------------------------------------------------------

Grunfeld E, Whelan TJ, Zitzelsberger L, Willan AR, Montesanto B, Evans WK. [Cancer care workers in Ontario: prevalence of burnout, job stress and job satisfaction.](https://pubmed-ncbi-nlm-nih-gov.proxy.kib.ki.se/10934978/) CMAJ. 2000;163(2):166-9.

----------------------------------------------------------

Harry EM, Carlasare LE, Sinsky CA, Brown RL, Goelz E, Nankivil N, et al. [Childcare stress, burnout, and intent to reduce hours or leave the job during the COVID-19 Pandemic Among US health care workers.](https://pubmed-ncbi-nlm-nih-gov.proxy.kib.ki.se/35849398/) JAMA Netw Open. 2022;5(7):e2221776. doi: 10.1001/jamanetworkopen.2022.21776.

----------------------------------------------------------

Holmgren AJ, Apathy NC. Assessing the impact of patient access to clinical notes on clinician EHR documentation. J Am Med Inform Assoc. 2022; 29(10): 1733-6. doi.org/10.1093/jamia/ocac120

----------------------------------------------------------

Holmgren AJ, Thombley R, Sinsky CA, Adler-Milstein J. [Changes in physician electronic health record use with the expansion of telemedicine.](https://pubmed-ncbi-nlm-nih-gov.proxy.kib.ki.se/37902737/) JAMA Intern Med. 2023;183(12):1357-65. doi: 10.1001/jamainternmed.2023.5738.

---------------------------

Khairat S, Coleman C, Ottmar P, Jayachander DI, Bice T, Carson SS. [Association of electronic health record use with physician fatigue and efficiency.](https://pubmed-ncbi-nlm-nih-gov.proxy.kib.ki.se/32515799/) JAMA Netw Open. 2020;3(6):e207385. doi: 10.1001/jamanetworkopen.2020.7385.

--------------------------

Lai R, Teoh K, Plakiotis C. [Factors contributing to stress and well-being among trainee psychiatrists in Victoria, Australia.](https://pubmed-ncbi-nlm-nih-gov.proxy.kib.ki.se/37581784/) Adv Exp Med Biol. 2023;1425:93-104. doi: 10.1007/978-3-031-31986-0_9.

--------------------------

Lund S, D'Angelo AL, Busch R, Friberg R, D'Angelo J. [With a little help from my friends: The negating impact of social community and mentorship on burnout.](https://pubmed-ncbi-nlm-nih-gov.proxy.kib.ki.se/35605571/) J Surg Res. 2022;278:190-95. doi: 10.1016/j.jss.2022.04.062.

-------------------------------

McGowan Y, Humphries N, Burke H, Conry M, Morgan K. [Through doctors' eyes: A qualitative study of hospital doctor perspectives on their working conditions.](https://pubmed-ncbi-nlm-nih-gov.proxy.kib.ki.se/23480457/) Br J Health Psychol. 2013;18(4):874-91. doi: 10.1111/bjhp.12037.

--------------------------

Melnick ER, Fong A, Nath B, Williams B, Ratwani RM, Goldstein R, et al.  [Analysis of electronic health record use and clinical productivity and their association with physician turnover.](https://pubmed-ncbi-nlm-nih-gov.proxy.kib.ki.se/34636911/) JAMA Netw Open. 2021 Oct 1;4(10):e2128790. doi: 10.1001/jamanetworkopen.2021.28790.

------------------------------

Molwitz I, Kemper C, Stahlmann K, Oechtering TH, Sieren MM, Afat S, et al. [Work expectations, their fulfillment, and exhaustion among radiologists of all career levels: What can be learned from the example of Germany.](https://pubmed-ncbi-nlm-nih-gov.proxy.kib.ki.se/36897346/) Eur Radiol. 2023;33(8):5664-74. doi: 10.1007/s00330-023-09510-6.

------------------------

Nedić O, Belkić K. [Job stressors and burnout among nurses and primary-care physicians working at a dedicated outpatient respiratory center for patients with suspected or confirmed COVID-19.](https://pubmed-ncbi-nlm-nih-gov.proxy.kib.ki.se/37013937/) Am J Ind Med. 2023;66(6):510-28. doi: 10.1002/ajim.23475.

----------------------

Nitzsche A, Neumann M, Groß SE, Ansmann L, Pfaff H, Baumann W, et al. [Recovery opportunities, work-home conflict, and emotional exhaustion among hematologists and oncologists in private practice.](https://pubmed-ncbi-nlm-nih-gov.proxy.kib.ki.se/27652494/) Psychol Health Med. 2017;22(4):462-473**.** doi: 10.1080/13548506.2016.1237666.

------------------------

Peccoralo LA, Kaplan CA, Pietrzak RH, Charney DS, Ripp JA. [The impact of time spent on the electronic health record after work and of clerical work on burnout among clinical faculty.](https://pubmed-ncbi-nlm-nih-gov.proxy.kib.ki.se/33550392/) J Am Med Inform Assoc. 2021;28(5):938-47 doi: 10.1093/jamia/ocaa349.

---------------------------

Pourmand K, Schiano TD, Motwani Y, Kriss M, Keefer L, Patel A. [Burnout Among transplant hepatologists in the United States.](https://pubmed-ncbi-nlm-nih-gov.proxy.kib.ki.se/34826182/) Liver Transpl. 2022;28(5):867-75. doi: 10.1002/lt.26375.

--------------------------

Quirk R, Rodin H, Linzer M.  [Targeting causes of burnout in residency: An innovative approach used at Hennepin healthcare.](https://pubmed-ncbi-nlm-nih-gov.proxy.kib.ki.se/33496434/) Acad Med. 2021;96(5):690-4.

--------------------------

Reliford A, Adebanjo B. [Use of Telepsychiatry in pediatric emergency room to decrease length of stay for psychiatric patients, improve resident on-call burden, and reduce factors related to physician burnout.](https://pubmed-ncbi-nlm-nih-gov.proxy.kib.ki.se/30379635/) Telemed J E Health. 2019;25(9):828-832. doi: 10.1089/tmj.2018.0124.

-----------------------

Rotenstein LS, Holmgren AJ, Healey MJ, Horn DM, Ting DY, Lipsitz S,et al. [Association between electronic health record time and quality of care metrics in primary care.](https://pubmed-ncbi-nlm-nih-gov.proxy.kib.ki.se/36255725/) JAMA Netw Open. 2022;5(10):e2237086. doi: 10.1001/jamanetworkopen.2022.37086.

--------------------------

Shah HP, Salehi PP, Ihnat J, Kim DD, Salehi P, Judson BL, et al. [Resident burnout and well-being in otolaryngology and other surgical specialties: Strategies for change.](https://pubmed-ncbi-nlm-nih-gov.proxy.kib.ki.se/35133919/) Otolaryngol Head Neck Surg. 2023;168(2):165-79. doi: 10.1177/01945998221076482.

--------------------------

Shanafelt TD, Wang H, Leonard M, Hawn M, McKenna Q, Majzun R, et al.  [Assessment of the association of leadership behaviors of supervising physicians with personal-organizational values alignment among staff physicians.](https://pubmed-ncbi-nlm-nih-gov.proxy.kib.ki.se/33560424/) JAMA Netw Open. 2021;4(2):e2035622. doi: 10.1001/jamanetworkopen.2020.35622.

--------------------------

Shoureshi P, Guerre M, Seideman CA, Callejas DG, Amling CL, Bassale S, et al.  [Addressing Burnout in urology: A qualitative assessment of interventions.](https://pubmed-ncbi-nlm-nih-gov.proxy.kib.ki.se/37145567/)

Urol Pract. 2022;9(1):101-7. doi: 10.1097/UPJ.0000000000000282.

--------------------------

Sinha A, Shanafelt TD, Trockel M, Wang H, Sharp C. [Novel nonproprietary measures of ambulatory electronic health record use associated with physician work exhaustion.](https://pubmed-ncbi-nlm-nih-gov.proxy.kib.ki.se/34261173/) Appl Clin Inform. 2021;12(3):637-46. doi: 10.1055/s-0041-1731678.

--------------------------

Tawfik DS, Shanafelt TD, Dyrbye LN, Sinsky CA, West CP, Davis AS,et al. [Personal and professional factors associated with work-life integration among us physicians.](https://pubmed-ncbi-nlm-nih-gov.proxy.kib.ki.se/34042994/) JAMA Netw Open. 2021;4(5):e2111575. doi: 10.1001/jamanetworkopen.2021.11575.

--------------------------------

Tang K, Labagnara K, Babar M, Loloi J, Watts KL, Jariwala S, et al.  [Electronic health record usage patterns across surgical subspecialties.](https://pubmed-ncbi-nlm-nih-gov.proxy.kib.ki.se/37852294/) Appl Clin Inform. 2024 Jan;15(1):34-44. doi: 10.1055/a-2194-1061.

--------------------------

Yesantharao LV, Joo H, Wei EX, Lin SY, Vohra V, Agrawal Y, et al.  [Factors related to wellness and burnout in academic otolaryngology: A pre- and Post-COVID-19 analysis.](https://pubmed-ncbi-nlm-nih-gov.proxy.kib.ki.se/37090875/) Laryngoscope Investig Otolaryngol. 2023;8(2):409-16. doi: 10.1002/lio2.1033.

--------------------------

Yuan JH, Huang Y, Rosgen BK, Donnelly S, Lan X, Katz SJ. [Burnout and fatigue amongst internal medicine residents: A cross-sectional study on the impact of alternative scheduling models on resident wellness.](https://pubmed-ncbi-nlm-nih-gov.proxy.kib.ki.se/37708198/) PLoS One. 2023;18(9):e0291457. doi: 10.1371/journal.pone.0291457. eCollection 2023.

----------------------------------------------------------------------------------------------------------------------------------------------------------------------------------------------------------------

----------------------------------------------------------------------------------------------------------------------------------------------------------------------------------------------------------------

**Insufficient work-free, paid vacation** (GH6)

Search strategy: (vacation or paid vacation) AND (Physician or Health care professional) AND (burnout)

--------------------------

Al-Sareai NS, Al-Khaldi YM, Mostafa OA, Abdel-Fattah MM.  [Magnitude and risk factors for burnout among primary health care physicians in Asir Province, Saudi Arabia.](https://pubmed-ncbi-nlm-nih-gov.proxy.kib.ki.se/24617120/) East Mediterr Health J. 2013;19(5):426-34.

--------------------------

Anderson JC, Bilal M, Burke CA, Gaidos JK, Lopez R, Oxentenko AS, et al.  [Burnout among US gastroenterologists and fellows in training: Identifying contributing factors and offering solutions.](https://pubmed-ncbi-nlm-nih-gov.proxy.kib.ki.se/36477385/) J Clin Gastroenterol. 2023;57(10):1063-69. doi: 10.1097/MCG.0000000000001781.

--------------------------

Banerjee S, Califano R, Corral J, de Azambuja E, De Mattos-Arruda L, Guarneri V, et al. [Professional burnout in European young oncologists: results of the European Society for Medical Oncology (ESMO) Young Oncologists Committee Burnout Survey.](https://pubmed-ncbi-nlm-nih-gov.proxy.kib.ki.se/28449049/) Ann Oncol. 2017;28(7):1590-6. doi: 10.1093/annonc/mdx196.

--------------------------

Belkić K, Rustagi N. Job stressors in relation to burnout and compromised sleep among academic physicians in India. Work. 2024; 78: 505–525.

-------------------------

Berger B, Cungi PJ, Arzalier S, Lieutaud T, Velly L, Simeone P, et al. [Incidence of burnout syndrome among anesthesiologists and intensivists in France: The REPAR Study.](https://pubmed-ncbi-nlm-nih-gov.proxy.kib.ki.se/36767139/) Int J Environ Res Public Health. 2023;20(3):1771. doi: 10.3390/ijerph20031771.

-------------------------

Brajcich BC, Chung JW, Wood DE, Horvath KD, Tolley PD, Yates EF, et al. [National evaluation of the association between resident labor union participation and surgical resident well-being.](https://pubmed-ncbi-nlm-nih-gov.proxy.kib.ki.se/34468754/) JAMA Netw Open. 2021;4(9):e2123412. doi: 10.1001/jamanetworkopen.2021.23412.

-----------------

Brown CVR, Joseph BA, Davis K, Jurkovich GJ. Modifiable factors to improve work-life balance for trauma surgeons. J Trauma Acute Care Surg. 2021;90(1):122-8. doi: 10.1097/TA.0000000000002910.

-----------------

Cankurtaran CZ, Reddy S, Cen SY, Lei X, Walker DK. [Work-Life experience of academic radiologists: Food for thought.](https://pubmed-ncbi-nlm-nih-gov.proxy.kib.ki.se/36775667/) Acad Radiol. 2023;30(4):579-84. doi: 10.1016/j.acra.2023.01.011.

-----------------

Christie C, Bidwell S, Copeland A, Hudson B. Self-care of Canterbury general practitioners, nurse practitioners, practice nurses and community pharmacists. J Prim Health Care. 2017; 9(4): 286-91. doi: 10.1071/HC17034.

------------------------------

Dietrich LG, Vögelin E, Deml MJ, Pastor T, Gueorguiev B, Pastor T. [Quality of life and working conditions of hand surgeons-A National Survey.](https://pubmed-ncbi-nlm-nih-gov.proxy.kib.ki.se/37629740/)

Medicina (Kaunas). 2023;59(8):1450. doi: 10.3390/medicina59081450.

------------------------------

Doctors Company. [Stressed out during the holidays? Try these tips to avoid burnout.](https://pubmed-ncbi-nlm-nih-gov.proxy.kib.ki.se/23362683/) Mich Med. 2012;111(6):7.

-----------------

Dodds DW, Cruz OA, Israel H. [Attitudes toward retirement of ophthalmology department chairs.](https://pubmed-ncbi-nlm-nih-gov.proxy.kib.ki.se/23531351/) Ophthalmology. 2013;120(7):1502-5. doi: 10.1016/j.ophtha.2012.12.023. Epub 2013 Mar 24.

-----------------

Embriaco N, Papazian L, Kentish-Barnes N, Pochard F, Azoulay E. Burnout syndrome among critical care healthcare workers. Curr Opin Crit Care. 2007; 13: 482–8.

----------------------------------------------

Foote DC, Donkersloot JN, Sandhu G, Ziegler K, Lau J. [Identifying institutional factors in general surgery resident wellness and burnout.](https://pubmed-ncbi-nlm-nih-gov.proxy.kib.ki.se/34332743/) Am J Surg. 2022;223(1):53-7. doi: 10.1016/j.amjsurg.2021.07.014.

----------------------------------------------

Goyal P, Rustagi N, Belkić K. [Physicians' total burden of occupational stressors: More than threefold increased odds of burnout.](https://pubmed-ncbi-nlm-nih-gov.proxy.kib.ki.se/34215893/) South Med J. 2021;114(7):409-15. doi: 10.14423/SMJ.0000000000001277.

----------------------------------------------

Halbach SM, Pillutla K, Seo-Mayer P, Schwartz A, Weidemann D, Mahan JD. [Burnout in pediatric nephrology fellows and faculty: Lessons from the Sustainable Pediatric Nephrology Workforce Project (SUPERPOWER).](https://pubmed-ncbi-nlm-nih-gov.proxy.kib.ki.se/35601419/) Front Pediatr. 2022;10:849370. doi: 10.3389/fped.2022.849370. eCollection 2022.

----------------------------------------------

Hays R, Wynd S, Veitch C, Crossland L. [Getting the balance right? GPs who chose to stay in rural practice.](https://pubmed-ncbi-nlm-nih-gov.proxy.kib.ki.se/14641232/) Aust J Rural Health. 2003;11(4):193-8.

----------------------------------------------

Hedden L, Banihosseini S, Strydom N, McCracken R. [Family physician perspectives on primary care reform priorities: a cross-sectional survey.](https://pubmed-ncbi-nlm-nih-gov.proxy.kib.ki.se/33958382/) CMAJ Open. 2021;9(2):E466-E473. doi: 10.9778/cmajo.20200102.

----------------------------------

Jiménez-Labaig P, Pacheco-Barcia V, Cebrià A, Gálvez F, Obispo B, Páez D, et al. Identifying and preventing burnout in young oncologists, an overwhelming challenge in the COVID-19 era: A study of the Spanish Society of Medical Oncology (SEOM). ESMO Open. 2021;6(4):100215. doi: 10.1016/j.esmoop.2021.100215.

----------------------------------

Kamal AH, Bull JH, Wolf SP, Swetz KM, Shanafelt TD, Ast K, et al. [Prevalence and predictors of burnout among hospice and palliative care clinicians in the U.S.](https://pubmed-ncbi-nlm-nih-gov.proxy.kib.ki.se/26620234/) J Pain Symptom Manage. 2016;51(4):690-96. doi: 10.1016/j.jpainsymman.2015.10.020.

----------------------------------------------

Knight-Davis DK, Fouweather MG, Srivastava ED, Allison MC. [Cross cover for physicians: an additional burden.](https://pubmed-ncbi-nlm-nih-gov.proxy.kib.ki.se/9597628/) J R Coll Physicians Lond. 1998;32(2):130-2.

----------------------------------------------

Kosan Z, Calikoglu EO, Guraksin A. [Levels of burnout and their associated factors among physicians working in Northeast Anatolia.](https://pubmed-ncbi-nlm-nih-gov.proxy.kib.ki.se/29984719/) Niger J Clin Pract. 2018;21(7):875-81. doi: 10.4103/njcp.njcp_298_17.

----------------------------------------------

Khursheed T, Sharif M, Khan MS, Masood A, Aziz W, Shah S, et al. [Burnout in South Asian rheumatologists in the COVID-19 pandemic: an online survey.](https://pubmed-ncbi-nlm-nih-gov.proxy.kib.ki.se/36917244/) Rheumatol Int. 2023;43(6):1143-50. doi: 10.1007/s00296-023-05304-7.

----------------------------------------------

Leep Hunderfund AN, West CP, Rackley SJ, Dozois EJ, Moeschler SM, Vaa Stelling BE, et al. [Social support, social isolation, and burnout: cross-sectional study of U.S. residents exploring associations with individual, interpersonal, program, and work-related factors.](https://pubmed-ncbi-nlm-nih-gov.proxy.kib.ki.se/35442910/) Acad Med. 2022;97(8):1184-94. doi: 10.1097/ACM.0000000000004709.

----------------------------------------------

[Le Gall](https://pubmed-ncbi-nlm-nih-gov.proxy.kib.ki.se/?term=Le+Gall+JR&cauthor_id=22096877) JR, [Azoulay](https://pubmed-ncbi-nlm-nih-gov.proxy.kib.ki.se/?term=Azoulay+E&cauthor_id=22096877) E, [Embriaco](https://pubmed-ncbi-nlm-nih-gov.proxy.kib.ki.se/?term=Embriaco+N&cauthor_id=22096877) N, [Poncet](https://pubmed-ncbi-nlm-nih-gov.proxy.kib.ki.se/?term=Poncet+MC&cauthor_id=22096877) MC, [Pochard](https://pubmed-ncbi-nlm-nih-gov.proxy.kib.ki.se/?term=Pochard+F&cauthor_id=22096877) F. Burn out syndrome among critical care workers. Bull Acad Natl Med. 2011; 195(2):389-97. French.

----------------------------------------------

Lloyd S, Streiner D, Shannon S.  [Burnout, depression, life and job satisfaction among Canadian emergency physicians.](https://pubmed-ncbi-nlm-nih-gov.proxy.kib.ki.se/7963406/) J Emerg Med. 1994;12(4):559-65.**)** doi: 10.1016/0736-4679(94)90360-3.

-------------------------------------

Macía-Rodríguez C, Alejandre de Oña Á, Martín-Iglesias D, Barrera-López L, Pérez-Sanz MT, Moreno-Diaz J, et al. [Burn-out syndrome in Spanish internists during the COVID-19 outbreak and associated factors: a cross-sectional survey.](https://pubmed-ncbi-nlm-nih-gov.proxy.kib.ki.se/33574150/) BMJ Open. 2021;11(2):e042966. doi: 10.1136/bmjopen-2020-042966.

-------------------------------------

[Makara-Studzińska M, Załuski M, Tylec A, Panasiuk L.Do Polish doctors suffer from occupational burnout syndrome? An attempt to find an answer - Pilot study.](https://pubmed-ncbi-nlm-nih-gov.proxy.kib.ki.se/30922052/) Ann Agric Environ Med. 2019;26(1):191-7. doi: 10.26444/aaem/105392.

-------------------------------------

Margolin EJ, Kosber RL, Smigelski MB, Rawjani S, Deleon S, Velji S, et al. [Promoting organizational change: A urology department-wide wellness program to reduce burnout.](https://pubmed-ncbi-nlm-nih-gov.proxy.kib.ki.se/37145807/) Urol Pract. 2022;9(6):615-21. doi: 10.1097/UPJ.0000000000000348.

-------------------------------------

Marshall AL, Elafros M, Duma N. [Work patterns of women physicians during vacation: A cross-sectional study.](https://pubmed-ncbi-nlm-nih-gov.proxy.kib.ki.se/34166095/) J Womens Health (Larchmt). 2022;31(4):573-9. **I** doi: 10.1089/jwh.2021.0100.

----------------------------------------------

Micek MA, Arndt B, Tuan WJ, Trowbridge B, Dean SM, Lochner J, et al. Physician burnout and timing of electronic health record use. ACI open. 2020;4(1):e1-e8. doi: 10.1055/s-0039-3401815.

----------------------------------------------

Milliken A. [Time to breathe.](https://pubmed-ncbi-nlm-nih-gov.proxy.kib.ki.se/24821244/) Hastings Cent Rep. 2014;44(3):8-9. doi: 10.1002/hast.306.

----------------------------------------------

Nahai F. [The art and science of vacationing.](https://pubmed-ncbi-nlm-nih-gov.proxy.kib.ki.se/29534159/) Aesthet Surg J. 2018;38(8):920-22. doi: 10.1093/asj/sjy067.

----------------------------------------------

Nam CS, Daignault-Newton S, Herrel LA, Kraft KH. [Can you have it all? Parenting in urology and work-life balance satisfaction.](https://pubmed-ncbi-nlm-nih-gov.proxy.kib.ki.se/36796543/) Urology. 2023;175:77-83. doi: 10.1016/j.urology.2022.12.044.

----------------------------------------------

Neilson S. Diary of an ICU slave.CMAJ. 2003; 169(12)1315-6.

----------------------------------------------

Nitzsche A, Neumann M, Groß SE, Ansmann L, Pfaff H, Baumann W, et al. [Recovery opportunities, work-home conflict, and emotional exhaustion among hematologists and oncologists in private practice.](https://pubmed-ncbi-nlm-nih-gov.proxy.kib.ki.se/27652494/) Psychol Health Med. 2017;22(4):462-73. doi: 10.1080/13548506.2016.1237666.

-----------------

O'Brien DC, Carr MM. [Current wellness practices among otolaryngology residencies.](https://pubmed-ncbi-nlm-nih-gov.proxy.kib.ki.se/29920216/) Otolaryngol Head Neck Surg. 2018;159(2):258-65. doi: 10.1177/0194599818782408.

-----------------

Ozyurt O, Hayran O, Sur H. Predictors of burnout and job satisfaction among Turkish physicians. QJM. 2006; 99(3):161–9. doi:10.1093/qjmed/hcl019

-----------------

Pang Y, He Y, Chen Z, Han X, Leng J, Tang L. The perceptions of burnout and related influencing factors in Chinese physicians and nurses working in a cancer hospital. Psychooncology. 2021;30(9):1525‐34. doi:10.1002/pon.5709

---------------------

Pipas CF. [Improving physician well-being through organizational change.](https://pubmed-ncbi-nlm-nih-gov.proxy.kib.ki.se/33169959/) Fam Pract Manag. 2020;27(6):23-8.

---------------------

Porto GG, Carneiro SC, Vasconcelos BC, Nascimento MM, Leal JL. [Burnout syndrome in oral and maxillofacial surgeons: a critical analysis.](https://pubmed-ncbi-nlm-nih-gov.proxy.kib.ki.se/24630070/) Int J Oral Maxillofac Surg. 2014;43(7):894-9 doi: 10.1016/j.ijom.2013.10.025.

----------------------

Raphael MJ, Fundytus A, Hopman WM, Vanderpuye V, Seruga B, Lopes G,et al. [Medical oncology job satisfaction: Results of a global survey.](https://pubmed-ncbi-nlm-nih-gov.proxy.kib.ki.se/30685074/) Semin Oncol. 2019;46(1):73-82. doi: 10.1053/j.seminoncol.2018.12.006.

----------------------

Ros SP, Scheper R. [Career longevity in clinical pediatric emergency medicine.](https://pubmed-ncbi-nlm-nih-gov.proxy.kib.ki.se/19633591/) Pediatr Emerg Care. 2009;25(8):487-8. doi: 10.1097/PEC.0b013e3181b09d19.

----------------------

Sammer MBK, Stahl A, Ozkan E, Sher AC.[Implementation of a software distribution intervention to improve workload balance in an academic pediatric radiology department.](https://pubmed-ncbi-nlm-nih-gov.proxy.kib.ki.se/33835322/) J Digit Imaging. 2021;34(3):741-749. doi: 10.1007/s10278-021-00451-4.

----------------------

Shbeer A, Ageel M. [Assessment of occupational burnout among intensive care unit staff in Jazan, Saudi Arabia, using the Maslach Burnout Inventory.](https://pubmed-ncbi-nlm-nih-gov.proxy.kib.ki.se/35469166/) Crit Care Res Pract. 2022;2022:1298887. doi: 10.1155/2022/1298887.

----------------------

Sinsky CA, Trockel MT, Dyrbye LN, Wang H, Carlasare LE, West CP, et al. [Vacation days taken, work during vacation, and burnout among US physicians.](https://pubmed-ncbi-nlm-nih-gov.proxy.kib.ki.se/38214928/) JAMA Netw Open. 2024;7(1):e2351635. doi: 10.1001/jamanetworkopen.2023.51635.

----------------------

Slostad J, Jain S, McKinnon M, Chokkara S, Laiteerapong N. [Evaluation of faculty parental leave policies at medical schools ranked by US News & World Report in 2020.](https://pubmed-ncbi-nlm-nih-gov.proxy.kib.ki.se/36689228/) JAMA Netw Open. 2023;6(1):e2250954. doi: 10.1001/jamanetworkopen.2022.50954.

---------------------------

Smith RP, Rayburn WF. [Burnout in obstetricians-gynecologists: Its prevalence, identification, prevention, and reversal.](https://pubmed-ncbi-nlm-nih-gov.proxy.kib.ki.se/33573788/) Obstet Gynecol Clin North Am. 2021;48(1):231-45. doi: 10.1016/j.ogc.2020.11.008.

---------------------------

Stelfox HT, Straus SE, Sackett DL. Clinician-trialist rounds: 27. Sabbaticals. Part 2: I'm taking a sabbatical! How should I prepare for it?Clin Trials. 2015;12(3):287-90. doi: 10.1177/1740774514567970.

---------------------------

Straus SE, Sackett DL. [Clinician-trialist rounds: 26. Sabbaticals. Part 1: should I take a sabbatical?](https://pubmed-ncbi-nlm-nih-gov.proxy.kib.ki.se/25480539/) Clin Trials. 2015;12(2):174-6. doi: 10.1177/1740774514562917.

--------------------------

Tobin S, Maskrey N. [Ten Commandments for the resilient practitioner.](https://pubmed-ncbi-nlm-nih-gov.proxy.kib.ki.se/27688508/) Br J Gen Pract. 2016;66(651):528-9. doi: 10.3399/bjgp16X687385.

---------------------------

Wickemeyer J, Achim V, Redleaf M. [Stapedectomy is rewarding: But how to prove it?](https://pubmed-ncbi-nlm-nih-gov.proxy.kib.ki.se/31081345/) Ann Otol Rhinol Laryngol. 201;128(10):911-4. doi: 10.1177/0003489419849615.

------------------------

Whippen DA, Canellos GP. [Burnout syndrome in the practice of oncology: results of a random survey of 1,000 oncologists.](https://pubmed-ncbi-nlm-nih-gov.proxy.kib.ki.se/1919641/) J Clin Oncol. 1991;9(10):1916-20 doi: 10.1200/JCO.1991.9.10.1916.

----------------------------------------------------------------------------------------------------------------------------------------------------------------------------------------------------------------

----------------------------------------------------------------------------------------------------------------------------------------------------------------------------------------------------------------

**Insufficient rest breaks**

Search strategy: (rest breaks) AND (Physician or Health care professional) AND (burnout)

--------------------------

Ali NA, Hammersley J, Hoffmann SP, O'Brien JM Jr, Phillips GS, Rashkin M, Warren E, Garland A; Midwest Critical Care Consortium. [Continuity of care in intensive care units: a cluster-randomized trial of intensivist staffing.](https://pubmed-ncbi-nlm-nih-gov.proxy.kib.ki.se/21719756/) Am J Respir Crit Care Med. 2011;184(7):803-8. doi: 10.1164/rccm.201103-0555OC.

---------------------------------------

Belkić K, Savić C. Job stressors and mental health: A proactive clinical perspective. Toh Tuck (Singapore): World Scientific; 2013.

---------------------------------------

Belkić K, Nedić O. Physician health challenges and return to work—insights from participatory action research for physicians by physicians. MedPregl. 2019; 72(11-12): 367–373.

---------------------------------------

Biddle GJ, Thomas N, Edwardson CL, Clemes SA, Daley AJ. [Burnout, psychological wellbeing, and musculoskeletal complaints in UK GPs: an observational study.](https://pubmed-ncbi-nlm-nih-gov.proxy.kib.ki.se/37474254/) BJGP Open. 2023;7(4):BJGPO.2023.0007. doi: 10.3399/BJGPO.2023.0007.

---------------------------------------

Bosch SJ, Valipoor S, Alakshendra A, De Portu G, Mohammadigorji S, Rittenbacher D, et al. [Coping and caregiving: Leveraging environmental design to moderate stress among healthcare workers in the emergency department setting.](https://pubmed-ncbi-nlm-nih-gov.proxy.kib.ki.se/36727220/) HERD. 2023;16(3):261-77. doi: 10.1177/19375867231151243.

---------------------------------------

Durand AC, Bompard C, Sportiello J, Michelet P, Gentile S. Stress and burnout among professionals working in the emergency department in a French university hospital: Prevalence and associated factors. Work. 2019; 63(1):57-67.

---------------------------------------

Freestone MC, Wilson K, Jones R, Mikton C, Milsom S, Sonigra K, et al. [The impact on staff of working with personality disordered offenders: A systematic review.](https://pubmed-ncbi-nlm-nih-gov.proxy.kib.ki.se/26305891/) PLoS One. 2015;10(8):e0136378. doi: 10.1371/journal.pone.0136378.  ---------------------------------------

Gemine R, Davies GR, Tarrant S, Davies RM, James M, Lewis K. Factors associated with work‐related burnout in NHS staff during COVID‐19: a cross‐sectional mixed methods study. BMJ Open. 2021;11(1):e042591.

---------------------------------------

Hall LH, Johnson J, Heyhoe J, Watt I, Anderson K, O'Connor DB. Strategies to improve general practitioner well-being: Findings from a focus group study. Fam Pract. 2018;35(4):511-516doi: 10.1093/fampra/cmx130.

---------------------------------------

Hutter MM, Kellogg KC, Ferguson CM, Abbott WM, Warshaw AL. The impact of the 80-hour resident workweek on surgical residents and attending surgeons. Ann Surg. 2006;243(6):864–71.

---------------------------------------

Ireland MJ, Clough B, Gill K, Langan F, O'Connor A, Spencer L. [A randomized controlled trial of mindfulness to reduce stress and burnout among intern medical practitioners.](https://pubmed-ncbi-nlm-nih-gov.proxy.kib.ki.se/28379084/) Med Teach. 2017;39(4):409-414. doi: 10.1080/0142159X.2017.1294749

----------------------

McGowan Y, Humphries N, Burke H, Conry M, Morgan K. [Through doctors' eyes: A qualitative study of hospital doctor perspectives on their working conditions.](https://pubmed-ncbi-nlm-nih-gov.proxy.kib.ki.se/23480457/) Br J Health Psychol. 2013;18(4):874-91. doi: 10.1111/bjhp.12037.

----------------------

Nitzsche A, Neumann M, Groß SE, Ansmann L, Pfaff H, Baumann W, et al. [Recovery opportunities, work-home conflict, and emotional exhaustion among hematologists and oncologists in private practice.](https://pubmed-ncbi-nlm-nih-gov.proxy.kib.ki.se/27652494/) Psychol Health Med. 2017;22(4):462-473**.** doi: 10.1080/13548506.2016.1237666.

----------------------

Pipas CF. [Improving physician well-being through organizational change.](https://pubmed-ncbi-nlm-nih-gov.proxy.kib.ki.se/33169959/) Fam Pract Manag. 2020;27(6):23-8.

----------------------

Shea JA, Bellini LM, Dinges DF, Curtis ML, Tao Y, Zhu J, et al. [Impact of protected sleep period for internal medicine interns on overnight call on depression, burnout, and empathy.](https://pubmed-ncbi-nlm-nih-gov.proxy.kib.ki.se/24949128/) J Grad Med Educ. 2014;6(2):256-63.

----------------------------------------------------------------------------------------------------------------------------------------------------------------------------------------------------------------

----------------------------------------------------------------------------------------------------------------------------------------------------------------------------------------------------------------

**D. SALARY, POSSIBILITIES FOR ADVANCEMENT & RECOGNITION**

**Lacks recognition of**  **good work** (GU4)

Search strategy: (recognize good work or recognition) AND (Physician or Health care professional) AND (burnout)

--------------------------

Al Rekabi A, Chen M, Patel N, Morgan R, McCafferty I, Haslam P, et al. [Well-being and burnout amongst interventional radiologists in the United Kingdom.](https://pubmed-ncbi-nlm-nih-gov.proxy.kib.ki.se/37380792/) Cardiovasc Intervent Radiol. 2023; 46(8):1053-1063. doi: 10.1007/s00270-023-03455-5.

--------------------------

Angelopoulou P, Panagopoulou E. [Is wellbeing at work related to professional recognition: A pilot intervention.](https://pubmed-ncbi-nlm-nih-gov.proxy.kib.ki.se/31865759/) Psychol Health Med. 2020;25(8):950-7. doi: 10.1080/13548506.2019.1707239.

----------------------------

Baathe F, Rosta J, Bringedal B, Isaksson K. How do doctors experience the interactions among professional fulfilment, organisational factors and quality of patient care? A qualitative study in a Norwegian hospital. BMJ Open. 2019;9(5):e026971. doi: 10.1136/bmjopen-2018-026971.

--------------------------

Babal JC, Lelkes E, Kloster H, Zwemer E, Lien ER, Sklansky D, et al.  [Pediatric resident well-being: A group concept mapping study.](https://pubmed-ncbi-nlm-nih-gov.proxy.kib.ki.se/38215904/) Acad Pediatr. 2024:S1876-2859(24)00002-0. doi: 10.1016/j.acap.2024.01.004..

--------------------------

Beschoner P, Braun M, Schönfeldt-Lecuona C, Freudenmann RW, von Wietersheim J. [[Gender aspects in female and male physicians : Occupational and psychosocial stress].](https://pubmed-ncbi-nlm-nih-gov.proxy.kib.ki.se/27631321/) Bundesgesundheitsblatt Gesundheitsforschung Gesundheitsschutz. 2016;59(10):1343-50. doi: 10.1007/s00103-016-2431-7.PMID: 27631321 German.

--------------------------

Bhembe LQ, Tsai FJ. [Occupational stress and burnout among health care workers caring for people living with HIV in Eswatini.](https://pubmed-ncbi-nlm-nih-gov.proxy.kib.ki.se/30865060/) J Assoc Nurses AIDS Care. 2019;30(6):639-47. doi: 10.1097/JNC.0000000000000068.

----------------------------

Budziński W, Walkiewicz M, Tartas M. [The system of values and styles of success in the medical career: A longitudinal study.](https://pubmed-ncbi-nlm-nih-gov.proxy.kib.ki.se/30568312/) Int J Occup Med Environ Health. 2018;31(6):823-35. doi: 10.13075/ijomeh.1896.01298.

--------------------------

Cagnazzo C, Filippi R, Zucchetti G, Cenna R, Taverniti C, Guarrera ASE, et al. [Clinical research and burnout syndrome in Italy - only a physicians' affair?](https://pubmed-ncbi-nlm-nih-gov.proxy.kib.ki.se/33712055/) Trials. 2021 Mar 12;22(1):205. doi: 10.1186/s13063-021-05158-z.

--------------------------

Caruso A, Vigna C, Bigazzi V, Sperduti I, Bongiorno L, Allocca A.  [Burnout among physicians and nurses working in oncology.](https://pubmed-ncbi-nlm-nih-gov.proxy.kib.ki.se/22619985/) Med Lav. 2012;103(2):96-105.

--------------------------

Cathelain A, Merlier M, Estrade JP, Duhamel A, Phalippou J, Kerbage Y, et al. [Assessment of the quality of life of gynecologic surgeons: A national survey in France.](https://pubmed-ncbi-nlm-nih-gov.proxy.kib.ki.se/32413525/) J Gynecol Obstet Hum Reprod. 2020;49(8):101791. doi: 10.1016/j.jogoh.2020.101791

--------------------------

Chang J, Saggar V, Cortijo-Brown A, Friedman BW, Jones M, Li-Sauerwine S, et al. [Improving physician well-being and reducing burnout using a peer-to-peer recognition program.](https://pubmed-ncbi-nlm-nih-gov.proxy.kib.ki.se/36994318/) AEM Educ Train. 2023;7(2):e10861. doi: 10.1002/aet2.10861.

--------------------------

Chênevert D, Kilroy S, Johnson K, Fournier PL.  [The determinants of burnout and professional turnover intentions among Canadian physicians: application of the job demands-resources model.](https://pubmed-ncbi-nlm-nih-gov.proxy.kib.ki.se/34544396/) BMC Health Serv Res. 2021;21(1):993. doi: 10.1186/s12913-021-06981-5.

--------------------------

Chua IS, Khinkar RM, Wien M, Kerrissey M, Lipsitz S, Cheung YY, et al. [What went right? A mixed-methods study of positive feedback data in a hospital-wide mortality review survey.](https://pubmed-ncbi-nlm-nih-gov.proxy.kib.ki.se/37725228/) J Gen Intern Med. 2023 Sep 19. doi: 10.1007/s11606-023-08393-z.

--------------------------

D'Abreau JA. [A Charge for resident wellness, resilience, and recognition by faculty.](https://pubmed-ncbi-nlm-nih-gov.proxy.kib.ki.se/30913073/) Acad Med. 2019;94(4):457. doi: 10.1097/ACM.0000000000002585.

--------------------------

Denis MA, Iwaz J, Dumetier F, Poyard-Berger G, Vézina M.  [Screening for psychosocial risks among physicians in a pediatric hospital.](https://pubmed-ncbi-nlm-nih-gov.proxy.kib.ki.se/37777348/) Arch Pediatr. 2023;30(8):530-536. doi: 10.1016/j.arcped.2023.09.004.

--------------------------

Estryn-Behar M, Fry C, Guetarni K, Aune I, Machet G, Doppia MA, et al. [Work week duration, work-family balance and difficulties encountered by female and male physicians: results from the French SESMAT study.](https://pubmed-ncbi-nlm-nih-gov.proxy.kib.ki.se/22112665/) Work. 2011;40 Suppl 1:S83-100. doi: 10.3233/WOR-2011-1270.

------------------------------------------

Frade Mera MJ, Vinagre Gaspar R, Zaragoza García I, Viñas Sánchez S, Antúnez Melero E, Alvarez González S, et al. [[Burnout syndrome in different intensive care units].](https://pubmed-ncbi-nlm-nih-gov.proxy.kib.ki.se/20038381/) Enferm Intensiva. 2009;20(4):131-40. doi: 10.1016/s1130-2399(09)73221-3. Spanish

--------------------------

Galhotra S, Smith RB, Norton T, Mahnert ND.  [The surgical gender gap: The impact of surgeon gender in medicine and gynecologic surgery.](https://pubmed-ncbi-nlm-nih-gov.proxy.kib.ki.se/35895969/)

Curr Opin Obstet Gynecol. 2022;34(4):256-261. doi: 10.1097/GCO.0000000000000788.

--------------------------

García Romera I, Danet Danet A, March Cerdá JC. [Emotional climate in primary health care teams. A qualitative approach].](https://pubmed-ncbi-nlm-nih-gov.proxy.kib.ki.se/20413338/) Rev Calid Asist. 2010;25(4):200-6. doi: 10.1016/j.cali.2010.02.004.

--------------------------

Gilewski T. Encountering grief in patient care.Am Soc Clin Oncol Educ Book. 2012:e81-4. doi: 10.14694/EdBook_AM.2012.32.302.

--------------------------

Glick JH, Mulhern V, Olthoff KM, Ende J. [The academy of master clinicians: Recognition of clinical excellence within an academic medical center.](https://pubmed-ncbi-nlm-nih-gov.proxy.kib.ki.se/28795973/)

Acad Med. 2018;93(2):220-3. doi: 10.1097/ACM.0000000000001858.

--------------------------

Green S, Markaki A, Baird J, Murray P, Edwards R. [Addressing healthcare professional burnout: A quality improvement intervention.](https://pubmed-ncbi-nlm-nih-gov.proxy.kib.ki.se/32584485/) Worldviews Evid Based Nurs. 2020;17(3):213-220. doi: 10.1111/wvn.12450.

--------------------------

Guenette JP, Smith SE. Burnout: Job Resources and Job Demands associated with low personal accomplishment in United States radiology residents. Acad Radiol 2018; 25(6):739–43.

--------------------------

Holmes EG, Connolly A, Putnam KT, Penaskovic KM, Denniston CR, Clark LH, et al.  [Taking care of our own: A multispecialty study of resident and program director perspectives on contributors to burnout and potential interventions.](https://pubmed-ncbi-nlm-nih-gov.proxy.kib.ki.se/27436125/) Acad Psychiatry. 2017;41(2):159-66. doi: 10.1007/s40596-016-0590-3.

-------------------

Hughes D, Hanson MN, Alseidi A, Romanelli J, Vassiliou M, Feldman LS, et al.  [Factors influencing surgeon well-being: Qualitatively exploring the joy of surgery.](https://pubmed-ncbi-nlm-nih-gov.proxy.kib.ki.se/37221414/) Surg Endosc. 2023;37(8):6464-75 doi: 10.1007/s00464-023-10135-5.

--------------------------

Joseph MM, Ahasic AM, Clark J, Templeton K. [State of women in medicine: History, challenges, and the benefits of a diverse workforce.](https://pubmed-ncbi-nlm-nih-gov.proxy.kib.ki.se/34470878/) Pediatrics. 2021;148(Suppl 2):e2021051440C. doi: 10.1542/peds.2021-051440C.

--------------------------

Kushnir T, Cohen AH. [Positive and negative work characteristics associated with burnout among primary care pediatricians.](https://pubmed-ncbi-nlm-nih-gov.proxy.kib.ki.se/19143981/) Pediatr Int. 2008;50(4):546-51. doi: 10.1111/j.1442-200X.2008.02619.x.

--------------------------

Lee YS, Campany M, Fullerton S, Malik R, Dorsey C, Mercado D, et al. [State of gender-based microaggressions among surgeons and development of simulation workshops for addressing microaggressions for surgical trainees and students.](https://pubmed-ncbi-nlm-nih-gov.proxy.kib.ki.se/36963719/) Ann Vasc Surg. 2023;95:285-90. doi: 10.1016/j.avsg.2023.03.001.

--------------------------

Mahant S, Mekky M, Parkin P. [Integrating pediatric hospitalists in the academic health science center: Practice and perceptions in a Canadian center.](https://pubmed-ncbi-nlm-nih-gov.proxy.kib.ki.se/20394029/) J Hosp Med. 2010;5(4):228-33doi: 10.1002/jhm.588.

--------------------------

McGowan Y, Humphries N, Burke H, Conry M, Morgan K. [Through doctors' eyes: A qualitative study of hospital doctor perspectives on their working conditions.](https://pubmed-ncbi-nlm-nih-gov.proxy.kib.ki.se/23480457/) Br J Health Psychol. 2013;18(4):874-91 doi: 10.1111/bjhp.12037.

----------------------

McNicholas F, Sharma S, Oconnor C, Barrett E. [Burnout in consultants in child and adolescent mental health services (CAMHS) in Ireland: A cross-sectional study.](https://pubmed-ncbi-nlm-nih-gov.proxy.kib.ki.se/31959602/) BMJ Open. 2020;10(1):e030354. doi: 10.1136/bmjopen-2019-030354.

--------------------

McNicholas F, Adamis D, Minihan E, Doody N, Gavin B. [Occupational stress in clinical and non-clinical staff in Child and Adolescent Mental Health Services (CAMHS): A cross-sectional study.](https://pubmed-ncbi-nlm-nih-gov.proxy.kib.ki.se/35403597/) Ir J Psychol Med. 2022:1-7.**)** doi: 10.1017/ipm.2022.

--------------------

Mehta AB, Lockhart S, Reed K, Griesmer C, Glasgow RE, Moss M, et al. [Drivers of burnout among critical care providers: A multicenter mixed-methods study.](https://pubmed-ncbi-nlm-nih-gov.proxy.kib.ki.se/34896094/) Chest. 2022;161(5):1263-1274. doi: 10.1016/j.chest.2021.11.034.

--------------------------

Merlier M, Ghesquière L, Huissoud C, Drumez E, Morel O, Garabedian C. [How do French Obstetrician-Gynaecologists perceive their quality of life? A national survey.](https://pubmed-ncbi-nlm-nih-gov.proxy.kib.ki.se/37243999/) Eur J Obstet Gynecol Reprod Biol. 2023 Jul;286:112-7. doi: 10.1016/j.ejogrb.2023.05.010.

--------------------------

Mishra K, Kovoor JG, Gupta AK, Bacchi S, Lai CS, Stain SC, Maddern GJ.  [Evolving challenges of leadership in surgery to improve inclusivity, representation, and well-being.](https://pubmed-ncbi-nlm-nih-gov.proxy.kib.ki.se/37758505/) Br J Surg. 2023;110(12):1723 doi: 10.1093/bjs/znad274.

--------------------------

Oman KM, Moulds R, Usher K. [Professional satisfaction and dissatisfaction among Fiji specialist trainees: What are the implications for preventing migration?](https://pubmed-ncbi-nlm-nih-gov.proxy.kib.ki.se/19690206/)

Qual Health Res. 2009;19(9):1246-58. doi: 10.1177/1049732309344116.

--------------------------

Paiva CE, Martins BP, Paiva BSR. [Doctor, are you healthy? A cross-sectional investigation of oncologist burnout, depression, and anxiety and an investigation of their associated factors.](https://pubmed-ncbi-nlm-nih-gov.proxy.kib.ki.se/30367614/) BMC Cancer. 2018;18(1):1044. doi: 10.1186/s12885-018-4964-7.

--------------------------

Phillips G, Shailin S, Lee D, O'Reilly G, Cameron P. ['You can make change happen': Experiences of emergency medicine leadership in the Pacific.](https://pubmed-ncbi-nlm-nih-gov.proxy.kib.ki.se/34796662/) Emerg Med Australas. 2022;34(3):398-410. doi: 10.1111/1742-6723.13905.

--------------------------

Quirk R, Rodin H, Linzer M.  [Targeting causes of burnout in residency: An innovative approach used at Hennepin healthcare.](https://pubmed-ncbi-nlm-nih-gov.proxy.kib.ki.se/33496434/) Acad Med. 2021;96(5):690-4. doi: 10.1097/ACM.0000000000003940.

--------------------------

Rama-Maceiras P, Parente S, Kranke P.  [Job satisfaction, stress and burnout in anaesthesia: Relevant topics for anaesthesiologists and healthcare managers?](https://pubmed-ncbi-nlm-nih-gov.proxy.kib.ki.se/22472627/)

Eur J Anaesthesiol. 2012;29(7):311-9. doi: 10.1097/EJA.0b013e328352816d.

--------------------------

Renzi C, Di Pietro C, Tabolli S. [Psychiatric morbidity and emotional exhaustion among hospital physicians and nurses: Association with perceived job-related factors.](https://pubmed-ncbi-nlm-nih-gov.proxy.kib.ki.se/22524653/) Arch Environ Occup Health. 2012;67(2):117-23. doi: 10.1080/19338244.2011.578682.

--------------------------

Sermeus W, Aiken LH, Ball J, Bridges J, Bruyneel L, Busse R, et al.  [A workplace organisational intervention to improve hospital nurses' and physicians' mental health: Study protocol for the Magnet4Europe wait list cluster randomised controlled trial.](https://pubmed-ncbi-nlm-nih-gov.proxy.kib.ki.se/35902190/) BMJ Open. 2022;12(7):e059159. doi: 10.1136/bmjopen-2021-059159.

--------------------------

Sibeoni J, Bellon-Champel L, Verneuil L, Siaugues C, Revah-Levy A, Farges O. [Workplace environment around physicians' burnout: A qualitative study in French hospitals.](https://pubmed-ncbi-nlm-nih-gov.proxy.kib.ki.se/34363393/) Scand J Work Environ Health. 2021;47(7):521-30. doi: 10.5271/sjweh.3977.

--------------------------

Sigsbee B, Bernat JL. [Physician burnout: A neurologic crisis.](https://pubmed-ncbi-nlm-nih-gov.proxy.kib.ki.se/25378679/) Neurology. 2014;83(24):2302-6. doi: 10.1212/WNL.0000000000001077.

--------------------------

Smith J, Abouzaid L, Masuhara J, Noormohamed S, Remo N, Straatman L. ["I may be essential but someone has to look after my kids": Women physicians and COVID-19.](https://pubmed-ncbi-nlm-nih-gov.proxy.kib.ki.se/34919212/) Can J Public Health. 2022;113(1):107-16. doi: 10.17269/s41997-021-00595-4.

--------------------------

Sriharan A, Ratnapalan S, Tricco AC, Lupea D, Ayala AP, Pang H, Lee DD. [Occupational stress, burnout, and depression in women in healthcare during COVID-19 Pandemic: Rapid scoping review.](https://pubmed-ncbi-nlm-nih-gov.proxy.kib.ki.se/34816168/) Front Glob Womens Health. 2020;1:596690. doi: 10.3389/fgwh.2020.596690.

--------------------

Stephens EH, Heisler CA, Temkin SM, Miller P. [The current status of women in surgery: How to affect the future.](https://pubmed-ncbi-nlm-nih-gov.proxy.kib.ki.se/32639556/) JAMA Surg. 2020;155(9):876-85. doi: 10.1001/jamasurg.2020.0312.

--------------------------

Stokar YN, Pat-Horenczyk R. [Effects of end-of-life care on medical health professionals: A dialectical approach.](https://pubmed-ncbi-nlm-nih-gov.proxy.kib.ki.se/33781366/) Palliat Support Care. 2022;20(1):76-83. doi: 10.1017/S1478951521000365.

--------------------------

Swetz KM, Harrington SE, Matsuyama RK, Shanafelt TD, Lyckholm LJ. [Strategies for avoiding burnout in hospice and palliative medicine: peer advice for physicians on achieving longevity and fulfillment.](https://pubmed-ncbi-nlm-nih-gov.proxy.kib.ki.se/19622012/) J Palliat Med. 2009;12(9):773-7. doi: 10.1089/jpm.2009.0050.

--------------------------

Van Ham I, Verhoeven AA, Groenier KH, Groothoff JW, De Haan J. [Job satisfaction among general practitioners: A systematic literature review.](https://pubmed-ncbi-nlm-nih-gov.proxy.kib.ki.se/17127604/)

Eur J Gen Pract. 2006;12(4):174-80. doi: 10.1080/13814780600994376.

----------------------

Willems R, Monten C, Portzky G. [Exploring the relative importance of work-organizational burnout risk factors in Belgian residents.](https://pubmed-ncbi-nlm-nih-gov.proxy.kib.ki.se/30244668/)

Med Educ Online. 2018;23(1):1521246. doi: 10.1080/10872981.2018.1521246.

--------------------------

Wong BJ, Nassar AK, Earley M, Chen L, Roman-Micek T, Wald SH, et al. [Perceptions of use of names, recognition of roles, and teamwork after labeling surgical caps.](https://pubmed-ncbi-nlm-nih-gov.proxy.kib.ki.se/37976068/) JAMA Netw Open. 2023;6(11):e2341182 doi: 10.1001/jamanetworkopen.2023.41182. Shanafelt TD, Goldhaber-Fiebert SN.

--------------------------

Xu Y, Deng J, Tan W, Yang W, Deng H.  [Mental health of general practitioners in Chongqing, China during COVID-19: A cross-sectional study.](https://pubmed-ncbi-nlm-nih-gov.proxy.kib.ki.se/38035743/) BMJ Open. 2023;13(11):e068333. doi: 10.1136/bmjopen-2022-068333.

----------------------------------------------------------------------------------------------------------------------------------------------------------------------------------------------------------------

----------------------------------------------------------------------------------------------------------------------------------------------------------------------------------------------------------------

**E. Working conditions**

**Office conditions**

Search strategy: [(crowded office) OR (cramped office) OR (windowless office) OR (uncomfortable office) OR (Office space)] AND (Physician or Health care professional) AND (burnout)

----------------------------

Belkić K, Savić C. Job stressors and mental health: A proactive clinical perspective. Toh Tuck (Singapore): World Scientific; 2013.

---------------------------------------

Belkić K, Nedić O. Physician health challenges and return to work—insights from participatory action research for physicians by physicians. MedPregl. 2019; 72(11-12): 367–373.

---------------------------------------

Berger B, Cungi PJ, Arzalier S, Lieutaud T, Velly L, Simeone P, et al. [Incidence of burnout syndrome among anesthesiologists and intensivists in France: The REPAR Study.](https://pubmed-ncbi-nlm-nih-gov.proxy.kib.ki.se/36767139/) Int J Environ Res Public Health. 2023;20(3):1771.**)** doi: 10.3390/ijerph20031771.

----------------------------

Blanchard J, Messman AM, Bentley SK, Lall MD, Liu YT, Merritt R, et al.  [In their own words: Experiences of emergency health care workers during the COVID-19 pandemic.](https://pubmed-ncbi-nlm-nih-gov.proxy.kib.ki.se/35332615/) Acad Emerg Med. 2022;29(8):974-86. doi: 10.1111/acem.14490.

-----------------------

Blazin LJ, Terao MA, Spraker-Perlman H, Baker JN, McLaughlin Crabtree V, et al. [Never enough time: Mixed methods study identifies drivers of temporal demand that contribute to burnout among physicians who care for pediatric hematology-oncology patients.](https://pubmed-ncbi-nlm-nih-gov.proxy.kib.ki.se/33720755/) JCO Oncol Pract. 2021;17(7):e958-e971. doi: 10.1200/OP.20.00754.

-------------------------------------

Bosch SJ, Valipoor S, Alakshendra A, De Portu G, Mohammadigorji S, Rittenbacher D, et al. [Coping and caregiving: Leveraging environmental design to moderate stress among healthcare workers in the emergency department setting.](https://pubmed-ncbi-nlm-nih-gov.proxy.kib.ki.se/36727220/) HERD. 2023;16(3):261-77. doi: 10.1177/19375867231151243.

---------------------------------------

Crowe RP, Fernandez AR, Pepe PE, Cash RE, Rivard MK, Wronski R, et al.. The association of job demands and resources with burnout among emergency medical services professionals.J Am Coll Emerg Physicians Open. 2020 Jan 27;1(1):6-16. doi: 10.1002/emp2.12014.

----------------------

Dunn TJ, Terao MA, Blazin LJ, Spraker-Perlman H, Baker JN, Mandrell B, et al. [Associations of job demands and patient safety event involvement on burnout among a multidisciplinary group of pediatric hematology/oncology clinicians.](https://pubmed-ncbi-nlm-nih-gov.proxy.kib.ki.se/34227729/) Pediatr Blood Cancer. 2021;68(11):e29214. doi: 10.1002/pbc.29214.

----------------------------

Kassam A, Horton J, Shoimer I, Patten S. [Predictors of well-being in resident physicians: A descriptive and psychometric study.](https://pubmed-ncbi-nlm-nih-gov.proxy.kib.ki.se/26217426/) J Grad Med Educ. 2015;7(1):70-4. doi: 10.4300/JGME-D-14-00022.1.

----------------------

Larsen EP, Hailu T, Sheldon L, Ginader A, Bodo N, Dewane D, Degnan AJ, Finley J, Sze RW.J [Optimizing radiology reading room design: The Eudaimonia Radiology Machine.](https://pubmed-ncbi-nlm-nih-gov.proxy.kib.ki.se/33065075/) Am Coll Radiol. 2021;18(1 Pt A):108-20.

--------------------------

Quirk R, Rodin H, Linzer M.  [Targeting causes of burnout in residency: An innovative approach used at Hennepin healthcare.](https://pubmed-ncbi-nlm-nih-gov.proxy.kib.ki.se/33496434/) Acad Med. 2021;96(5):690-4. doi: 10.1097/ACM.0000000000003940.

--------------------------

Toyoshima M, Takenoshita S, Hasegawa H, Kimura T, Nomura K. E[xperiences of negotiations for improving research environment and burnout among young physician researchers in Japan.](https://pubmed-ncbi-nlm-nih-gov.proxy.kib.ki.se/32698340/) Int J Environ Res Public Health. 2020;17(14):5221. doi: 10.3390/ijerph17145221.

------------------------

Ziabari SMZ, Andalib E, Faghani M, Roodsari NN, Arzhangi N, Khesht-Masjedi MF, et al. [Evidence-based design in the hospital environment: A staff's burnout study in the COVID-19 era.](https://pubmed-ncbi-nlm-nih-gov.proxy.kib.ki.se/36691323/) HERD. 2023;16(2):236-49. doi: 10.1177/19375867221148168.

----------------------------

----------------------------

**Radiation exposure**

Search strategy: (radiation exposure) AND (Physician or Health care professional) AND (burnout)

----------------------------

Advani R, Arjonilla M, Guerson A, Taub E, Monzur F. [Gender-specific attitudes of internal medicine residents toward gastroenterology.](https://pubmed-ncbi-nlm-nih-gov.proxy.kib.ki.se/35596822/). Dig Dis Sci. 2022; 67(11):5044-5052. doi: 10.1007/s10620-022-07541-5.

--------------------------

Edling C. [[Risks in physicians' working environment. From plague and anesthesiologic gases to stress and lack of control].](https://pubmed-ncbi-nlm-nih-gov.proxy.kib.ki.se/12092050/) Lakartidningen. 2002;99(22):2506-9.

--------------------------

Lester JD, Hsu S, Ahmad CS. [Occupational hazards facing orthopedic surgeons.](https://pubmed-ncbi-nlm-nih-gov.proxy.kib.ki.se/22530210/) Am J Orthop (Belle Mead NJ). 2012;41(3):132-9.

--------------------------

Nagarkar P. [Personal safety of the plastic surgeon: Keeping yourself healthy while you work.](https://pubmed-ncbi-nlm-nih-gov.proxy.kib.ki.se/29952903/) Plast Reconstr Surg. 2018;142(1):76e-81e. doi: 10.1097/PRS.0000000000004474.

--------------------------

Ryu RC, Behrens PH, Malik AT, Lester JD, Ahmad CS. [Are we putting ourselves in danger? Occupational hazards and job safety for orthopaedic surgeons.](https://pubmed-ncbi-nlm-nih-gov.proxy.kib.ki.se/33716416/) J Orthop. 2021;24:96-101. doi: 10.1016/j.jor.2021.02.023.

----------------------------

----------------------------

**Listens to emotionally-disturbing accounts**

Search strategy: (Emotionally disturbing) AND (Physician or Health care professional) AND (burnout)

---------------------------

---------------------------

Boegelund Kristensen T, Kelstrup Hallas M, Høgsted R, Groenvold M, Sjøgren P, et al. [Burnout in physicians: A survey of the Danish society for palliative medicine.](https://pubmed-ncbi-nlm-nih-gov.proxy.kib.ki.se/34187876/) Support Palliat Care. 2021:bmjspcare-2021-003237. doi: 10.1136/bmjspcare-2021-003237.

--------------------------------

Borritz M, Rugulies R, Bjorner JB, Villadsen E, Mikkelsen OA, Kristensen TS. [Burnout among employees in human service work: design and baseline findings of the PUMA study.](https://pubmed-ncbi-nlm-nih-gov.proxy.kib.ki.se/16449044/) Scand J Public Health. 2006;34(1):49-58. doi: 10.1080/14034940510032275.

--------------------------------

Bosch SJ, Valipoor S, Alakshendra A, De Portu G, Mohammadigorji S, Rittenbacher D, et al. [Coping and caregiving: Leveraging environmental design to moderate stress among healthcare workers in the emergency department setting.](https://pubmed-ncbi-nlm-nih-gov.proxy.kib.ki.se/36727220/) HERD. 2023;16(3):261-77. doi: 10.1177/19375867231151243.

--------------------

Fattori A, Pedruzzi M, Cantarella C, Bonzini M. [The burden in palliative care assistance: A comparison of psychosocial risks and burnout between inpatient hospice and home care services workers.](https://pubmed-ncbi-nlm-nih-gov.proxy.kib.ki.se/35078551/) Palliat Support Care. 2023;21(1):49-56. doi: 10.1017/S1478951521001887.

--------------------

Fernando BMS, Samaranayake DL.[Burnout among postgraduate doctors in Colombo: Prevalence, associated factors and association with self-reported patient care.](https://pubmed-ncbi-nlm-nih-gov.proxy.kib.ki.se/31619216/) BMC Med Educ. 2019;19(1):373. doi: 10.1186/s12909-019-1810-9.

--------------------------------

Fino E, Daniels J, Micheli G, Gazineo D, Godino L, Imbriaco G, et al. Moral injury in a global health emergency: A validation study of the Italian version of the Moral Injury Events Scale adjusted to the healthcare setting. Eur J Psychotraumatol. 2023; 14(2).2263316 doi.org/10.1080/20008066.2023.2263316

--------------------------------

Fischer L, Dadaczynski K, Rathmann K.Pflege. [Psychosoziale Arbeitsbedingungen und Burnout-Symptome in der stationären somatischen und psychiatrischen Gesundheits- und Krankenpflege.](https://pubmed-ncbi-nlm-nih-gov.proxy.kib.ki.se/32208900/) 2020;33(2):93-104. doi: 10.1024/1012-5302/a000720.

-----------------

Fitzpatrick M, Garsia K, Eyre K, Blackhall CA, Pit S.  [Emotional exhaustion among regional doctors in training and the application of international guidelines on sustainable employability management for organisations.](https://pubmed-ncbi-nlm-nih-gov.proxy.kib.ki.se/32183938/) Aust Health Rev. 2020;44(4):609-17. doi: 10.1071/AH19121.

------------------------------

Funding E, Viftrup DT, Knudsen MB, Haunstrup LM, Tolver A, Clemmensen SN. [Impact of training in serious illness communication and work life balance on physicians' self-efficacy, clinical practice and perception of roles.](https://pubmed-ncbi-nlm-nih-gov.proxy.kib.ki.se/37283658/) Adv Med Educ Pract. 2023;14:547-55. doi: 10.2147/AMEP.S406570.

-----------------

Gilewski T. Encountering grief in patient care.Am Soc Clin Oncol Educ Book. 2012:e81-4. doi: 10.14694/EdBook_AM.2012.32.302.

--------------------------

Hagani N, Yagil D, Cohen M. Burnout among among oncologists and oncology nurses: A systematic review and meta-analysis. Health Psychol 2022; 41(1):53-64.

--------------------------

Hardy P, Costemale-Lacoste JF, Trichard C, Butlen-Ducuing F, Devouge I, Cerboneschi V, et al. Comparison of burnout, anxiety and depressive syndromes in hospital psychiatrists and other physicians: Results from the ESTEM study. Psychiatry Res. 2020;284:112662. doi: 10.1016/j.psychres.2019.112662.

--------------

Lue BH, Chen HJ, Wang CW, Cheng Y, Chen MC.  [Stress, personal characteristics and burnout among first postgraduate year residents: A nationwide study in Taiwan.](https://pubmed-ncbi-nlm-nih-gov.proxy.kib.ki.se/20423259/) Med Teach. 2010;32(5):400-7. doi: 10.3109/01421590903437188.

--------------------

Monette DL, Macias-Konstantopoulos WL, Brown DFM, Raja AS, Takayesu JK. A video-based debriefing program to support emergency medicine clinician well-being during the COVID-19 pandemic. West J Emerg Med. 2020;21(6)88-92. DOI: 10.5811/westjem.2020.8.48579

--------------------

Nedić O, Belkić K, Filipović D, Jocić N, Job stressors among female physicians: Relation to having a clinical diagnosis of hypertension. Int J Occup Environ Med. 2010, 16: 330-340.

--------------------

Parola V, Coelho A, Cardoso D, Sandgren A, Apóstolo J. [Prevalence of burnout in health professionals working in palliative care: A systematic review.](https://pubmed-ncbi-nlm-nih-gov.proxy.kib.ki.se/28708752/) JBI Database System Rev Implement Rep. 2017;15(7):1905-33. doi: 10.11124/JBISRIR-2016-003309.

-------------------

Shanafelt T, Adjei A, Meyskens FL. When your favorite patient relapses: Physician grief and well-being in the practice of oncology. J Clin Oncol. 2003; 21(13):2616-9 DOI: 10.1200/JCO.2003.06.075

-------------------Shanafelt T, West C, Zhao X, Novotny P, Kolars J, Habermann T, et al. Relationship between increased personal well-being and enhanced empathy among internal medicine residents. J Gen Intern Med. 2005; 20(7):559-64. DOI: 10.1111/j.1525-1497.2005.0108.x

-------------------

Soto RG, Rosen GP. Pediatric death: guidelines for the grieving anesthesiologist J Clin Anesth 2003; 15: 275-7.

-------------------Stokar YN, Pat-Horenczyk R. [Effects of end-of-life care on medical health professionals: A dialectical approach.](https://pubmed-ncbi-nlm-nih-gov.proxy.kib.ki.se/33781366/) Palliat Support Care. 2022;20(1):76-83. doi: 10.1017/S1478951521000365.

----------------------------------------------------------------------------------------------------------------------------------------------------------------------------------------------------------------

----------------------------------------------------------------------------------------------------------------------------------------------------------------------------------------------------------------

**F. MISHAPS AT WORK**

**Patient suicide**

Search strategy: (patient suicide) AND (Physician or Health care professional) AND (burnout)

--------------------------

Agrawal A, Gitlin M, Melancon SNT, Booth BI, Ghandhi J, DeBonis K. [Responding to a tragedy: Evaluation of a postvention protocol among adult psychiatry residents.](https://pubmed-ncbi-nlm-nih-gov.proxy.kib.ki.se/33686537/) Acad Psychiatry. 2021;45(3):262-71. doi: 10.1007/s40596-021-01418-x.

----------------------

Belkić K, Savić C. Job stressors and mental health: A proactive clinical perspective. Toh Tuck (Singapore): World Scientific; 2013.

---------------------------------------

Belkić K, Nedić O. Physician health challenges and return to work—insights from participatory action research for physicians by physicians. MedPregl. 2019; 72(11-12): 367–373.

---------------------------------------

Bowers L, Simpson A, Eyres S, Nijman H, Hall C, Grange A, et al. [Serious untoward incidents and their aftermath in acute inpatient psychiatry: the Tompkins Acute Ward study.](https://pubmed-ncbi-nlm-nih-gov.proxy.kib.ki.se/17064318/) Int J Ment Health Nurs. 2006;15(4):226-34. doi: 10.1111/j.1447-0349.2006.00428.x.

----------------------

Dwyer AJ, Morley P, Reid E, Angelatos C. Distressed doctors: A hospital-based support program for poorly performing and "at-risk" junior medical staff. Med J Aust. 2011;194(9):466-9. doi: 10.5694/j.1326-5377.2011.tb03063.x.

----------------------

Erlich MD, Rolin SA, Dixon LB, Adler DA, Oslin DW, Levine B, et al.  [Why we need to enhance suicide postvention: evaluating a survey of psychiatrists' behaviors after the suicide of a patient.](https://pubmed-ncbi-nlm-nih-gov.proxy.kib.ki.se/28590263/) J Nerv Ment Dis. 2017;205(7):507-11. doi: 10.1097/NMD.0000000000000682.

----------------------

Kumar S. [Burnout in psychiatrists.](https://pubmed-ncbi-nlm-nih-gov.proxy.kib.ki.se/18188444/) World Psychiatry. 2007 Oct;6(3):186-9.

----------------------

Price R, Weingartner LA, Brikker E, Shaw MA, Shreffler J, O'Connor SS. [Improving medical student attitudes toward suicide prevention through a patient safety planning clerkship initiative.](https://pubmed-ncbi-nlm-nih-gov.proxy.kib.ki.se/35578094/) Acad Psychiatry. 2022;46(5):616-21. doi: 10.1007/s40596-022-01643-y.

----------------------

Siau CS, Chan CMH, Wee LH, Wahab S, Visvalingam U, Chen WS, et al. [Depression and anxiety predict healthcare workers' understanding of and willingness to help suicide attempt patients.](https://pubmed-ncbi-nlm-nih-gov.proxy.kib.ki.se/34096373/) Omega (Westport). 2023;87(2):469-84.

----------------------

Sun FK, Long A. [A theory to guide families and carers of people who are at risk of suicide.](https://pubmed-ncbi-nlm-nih-gov.proxy.kib.ki.se/18592618/) J Clin Nurs. 2008;17(14):1939-48. doi: 10.1111/j.1365-2702.2007.02230.x.

----------------------

----------------------

**Attempted or completed suicide of person(s) with whom one works**

Search strategy: [(colleague suicide attempt) OR (Coworker suicide)] AND (Physician or Health care professional) AND (burnout)

--------------------

Bartone PT, Bartone JV, Violanti JM, Gileno ZM. [Peer Support Services for Bereaved Survivors: A Systematic Review.](https://web-p-ebscohost-com.proxy.kib.ki.se/ehost/viewarticle/render?data=dGJyMPPp44rp2%2fdV0%2bnjisfk5Ie46bFRr6ixSrOk63nn5Kx95uXxjL6prUqzpbBIsKqeTbiqrlKwp55Zy5zyit%2fk8Xnh6ueH7N%2fiVbCoskq0p7dQspzxgeKzr1CxrbVK4NerSeTZs0WypuJNq9euT%2bKj5FCz2%2bF636jie6%2buvorj2ueLpOLfhuWz44ak2uBV4d%2fsSq6c8nnls79mpNfsVa%2bptku3rrJKr5zkh%2fDj34y73POE6urjkPIA&vid=2&sid=183772ba-1fc5-40d5-a07d-f85ecba2dc18@redis) Omega (Westport).  2019; 80(1): 137-66. doi: 10.1177/0030222817728204

--------------------

Dyrbye L, Jin J, Yu Moutier C, Bucks C. After a Physician Suicide. Respond Compassionately and Effectively as an Organization. American Medical Association; 2021.

--------------------

Ey S, Moffit M, Kinzie JM, Brunett PH. [Feasibility of a comprehensive wellness and suicide prevention program: A decade of caring for physicians in training and practice.](https://pubmed-ncbi-nlm-nih-gov.proxy.kib.ki.se/28018541/) J Grad Med Educ. 2016;8(5):747-53. doi: 10.4300/JGME-D-16-00034.1.

----------------------

Hunt TL, Hooten M. Preventing and responding to physician suicide. Mayo Clin Proc. 2024; ;99(3):359-61. doi.org/10.1016/j.mayocp.2023.11.021

----------------------

Legato MJ. Physician suicide: Unnecessary losses. Gender Med. 2009; 6(1): 247

----------------------

Lynn CW. [When a](https://web-p-ebscohost-com.proxy.kib.ki.se/ehost/viewarticle/render?data=dGJyMPPp44rp2%2fdV0%2bnjisfk5Ie46bFRr6ixSrOk63nn5Kx95uXxjL6prUqzpbBIsKqeTbiqrlKwp55Zy5zyit%2fk8Xnh6ueH7N%2fiVbCoskq0p7dQspzxgeKzr1CxrbVK4NerSeTZs0WypuJNq9euT%2bKj5FCz2%2bF636jie6%2buvorj2ueLpOLfhuWz44ak2uBV4d%2fsSq6c8nnls79mpNfsVa%2bms020r7FJr5zkh%2fDj34y73POE6urjkPIA&vid=2&sid=183772ba-1fc5-40d5-a07d-f85ecba2dc18@redis)**[coworker](https://web-p-ebscohost-com.proxy.kib.ki.se/ehost/viewarticle/render?data=dGJyMPPp44rp2%2fdV0%2bnjisfk5Ie46bFRr6ixSrOk63nn5Kx95uXxjL6prUqzpbBIsKqeTbiqrlKwp55Zy5zyit%2fk8Xnh6ueH7N%2fiVbCoskq0p7dQspzxgeKzr1CxrbVK4NerSeTZs0WypuJNq9euT%2bKj5FCz2%2bF636jie6%2buvorj2ueLpOLfhuWz44ak2uBV4d%2fsSq6c8nnls79mpNfsVa%2bms020r7FJr5zkh%2fDj34y73POE6urjkPIA&vid=2&sid=183772ba-1fc5-40d5-a07d-f85ecba2dc18@redis)**[completes](https://web-p-ebscohost-com.proxy.kib.ki.se/ehost/viewarticle/render?data=dGJyMPPp44rp2%2fdV0%2bnjisfk5Ie46bFRr6ixSrOk63nn5Kx95uXxjL6prUqzpbBIsKqeTbiqrlKwp55Zy5zyit%2fk8Xnh6ueH7N%2fiVbCoskq0p7dQspzxgeKzr1CxrbVK4NerSeTZs0WypuJNq9euT%2bKj5FCz2%2bF636jie6%2buvorj2ueLpOLfhuWz44ak2uBV4d%2fsSq6c8nnls79mpNfsVa%2bms020r7FJr5zkh%2fDj34y73POE6urjkPIA&vid=2&sid=183772ba-1fc5-40d5-a07d-f85ecba2dc18@redis)**[suicide](https://web-p-ebscohost-com.proxy.kib.ki.se/ehost/viewarticle/render?data=dGJyMPPp44rp2%2fdV0%2bnjisfk5Ie46bFRr6ixSrOk63nn5Kx95uXxjL6prUqzpbBIsKqeTbiqrlKwp55Zy5zyit%2fk8Xnh6ueH7N%2fiVbCoskq0p7dQspzxgeKzr1CxrbVK4NerSeTZs0WypuJNq9euT%2bKj5FCz2%2bF636jie6%2buvorj2ueLpOLfhuWz44ak2uBV4d%2fsSq6c8nnls79mpNfsVa%2bms020r7FJr5zkh%2fDj34y73POE6urjkPIA&vid=2&sid=183772ba-1fc5-40d5-a07d-f85ecba2dc18@redis)**[.](https://web-p-ebscohost-com.proxy.kib.ki.se/ehost/viewarticle/render?data=dGJyMPPp44rp2%2fdV0%2bnjisfk5Ie46bFRr6ixSrOk63nn5Kx95uXxjL6prUqzpbBIsKqeTbiqrlKwp55Zy5zyit%2fk8Xnh6ueH7N%2fiVbCoskq0p7dQspzxgeKzr1CxrbVK4NerSeTZs0WypuJNq9euT%2bKj5FCz2%2bF636jie6%2buvorj2ueLpOLfhuWz44ak2uBV4d%2fsSq6c8nnls79mpNfsVa%2bms020r7FJr5zkh%2fDj34y73POE6urjkPIA&vid=2&sid=183772ba-1fc5-40d5-a07d-f85ecba2dc18@redis) AAOHN Journal, 2008; 56(11): 459-69.

----------------------

Nakagawa K, Yellowlees PM. [The physician's physician: The role of the psychiatrist in helping other physicians and promoting wellness.](https://pubmed-ncbi-nlm-nih-gov.proxy.kib.ki.se/31358126/)

Psychiatr Clin North Am. 2019;42(3):473-82. doi: 10.1016/j.psc.2019.05.012.

----------------------

Schernhammer E. Taking their own lives—the high rate of physician suicide. N Engl J Med. 2005;352:2473–6.

----------------------

Zabar S, Hanley K, Horlick M, Cocks P, Altshuler L, Watsula-Morley A, et al.  ["I cannot take this any more!": Preparing interns to identify and help a struggling colleague.](https://pubmed-ncbi-nlm-nih-gov.proxy.kib.ki.se/30993628/) J Gen Intern Med. 2019;34(5):773-7. doi: 10.1007/s11606-019-04886-y. Berman R, Hochberg M, Phillips D, Kalet A, Gillespie C.

----------------------

----------------------

**Official complaint against the physician**

Search strategy: [(official complaint)) OR (malpractice)] AND (Physician or Health care professional) AND (burnout)

-----------------

Adelani MA, Hong Z, Miller AN. [Effect of lawsuits on professional well-being and medical error rates among orthopaedic surgeons.](https://pubmed-ncbi-nlm-nih-gov.proxy.kib.ki.se/37311452/) J Am Acad Orthop Surg. 2023;31(16):893-900. doi: 10.5435/JAAOS-D-23-00174.

-----------------

Akbaba M, Davutoğlu V. [[The doctor in a vise between health and the law: What to do?].](https://pubmed-ncbi-nlm-nih-gov.proxy.kib.ki.se/27774974/) Turk Kardiyol Dern Ars. 2016;44(7):609-16. Turkish doi: 10.5543/tkda.2016.69302.

-----------------

Arnhart K, Privitera MR, Fish E, Young A, Hengerer AS, Chaudhry HJ, et al. [Physician burnout and barriers to care on professional applications.](https://web-p-ebscohost-com.proxy.kib.ki.se/ehost/viewarticle/render?data=dGJyMPPp44rp2%2fdV0%2bnjisfk5Ie46bFRr6ixSrOk63nn5Kx95uXxjL6prUq0pbBIsKqeT7iqsVKvqZ5Zy5zyit%2fk8Xnh6ueH7N%2fiVa%2bnrk2zr7ZRs6yki%2bfau1Dk27dKstziReCosUirquF5saPge7Lcq06x2LVIs6y1UbOrsVjw2%2bKB8Zzqeezdu33snOJ6u9nnhrCmpIzf3btZzJzfhrunsVGwp69Nsaekfu3o63nys%2bSN6uLyffbq&vid=2&sid=8fe924fd-b230-4ca3-bc4f-63b705679553@redis) J Leg Med.  2019; 39(3): 235-46. doi: 10.1080/01947648.2019.1629364.

-----------------

Balch CM, Oreskovich MR, Dyrbye LN, Colaiano JM, Satele DV, Sloan JA, et al. [Personal consequences of malpractice lawsuits on American surgeons.](https://pubmed-ncbi-nlm-nih-gov.proxy.kib.ki.se/21890381/) J Am Coll Surg. 2011;213(5):657-67 doi: 10.1016/j.jamcollsurg.2011.08.005. Shanafelt TD.

-----------------

Berman L, Rialon KL, Mueller CM, Ottosen M, Weintraub A, Coakley B, et al. J [Supporting recovery after adverse events: An essential component of surgeon well-being.](https://pubmed-ncbi-nlm-nih-gov.proxy.kib.ki.se/33454081/) Pediatr Surg. 2021;56(5):833-8. doi: 10.1016/j.jpedsurg.2020.12.031.

-----------------

Bhattacharya K, Bhattacharya N. [Surgeon's guilt after postoperative complication.](https://pubmed-ncbi-nlm-nih-gov.proxy.kib.ki.se/36047357/) Pol Przegl Chir. 2022;94(4):45-8. doi: 10.5604/01.3001.0015.6986.

---------------

Brands WG. [[Complaints by complainers?].](https://pubmed-ncbi-nlm-nih-gov.proxy.kib.ki.se/12092337/) Ned Tijdschr Tandheelkd. 2002;109(6):217-21. Dutch

---------------

Buran F, Altın Z. [Burnout among physicians working in a pandemic hospital during the COVID-19 pandemic.](https://web-p-ebscohost-com.proxy.kib.ki.se/ehost/viewarticle/render?data=dGJyMPPp44rp2%2fdV0%2bnjisfk5Ie46bFRr6ixSrOk63nn5Kx95uXxjL6prUq0pbBIsKqeT7iqsVKvqZ5Zy5zyit%2fk8Xnh6ueH7N%2fiVa%2bnrk2zr7ZRs6yki%2bfau1Dk27dKstziReCosUirquF5saPge7Lcq06x2LVIs6y1UbOrsVjw2%2bKB8Zzqeezdu33snOJ6u9nnhrCmpIzf3btZzJzfhruns0i1p7RRtaqkfu3o63nys%2bSN6uLyffbq&vid=2&sid=8fe924fd-b230-4ca3-bc4f-63b705679553@redis) Leg Med (Tokyo). 2021;51:101881. doi: 10.1016/j.legalmed.2021.101881.

---------------

Chen KY, Yang CM, Lien CH, Chiou HY, Lin MR, Chang HR, Chiu WT. [Burnout, job satisfaction, and medical malpractice among physicians.](https://web-p-ebscohost-com.proxy.kib.ki.se/ehost/viewarticle/render?data=dGJyMPPp44rp2%2fdV0%2bnjisfk5Ie46bFRr6ixSrOk63nn5Kx95uXxjL6prUq0pbBIsKqeT7iqsVKvqZ5Zy5zyit%2fk8Xnh6ueH7N%2fiVa%2bnrk2zr7ZRs6yki%2bfau1Dk27dKstziReCosUirquF5saPge7Lcq06x2LVIs6y1UbOrsVjw2%2bKB8Zzqeezdu33snOJ6u9nnhrCmpIzf3btZzJzfhrunrk%2b3p7BPsqekfu3o63nys%2bSN6uLyffbq&vid=2&sid=8fe924fd-b230-4ca3-bc4f-63b705679553@redis) Int J Med Sci.  2013; 10(11): 1471-8. doi: 10.7150/ijms.6743

----------------------------------------

Correia C, Teixeira R, de Almeida NMP, Morais S, Figueiredo P. [Burnout in gastrenterologists: a national-level analysis.](https://pubmed-ncbi-nlm-nih-gov.proxy.kib.ki.se/34403305/) Scand J Gastroenterol. 2021;56(11):1271-8. doi: 10.1080/00365521.2021.1961308.

---------------------------

Crijns TJ, Kortlever JTP, Guitton TG, Ring D, Barron GC. [Symptoms of burnout among surgeons are correlated with a higher incidence of perceived medical errors.](https://pubmed-ncbi-nlm-nih-gov.proxy.kib.ki.se/33380961/) HSS J. 2020;16(Suppl 2):305-10. doi: 10.1007/s11420-019-09727-6.

---------------------------

Dırvar F, Dırvar SU, Kaygusuz MA, Evren B, Öztürk İ. [Effect of malpractice claims on orthopedic and traumatology physicians in Turkey: A survey study.](https://pubmed-ncbi-nlm-nih-gov.proxy.kib.ki.se/33847581/) Acta Orthop Traumatol Turc. 2021;55(2):171-6. doi: 10.5152/j.aott.2021.20167.

---------------------------

Doehring MC, Strachan CC, Haut L, Heniff M, Crevier K, Crittendon M, et al. [Establishing a novel group-based litigation peer support program to promote wellness for physicians involved in medical malpractice lawsuits.](https://pubmed-ncbi-nlm-nih-gov.proxy.kib.ki.se/38353185/) Clin Pract Cases Emerg Med.  2023; 7(4): 205-9. doi: 10.5811/cpcem.1377.

---------------------------

Drill-Mellum L.Minn Med. [Practicing medicine in an imperfect world. Five truths about preventing or surviving a lawsuit.](https://pubmed-ncbi-nlm-nih-gov.proxy.kib.ki.se/23862368/) 2013;96(6):31-3.

---------------------------

Evans R W, Ghosh K. Sur[vey of headache medicine specialists on career satisfaction and burnout.](https://web-p-ebscohost-com.proxy.kib.ki.se/ehost/viewarticle/render?data=dGJyMPPp44rp2%2fdV0%2bnjisfk5Ie46bFRr6ixSrOk63nn5Kx95uXxjL6prUq0pbBIsKqeT7iqsVKvqZ5Zy5zyit%2fk8Xnh6ueH7N%2fiVa%2bnrk2zr7ZRs6yki%2bfau1Dk27dKstziReCosUirquF5saPge7Lcq06x2LVIs6y1UbOrsVjw2%2bKB8Zzqeezdu33snOJ6u9nnhrCmpIzf3btZzJzfhrunr0mwrq5Ktaqkfu3o63nys%2bSN6uLyffbq&vid=2&sid=8fe924fd-b230-4ca3-bc4f-63b705679553@redis) Headache. 2015; 55(10): 1448-57. doi: 10.1111/head.12708.

------------------------------------

Freeborn DK, Levinson W, Mullooly JP  [Medical malpractice and its consequences: does physician gender play a role?](https://web-p-ebscohost-com.proxy.kib.ki.se/ehost/viewarticle/render?data=dGJyMPPp44rp2%2fdV0%2bnjisfk5Ie46bFRr6ixSrOk63nn5Kx95uXxjL6prUq0pbBIsKqeT7iqsVKvqZ5Zy5zyit%2fk8Xnh6ueH7N%2fiVa%2bnrk2zr7ZRs6yki%2bfau1Dk27dKstziReCosUirquF5saPge7Lcq06x2LVIs6y1UbOrsVjw2%2bKB8Zzqeezdu33snOJ6u9nnhrCmpIzf3btZzJzfhrunrk%2bur7VLtamkfu3o63nys%2bSN6uLyffbq&vid=2&sid=8fe924fd-b230-4ca3-bc4f-63b705679553@redis) Journal of Gender, Culture, & Health. 1999; 4(3): 201-14.

------------------------------------

Fresán A, Yoldi-Negrete M, Robles-García R, Tovilla-Zárate CA, Suárez-Mendoza A. [Professional adversities and protective factors associated with suicidal ideation in Mexican psychiatrists.](https://pubmed-ncbi-nlm-nih-gov.proxy.kib.ki.se/32018070/) Arch Med Res. 2019;50(8):484-9.**)** doi: 10.1016/j.arcmed.2019.11.010

------------------------------------

Goto K, Noda M. [Editorial: grieving over complications associated with neuro-endovascular treatment.](https://pubmed-ncbi-nlm-nih-gov.proxy.kib.ki.se/20663346/) Interv Neuroradiol. 2001 Sep 30;7(3):181-90. doi: 10.1177/159101990100700301.

---------------------------

Guillain A, Moncany AH, Hamel O, Gerson C, Bougeard R, Dran G, et al. [Spine neurosurgeons facing the judicialization of their profession: disenchantment and alteration of daily practice-a qualitative study.](https://pubmed-ncbi-nlm-nih-gov.proxy.kib.ki.se/32221729/) Acta Neurochir (Wien). 2020;162(6):1379-87. doi: 10.1007/s00701-020-04302-z.

---------------------------

Gupta K, Rivadeneira NA, Lisker S, Chahal K, Gross N, Sarkar U. [Multispecialty physician online survey reveals that burnout related to adverse event involvement may be mitigated by peer support.](https://pubmed-ncbi-nlm-nih-gov.proxy.kib.ki.se/35482414/) J Patient Saf. 2022 ;18(6):531-8. doi: 10.1097/PTS.0000000000001008.

-----------------------

Klein J, Grosse Frie K, Blum K, von dem Knesebeck O. [Burnout and perceived quality of care among German clinicians in surgery.](https://pubmed-ncbi-nlm-nih-gov.proxy.kib.ki.se/20935011/) Int J Qual Health Care. 2010;22(6):525-30. doi: 10.1093/intqhc/mzq056. Epub 2010 Oct 8.

-------------------------

Laarman BS, Bouwman RJ, de Veer AJ, Hendriks M, Friele RD. [How do doctors in the Netherlands perceive the impact of disciplinary procedures and disclosure of disciplinary measures on their professional practice, health and career opportunities? A questionnaire among medical doctors who received a disciplinary measure.](https://pubmed-ncbi-nlm-nih-gov.proxy.kib.ki.se/30878977/) BMJ Open. 2019;9(3):e023576. doi: 10.1136/bmjopen-2018-023576.

-------------------------

Marmon LM, Heiss K. [Improving surgeon wellness: The second victim syndrome and quality of care.](https://pubmed-ncbi-nlm-nih-gov.proxy.kib.ki.se/26653167/) Semin Pediatr Surg. 2015;24(6):315-8. doi: 10.1053/j.sempedsurg.2015.08.011.

------------------------

Mazurenko O, Menachemi N. [Environmental market factors associated with physician career satisfaction.](https://pubmed-ncbi-nlm-nih-gov.proxy.kib.ki.se/23087994/) J Healthc Manag. 2012;57(5):307-22;

------------------------

Mull CC, Thompson AD, Selbst SM, Miller EG, Rappaport DI, Gartner JC Jr, Bowman WR.  [A call to restore your calling: self-care of the emergency physician in the face of life-changing stress-Part 1 of 6.](https://web-p-ebscohost-com.proxy.kib.ki.se/ehost/viewarticle/render?data=dGJyMPPp44rp2%2fdV0%2bnjisfk5Ie46bFRr6ixSrOk63nn5Kx95uXxjL6prUq0pbBIsKqeT7iqsVKvqZ5Zy5zyit%2fk8Xnh6ueH7N%2fiVa%2bnrk2zr7ZRs6yki%2bfau1Dk27dKstziReCosUirquF5saPge7Lcq06x2LVIs6y1UbOrsVjw2%2bKB8Zzqeezdu33snOJ6u9nnhrCmpIzf3btZzJzfhrunsU22qrZNtaukfu3o63nys%2bSN6uLyffbq&vid=2&sid=8fe924fd-b230-4ca3-bc4f-63b705679553@redis) Pediatr Emerg Care.  2019; 35(4): 319-22. doi: 10.1097/PEC.0000000000001807

------------------------

Parks-Savage A, Archer L, Newton H, Wheeler E, Huband SR. [Prevention of medical errors and malpractice: Is creating resilience in physicians part of the answer?](https://web-p-ebscohost-com.proxy.kib.ki.se/ehost/viewarticle/render?data=dGJyMPPp44rp2%2fdV0%2bnjisfk5Ie46bFRr6ixSrOk63nn5Kx95uXxjL6prUq0pbBIsKqeT7iqsVKvqZ5Zy5zyit%2fk8Xnh6ueH7N%2fiVa%2bnrk2zr7ZRs6yki%2bfau1Dk27dKstziReCosUirquF5saPge7Lcq06x2LVIs6y1UbOrsVjw2%2bKB8Zzqeezdu33snOJ6u9nnhrCmpIzf3btZzJzfhrunsUm1qa5Orq6kfu3o63nys%2bSN6uLyffbq&vid=2&sid=8fe924fd-b230-4ca3-bc4f-63b705679553@redis) Int J Law Psychiatry. 2018; 60: 35-9. doi: 10.1016/j.ijlp.2018.07.003.

------------------------

Rappaport DI, Selbst SM. [Medical errors and malpractice lawsuits: Impact on providers- Part 2 of 6.](https://pubmed-ncbi-nlm-nih-gov.proxy.kib.ki.se/31135686/) Pediatr Emerg Care. 2019;35(6):440-2. doi: 10.1097/PEC.0000000000001856.

**---------------------------**

Soh IY, Money SR, Huber TS, Coleman DM, Sheahan MG, Morrissey NJ, Hallbeck MS, Meltzer AJ. [Malpractice allegations against vascular surgeons: Prevalence, risk factors, and impact on surgeon wellness.](https://pubmed-ncbi-nlm-nih-gov.proxy.kib.ki.se/34478809/) J Vasc Surg. 2022;75(2):680-686. doi: 10.1016/j.jvs.2021.07.233.

**---------------------------**

# Tan, E C-H; Chen D-R, Second victim: Malpractice disputes and quality of life among primary care physicians J Formos Med Assoc. 2019; 118(2): 619-27 doi: 10.1016/j.jfma.2018.07.012.

-----------------------

Tawfik DS, Profit J, Morgenthaler TI, Satele DV, Sinsky CA, Dyrbye LN, et al. [Physician burnout, well-being, and work unit safety grades in relationship to reported medical errors.](https://pubmed-ncbi-nlm-nih-gov.proxy.kib.ki.se/30001832/) Mayo Clin Proc. 2018 Nov;93(11):1571-1580. doi: 10.1016/j.mayocp.2018.05.014

-----------------------

Tawfik DS, Profit J, Morgenthaler TI, Satele DV, Sinsky C, Dyrbye L, et al.  [In Reply-burnout is not associated with increased medical errors.](https://pubmed-ncbi-nlm-nih-gov.proxy.kib.ki.se/30392548/) Mayo Clin Proc. 2018;93(11):1683-4. doi: 10.1016/j.mayocp.2018.08.014.

-----------------------

Travers V. [Burnout in orthopedic surgeons.](https://pubmed-ncbi-nlm-nih-gov.proxy.kib.ki.se/31740160/) Orthop Traumatol Surg Res. 2020;106(1S):S7-S12. doi: 10.1016/j.otsr.2019.04.029.

-----------------------

Walker AL, Gamble J, Creedy DK, Ellwood DA.  [Impact of traumatic birth on Australian obstetricians: A pilot feasibility study.](https://pubmed-ncbi-nlm-nih-gov.proxy.kib.ki.se/31828770/) Aust N Z J Obstet Gynaecol. 2020;60(4):555-60. doi: 10.1111/ajo.13107.

------------------------

Wu AW. Medical error: the second victim. The doctor who makes the mistake needs help too. BMJ. 2000; 320(7237): 726-7.

----------------------------------------------------------------------------------------------------------------------------------------------------------------------------------------------------------------

----------------------------------------------------------------------------------------------------------------------------------------------------------------------------------------------------------------

**G. TIME PRESSURE AT WORK**

**Time constraints preclude completion of work tasks**

Search strategy: (work load time pressure conflict) AND (Physician or Health care professional) AND (burnout)

----------------------

Banerjee G, Mitchell JD, Brzezinski M, DePorre A, Ballard HA. [Burnout in academic physicians.](https://pubmed-ncbi-nlm-nih-gov.proxy.kib.ki.se/37309180/) Perm J. 2023;27(2):142-9. doi: 10.7812/TPP/23.032.

----------------------------

Boivin J, Bunting L, Koert E, Ieng U C, Verhaak C. [Perceived challenges of working in a fertility clinic: a qualitative analysis of work stressors and difficulties working with patients.](https://web-p-ebscohost-com.proxy.kib.ki.se/ehost/viewarticle/render?data=dGJyMPPp44rp2%2fdV0%2bnjisfk5Ie46bFRr6ixSrOk63nn5Kx95uXxjL6prUq1pbBIsKqeSa%2bwr064p7c4v8OkjPDX7Ivf2fKB7eTnfLuorlGuqLBPsq%2byPvHf4lW0p%2bFOs66yUavZsn2vo7JJr6arerSqs0Wvp7V7strjT7Cv5Ey%2b6ON85%2bmkhN%2fk5VXj5KR84LPhgeyorj7y1%2bVVv8Skeeyzr0qur7ZQsayyPuTl8IXf6rt%2b8%2bLqjOPu8gAA&vid=2&sid=61c65849-c4e1-4110-b645-117c4de729f4@redis) Hum Reprod.  2017; 32(2): 403-8**.** doi: 10.1093/humrep/dew326.

----------------------------

Dominic C, Gopal DP, Sidhu A.  ['It's like juggling fire daily': Well-being, workload and burnout in the British NHS - A survey of 721 physicians.](https://pubmed-ncbi-nlm-nih-gov.proxy.kib.ki.se/34633337/) Work. 2021;70(2):395-403. doi: 10.3233/WOR-205337.

----------------------

Jantea R, Buranosky R, Simak D, Hoffman E, Zimmer SM, Elnicki DM. [The 50/50 block schedule: Impact on residents' and preceptors' perceptions, patient outcomes, and continuity of care.](https://web-p-ebscohost-com.proxy.kib.ki.se/ehost/viewarticle/render?data=dGJyMPPp44rp2%2fdV0%2bnjisfk5Ie46bFRr6ixSrOk63nn5Kx95uXxjL6prUq1pbBIsKqeSa%2bwr064p7c4v8OkjPDX7Ivf2fKB7eTnfLuorlGuqLBPsq%2byPvHf4lW0p%2bFOs66yUavZsn2vo7JJr6arerSqs0Wvp7V7strjT7Cv5Ey%2b6ON85%2bmkhN%2fk5VXj5KR84LPhgeyorj7y1%2bVVv8Skeeyzr0q2rK9Pt6a3PuTl8IXf6rt%2b8%2bLqjOPu8gAA&vid=2&sid=61c65849-c4e1-4110-b645-117c4de729f4@redis) Teach Learn Med. 2018; 30(2): 223-32.

----------------------

Lancman S, Mângia EF, Muramoto MT. [Impact of conflict and violence on workers in a hospital emergency room.](https://web-p-ebscohost-com.proxy.kib.ki.se/ehost/viewarticle/render?data=dGJyMPPp44rp2%2fdV0%2bnjisfk5Ie46bFRr6ixSrOk63nn5Kx95uXxjL6prUq1pbBIsKqeSa%2bwr064p7c4v8OkjPDX7Ivf2fKB7eTnfLuorlGuqLBPsq%2byPvHf4lW0p%2bFOs66yUavZsn2vo7JJr6arerSqs0Wvp7V7strjT7Cv5Ey%2b6ON85%2bmkhN%2fk5VXj5KR84LPhgeyorj7y1%2bVVv8Skeeyzr0iyqK9QtKm1PuTl8IXf6rt%2b8%2bLqjOPu8gAA&vid=2&sid=61c65849-c4e1-4110-b645-117c4de729f4@redis)Work, 2013; 45(4): 519-27.

----------------------------------

Quirk R, Rodin H, Linzer M.  [Targeting causes of burnout in residency: An innovative approach used at Hennepin healthcare.](https://pubmed-ncbi-nlm-nih-gov.proxy.kib.ki.se/33496434/) Acad Med. 2021;96(5):690-4.

----------------------------------------------------------------------------------------------------------------------------------------------------------------------------------------------------------------

----------------------------------------------------------------------------------------------------------------------------------------------------------------------------------------------------------------

**H. PROBLEMS, RESTRICTIONS/CONSTRAINTS**

**Problems/deficiencies hinder patient care** (OCNFL2) **--Understaffing specifically hinders patient care**

Search strategy: [(technical problems) or (Hindrances to patient care) or (Understaffing)] AND (Physician or Health care professional) AND (burnout)

----------------------

Abrams R, Jones B, Campbell J, de Lusignan S, Peckham S, Gage H. [The effect of general practice team composition and climate on staff and patient experiences: A systematic review.](https://pubmed-ncbi-nlm-nih-gov.proxy.kib.ki.se/37827584/) BJGP Open. 2023:BJGPO.2023.0111. doi: 10.3399/BJGPO.2023.0111.

----------------

Antia A, Ndukauba C, Pius R, Gbegbaje O, Ong K. [Impact of day of admission on in-hospital outcomes of cardiogenic shock.](https://pubmed-ncbi-nlm-nih-gov.proxy.kib.ki.se/37858845/) Curr Probl Cardiol. 2024;49(1 Pt C):102140. doi: 10.1016/j.cpcardiol.2023.102140

----------------

Belkić K, Rustagi N. Job stressors in relation to burnout and compromised sleep among academic physicians in India. Work. 2024; 78: 505–525.

----------------

Chambers CN, Frampton CM, Barclay M, McKee M. [Burnout prevalence in New Zealand's public hospital senior medical workforce: A cross-sectional mixed methods study.](https://pubmed-ncbi-nlm-nih-gov.proxy.kib.ki.se/27881531/) BMJ Open. 2016;6(11):e013947. doi: 10.1136/bmjopen-2016-013947.

-------------------

Dillon EC, Tai-Seale M, Meehan A, Martin V, Nordgren R, Lee T, et al. [Frontline perspectives on physician burnout and strategies to improve well-being: Interviews with physicians and health system leaders.](https://pubmed-ncbi-nlm-nih-gov.proxy.kib.ki.se/31659668/) J Gen Intern Med. 2020;35(1):261-7. doi: 10.1007/s11606-019-05381-0.

-------------------

Dominic C, Gopal DP, Sidhu A.  ['It's like juggling fire daily': Well-being, workload and burnout in the British NHS - A survey of 721 physicians.](https://pubmed-ncbi-nlm-nih-gov.proxy.kib.ki.se/34633337/) Work. 2021;70(2):395-403. doi: 10.3233/WOR-205337.

-------------------

Green ME; Van Iersel RI. [Response of rural physicians in a non--fee-for-service environment to acute increases in demand due to physician shortages.](https://web-p-ebscohost-com.proxy.kib.ki.se/ehost/viewarticle/render?data=dGJyMPPp44rp2%2fdV0%2bnjisfk5Ie46bFRr6ixSrOk63nn5Kx95uXxjL6prUqupbBIsKqeT7imtFKxrp5oy5zyit%2fk8Xnh6ueH7N%2fiVbWtr0u1qK5MpOnnfLuq40u3r%2bRMtqPge6%2fcq0yxq%2bBF4Nffe6upr3u2p7JPsa61S%2bC28H3i3%2fE%2b6tfsf7vb7D7i2Lt75%2bSwSKTq33%2b7t8w%2b3%2bS7Sa6rtk%2bvrbJQpNztiuvX8lXk6%2bqE8tv2jAAA&vid=2&sid=4e399f48-bc1f-435b-baac-31c81473873b@redis) Can J Rural Med.2007; 12(1): 10-15.

----------------------

Gregory ST, Menser T, Gregory BT. An organizational intervention to reduce physician burnout. J Healthc Manag 2018;63(5):338–52.

----------------------

Haffizulla F, Newman C, Kaushai S, Williams C, Haffizulla A, Hardigan P, et al. Assessment of burnout: A pilot study of international women physicians. Perm J. 2020; 24:20.028. doi.org/10.7812/TPP/20.028

------------------

Hämmig O. Explaining burnout and the intention to leave the profession among health professionals - a cross-sectional study in a hospital setting in Switzerland.BMC Health Serv Res. 2018;18(1):785. doi: 10.1186/s12913-018-3556-1.

-------------

Ilić IM, Arandjelović MŽ, Jovanović JM, Nešić MM. [Relationships of work-related psychosocial risks, stress, individual factors and burnout - Questionnaire survey among emergency physicians and nurses.](https://pubmed-ncbi-nlm-nih-gov.proxy.kib.ki.se/28345677/) Med Pr. 2017;68(2):167-78. doi: 10.13075/mp.5893.00516.

-------------

Lasater KB, Aiken LH, Sloane DM, French R, Martin B, Reneau K, et al. [Chronic hospital nurse understaffing meets COVID-19: an observational study.](https://pubmed-ncbi-nlm-nih-gov.proxy.kib.ki.se/32817399/) BMJ Qual Saf. 2021;30(8):639-647. doi: 10.1136/bmjqs-2020-011512.

----------------------

McGowan Y, Humphries N, Burke H, Conry M, Morgan K. [Through doctors' eyes: A qualitative study of hospital doctor perspectives on their working conditions.](https://pubmed-ncbi-nlm-nih-gov.proxy.kib.ki.se/23480457/) Br J Health Psychol. 2013;18(4):874-91.**)** doi: 10.1111/bjhp.12037.

----------------------

Nedić O, Belkić K. Job stressors and burnout among nurses and primary-care physicians working at a dedicated outpatient respiratory center for patients with suspected or confirmed COVID-19. Am J Ind Med. 2023; 66 (6): 510-28.

----------------------

Petrino R, Riesgo LG, Yilmaz B.  [Burnout in emergency medicine professionals after 2 years of the COVID-19 pandemic: A threat to the healthcare system?](https://pubmed-ncbi-nlm-nih-gov.proxy.kib.ki.se/35620812/) Eur J Emerg Med. 2022;29(4):279-84. doi: 10.1097/MEJ.0000000000000952.

---------------------

Quirk R, Rodin H, Linzer M.  [Targeting causes of burnout in residency: An innovative approach used at Hennepin healthcare.](https://pubmed-ncbi-nlm-nih-gov.proxy.kib.ki.se/33496434/) Acad Med. 2021;96(5):690-4.

----------------------

Sturm H, Rieger MA, Martus P, Ueding E, Wagner A, Holderried M, et al. [Do perceived working conditions and patient safety culture correlate with objective workload and patient outcomes: A cross-sectional explorative study from a German university hospital.](https://pubmed-ncbi-nlm-nih-gov.proxy.kib.ki.se/30608945/) PLoS One. 2019;14(1):e0209487. doi: 10.1371/journal.pone.0209487.

---------------------------

Walia S, Nordenholz KE, Krywko D, Norvell JG, Hewitt KV, Parmele KT, et al. [The Chief Wellness Officer: A long overdue catalyst for systemic change in Emergency Medicine.](https://pubmed-ncbi-nlm-nih-gov.proxy.kib.ki.se/37823601/) Int J Health Plann Manage. 2024;39(1):141-51. doi: 10.1002/hpm.3714.

---------------------------

Ward NS, Afessa B, Kleinpell R, Tisherman S, Ries M, Howell M, et al. [Intensivist/patient ratios in closed ICUs: a statement from the Society of Critical Care Medicine Taskforce on ICU Staffing.](https://pubmed-ncbi-nlm-nih-gov.proxy.kib.ki.se/23263586/) Crit Care Med. 2013;41(2):638-45. doi: 10.1097/CCM.0b013e3182741478.

--------------

Zhao X, Liu S, Chen Y, Zhang Q, Wang Y.  [Influential factors of burnout among village doctors in China: A cross-sectional study.](https://pubmed-ncbi-nlm-nih-gov.proxy.kib.ki.se/33669640/) Int J Environ Res Public Health. 2021;18(4):2013. doi: 10.3390/ijerph18042013.

--------------------

--------------------

**Interruptions from people hamper task performance** (OCNFL3)

(Interruptions) AND (Physician or Health care professional) AND (burnout)

Bosch SJ, Valipoor S, Alakshendra A, De Portu G, Mohammadigorji S, Rittenbacher D,et al. [Coping and caregiving: Leveraging environmental design to moderate stress among healthcare workers in the emergency department setting.](https://pubmed-ncbi-nlm-nih-gov.proxy.kib.ki.se/36727220/) HERD. 2023;16(3):261-77. doi: 10.1177/19375867231151243

--------------------------------

Carlson KJ, Matthias TH, Birge JR, Bulian BP, Richards SE, Shiffermiller JF. [The effect of geographic rounding on hospitalist work experience: A mixed-methods study.](https://pubmed-ncbi-nlm-nih-gov.proxy.kib.ki.se/35253585/) (1995). Hosp Pract. 2022;50(2):124-31. doi: 10.1080/21548331.2022.2050649.

--------------------------------

Eng MS, Fierro K, Abdouche S, Yu D, Schreyer KE. [Perceived vs. actual distractions in the emergency department.](https://pubmed-ncbi-nlm-nih-gov.proxy.kib.ki.se/30686536/) Am J Emerg Med. 2019;37(10):1896-903. doi: 10.1016/j.ajem.2019.01.005.

--------------------------------

Geva A, Landrigan CP, van der Velden MG, Randolph AG. Simulation of a novel schedule for intensivist staffing to improve continuity of patient care and reduce physician burnout. Crit Care Med. 2017;45(7):1138-1144. doi: 10.1097/CCM.0000000000002319.

-------------------------

Johnson HM, Irish W, Strassle PD, Mahoney ST, Schroen AT, Josef AP, et al. [Associations between career satisfaction, personal life factors, and work-life integration practices among US surgeons by gender.](https://pubmed-ncbi-nlm-nih-gov.proxy.kib.ki.se/32579211/) JAMA Surg. 2020;155(8):742-50. doi: 10.1001/jamasurg.2020.1332. Freischlag JA, Tuttle JE, Brownstein MR.

-------------------------

Kapoor R, Gupta N, Roberts SD, Naum C, Perkins AJ, Khan BA.  [Impact of geographical cohorting in the ICU: An academic tertiary care center experience.](https://pubmed-ncbi-nlm-nih-gov.proxy.kib.ki.se/33063024/) Crit Care Explor. 2020;2(10):e0212. doi: 10.1097/CCE.0000000000000212.

-----------------------------

Lapointe R, Bhesania S, Tanner T, Peruri A, Mehta P. [An innovative approach to improve communication and reduce physician stress and burnout in a university affiliated residency program.](https://pubmed-ncbi-nlm-nih-gov.proxy.kib.ki.se/29808384/) J Med Syst. 2018;42(7):117. doi: 10.1007/s10916-018-0956-z.

---------------------------

Larsen EP, Hailu T, Sheldon L, Ginader A, Bodo N, Dewane D, Degnan AJ, Finley J, Sze RW.J [Optimizing radiology reading room design: The Eudaimonia Radiology Machine.](https://pubmed-ncbi-nlm-nih-gov.proxy.kib.ki.se/33065075/) Am Coll Radiol. 2021;18(1 Pt A):108-20. doi: 10.1016/j.jacr.2020.09.041.

---------------------------

Lee J, Aoude A, Alhalabi B, Watt A, Lessard L M. Can an emergency surgery scheduling software improve residents' time management and quality of life? Journal of Medicine. 2022; 20(2): 10-15.
-----------

Li-Wang J, Townsley A, Katta R.  [Cognitive ergonomics: A review of interventions for outpatient practice.](https://pubmed-ncbi-nlm-nih-gov.proxy.kib.ki.se/37772235/) Cureus. 2023;15(8):e44258. doi: 10.7759/cureus.44258.

-----------

Luu T, Spiegelman L, Nykin D, Abido K, Roh J, Rudkin S, et al. Implementation of an electronic health record-based messaging system in the emergency department: Effects on physician workflow and resident burnout. J Patient Saf. 2022; 18(2):e542-6. doi: 10.1097/PTS.0000000000000869. Toohey S

-----------

Mazur LM, Adapa K, Meltzer-Brody S, Karwowski W.  [Towards better understanding of workplace factors contributing to hospitalist burden and burnout prior to COVID-19 pandemic.](https://pubmed-ncbi-nlm-nih-gov.proxy.kib.ki.se/36030734/) Appl Ergon. 2023;106:103884. doi: 10.1016/j.apergo.2022.103884.

----------------------

McGowan Y, Humphries N, Burke H, Conry M, Morgan K. [Through doctors' eyes: A qualitative study of hospital doctor perspectives on their working conditions.](https://pubmed-ncbi-nlm-nih-gov.proxy.kib.ki.se/23480457/) Br J Health Psychol. 2013;18(4):874-91. doi: 10.1111/bjhp.12037.

----------------------

Nedić O, Belkić K, Filipović D, Jocić N, Job stressors among female physicians: Relation to having a clinical diagnosis of hypertension, Int J Occup Environ Med 2010, 16: 330-340.

----------------------

Rick VB, Brandl C, Mertens A, Nitsch V. Work interruptions of office workers: The influence of the complexity of primary work tasks on the perception of interruptions. Work. 2024; 77(1): 185-96.

----------------------

Rushing CJ, Roland DA, Pham A, Bodden VM, Soldano SA, Epstein S, et al. [A formal work hour analysis of the resident foot and ankle surgeon.](https://pubmed-ncbi-nlm-nih-gov.proxy.kib.ki.se/30583784/) J Foot Ankle Surg. 2019;58(1):80-85. doi: 10.1053/j.jfas.2018.08.005.

----------------------

Santos CN, Pedrosa BF, Martins M, Gouveia F, Franco F, Vardasca MJ, et al. [Interruptions during general practice consultations: negative impact on physicians, and patients' indifference.](https://pubmed-ncbi-nlm-nih-gov.proxy.kib.ki.se/36409278/) Fam Pract. 2022:cmac129.**)** doi: 10.1093/fampra/cmac129.

----------------------

Schwarzkopf D, Westermann I, Skupin H, Riedemann NC, Reinhart K, Pfeifer R, et al. [A novel questionnaire to measure staff perception of end-of-life decision making in the intensive care unit--development and psychometric testing.](https://pubmed-ncbi-nlm-nih-gov.proxy.kib.ki.se/25311265/) .J Crit Care. 2015;30(1):187-95. doi: 10.1016/j.jcrc.2014.09.015.

----------------------

Seelandt JC, Tschan F, Keller S, Beldi G, Jenni N, Kurmann A, Candinas D, Semmer NK. [Assessing distractors and teamwork during surgery: developing an event-based method for direct observation.](https://pubmed-ncbi-nlm-nih-gov.proxy.kib.ki.se/25013008/) BMJ Qual Saf. 2014;23(11):918-29. doi: 10.1136/bmjqs-2014-002860.

------------------------

Shreffler J, Huecker M. [Physician flow at work: examining work absorption, clinical flow, work fulfillment, and flow thieves.](https://pubmed-ncbi-nlm-nih-gov.proxy.kib.ki.se/35766249/) Workplace Health Saf. 2022;70(11):484-491. doi: 10.1177/21650799221093772.

----------------------

Simon AF, Holmes JH, Schwartz ES. [Decreasing radiologist burnout through informatics-based solutions.](https://pubmed-ncbi-nlm-nih-gov.proxy.kib.ki.se/31821974/) Clin Imaging. 2020;59(2):167-171. doi: 10.1016/j.clinimag.2019.10.014.

----------------------

Sinsky CA, Privitera MR. Creating a "Manageable Cockpit" for clinicians: A shared responsibility. JAMA Intern Med. 2018 Jun;178(6):741-2. doi: 10.1001/jamainternmed.2018.0575.

----------------------

Steitz BD, Sulieman L, Warner JL, Fabbri D, Brown JT, Davis AL, et al. [Classification and analysis of asynchronous communication content between care team members involved in breast cancer treatment.](https://pubmed-ncbi-nlm-nih-gov.proxy.kib.ki.se/34396056/) JAMIA Open. 2021;4(3):ooab049. doi: 10.1093/jamiaopen/ooab049.

----------------------

Williams KL, Renouf TS, Dubrowski A. [Pitfalls in emergency medicine: survey-based identification of learning objectives for targeted simulation curricula by emergency department staff.](https://pubmed-ncbi-nlm-nih-gov.proxy.kib.ki.se/33425541/) Cureus. 2020;12(12):e11965. doi: 10.7759/cureus.11965.

----------------------

Witherspoon L, Nham E, Abdi H, Dergham A, Skinner T, Oake JS, et al. [Is it time to rethink how we page physicians? Understanding paging patterns in a tertiary care hospital.](https://pubmed-ncbi-nlm-nih-gov.proxy.kib.ki.se/31870370/) BMC Health Serv Res. 2019;19(1):992. doi: 10.1186/s12913-019-4844-0.

----------------------

Yu EJ, Lee EN. [Development and validation of a Nursing Work Interruption Scale.](https://pubmed-ncbi-nlm-nih-gov.proxy.kib.ki.se/36294067/) Int J Environ Res Public Health. 2022;19(20):13487. doi: 10.3390/ijerph192013487.

----------------------

----------------------

**I. INTERPERSONAL INTERACTIONS & SOCIAL CLIMATE**

**Lacking redress of grievances** (GCNFL5)

Search strategy: (Grievance or complaints) AND (Physician or Health care professional) AND (burnout)

----------------------------

Chesak SS, Salinas M, Abraham H, Harris CE, Carey EC, Khalsa T, et al.  [Experiences of gender inequity among women physicians across career stages: Findings from Participant Focus Groups.](https://pubmed-ncbi-nlm-nih-gov.proxy.kib.ki.se/35415713/) Womens Health Rep (New Rochelle). 2022;3(1):359-68.

----------------------

----------------------

**J. WORKLOAD & ACTIVITIES**

**Handles patients who cannot give a history**

Search strategy: (Patient cannot give history) AND (Physician or Health care professional) AND (burnout)

----------------------------

No results from any of the searches

----------------------------

----------------------------

**No separate time for non-clinical duties**

Search strategy: [(Separate time for other tasks)OR (Separate time for non-clinical tasks)] AND (Physician or Health care professional) AND (burnout)

----------------------------

Aziz F, Talhelm L, Keefer J, Krawiec C.  [Vascular surgery residents spend one fifth of their time on electronic health records after duty hours.](https://pubmed-ncbi-nlm-nih-gov.proxy.kib.ki.se/31010521/)

J Vasc Surg. 2019;69(5):1574-9. doi: 10.1016/j.jvs.2018.08.173.

----------------------------

Banerjee G, Mitchell JD, Brzezinski M, DePorre A, Ballard HA. [Burnout in academic physicians.](https://pubmed-ncbi-nlm-nih-gov.proxy.kib.ki.se/37309180/) Perm J. 2023;27(2):142-9. doi: 10.7812/TPP/23.032.

----------------------------

Egan HM, Swanson MB, Ilko SA, Pomeranz KA, Mohr NM, Ahmed A. [High-efficiency practices of residents in an academic emergency department: A Mixed-methods Study.](https://pubmed-ncbi-nlm-nih-gov.proxy.kib.ki.se/34027282/) AEM Educ Train. 2020;5(3):e10517. doi: 10.1002/aet2.10517.

----------------------------

Jones CE, Fox ED, Holsten SB, White CQ, Sayyid RK, O'Keeffe T, et al. [Burnout reduction in acute care surgeons: Impact of faculty schedule change at a level 1 trauma and tertiary care center.](https://pubmed-ncbi-nlm-nih-gov.proxy.kib.ki.se/35788582/)J Trauma Acute Care Surg. 2022;93(4):439-45. doi: 10.1097/TA.0000000000003736.

----------------------------

Ozkula G, Durukan, E. [Burnout syndrome among physicians: The role of socio-demographic characteristics.](https://web-p-ebscohost-com.proxy.kib.ki.se/ehost/viewarticle/render?data=dGJyMPPp44rp2%2fdV0%2bnjisfk5Ie46bFRr6ixSrOk63nn5Kx95uXxjL6prUmxpbBIsKqeSbCwslC4prc4zsOkjPDX7Ivf2fKB7eTnfLuvs02xprZNs66ki%2bfau3nhq7VQs6quRa%2fc4lGrqrNQ4qO2frGoq33iqrdN4dquTN%2fYs1jw2%2bKB8Zzqeezdu33snOJ6u9nnhrCmpIzf3btZzJzfhrunsEu2p7VIr6%2bkfu3o63nys%2bSN6uLyffbq&vid=11&sid=ac578540-1fd9-458d-8f32-ed495cd04ab5@redis) Dusunen Adam Journal of Psychiatry & Neurological Sciences. 2017; 30(2): 136-44. DOI: 10.5350/DAJPN2017300207

----------------------------

Perumalswami CR, Takenoshita S, Tanabe A, Kanda R, Hiraike H, Okinaga H, et al. Workplace resources, mentorship, and burnout in early career physician-scientists: a cross sectional study in Japan. BMC Med Educ. 2020;20(1):178. doi: 10.1186/s12909-020-02072-x.

----------------------------

Quirk R, Rodin H, Linzer M.  [Targeting causes of burnout in residency: An innovative approach used at Hennepin healthcare.](https://pubmed-ncbi-nlm-nih-gov.proxy.kib.ki.se/33496434/) Acad Med. 2021;96(5):690-4.

----------------------

Toyoshima M, Takenoshita S, Hasegawa H, Kimura T, Nomura K. E[xperiences of negotiations for improving research environment and burnout among young physician researchers in Japan.](https://pubmed-ncbi-nlm-nih-gov.proxy.kib.ki.se/32698340/) Int J Environ Res Public Health. 2020;17(14):5221. doi: 10.3390/ijerph17145221.

----------------------------

----------------------------

**Performs tasks that seem pointless** (GCNFL7)

Search strategy: [(Pointless tasks) OR (Illegitimate tasks) OR (Bureaucratic tasks)] AND AND (Physician or Health care professional) AND (burnout)

----------------------------

Anskär E, Lindberg M, Falk M, Andersson A. [Legitimacy of work tasks, psychosocial work environment, and time utilization among primary care staff in Sweden.](https://pubmed-ncbi-nlm-nih-gov.proxy.kib.ki.se/31682152/) Scand J Prim Health Care. 2019;37(4):476-83. doi: 10.1080/02813432.2019.1684014.

----------------------------

Ash JS, Berg M, Coiera E. Some unintended consequences of information technology in health care: the nature of patient care information system related errors. J Am Med Inform Assoc 2003; 11 (2): 104–12.

-------------------------------

Aziz F, Talhelm L, Keefer J, Krawiec C.  [Vascular surgery residents spend one fifth of their time on electronic health records after duty hours.](https://pubmed-ncbi-nlm-nih-gov.proxy.kib.ki.se/31010521/)

J Vasc Surg. 2019;69(5):1574-9. doi: 10.1016/j.jvs.2018.08.173.

----------------------------

Banerjee G, Mitchell JD, Brzezinski M, DePorre A, Ballard HA. [Burnout in academic physicians.](https://pubmed-ncbi-nlm-nih-gov.proxy.kib.ki.se/37309180/) Perm J. 2023;27(2):142-9. doi: 10.7812/TPP/23.032.

----------------------------

Barak-Corren Y, Wolf R, Rozenblum R, Creedon JK, Lipsett SC, Lyons TW, et al.. [Harnessing the power of generative AI for clinical summaries: Perspectives from emergency physicians.](https://pubmed-ncbi-nlm-nih-gov.proxy.kib.ki.se/38483426/) Ann Emerg Med. 2024:S0196-0644(24)00078-7. doi: 10.1016/j.annemergmed.2024.01.039

----------------------

Belkić K, Rustagi N. Job stressors in relation to burnout and compromised sleep among academic physicians in India. Work. 2024; 78: 505–525.

--------------------------------

Bernat JL. [Challenges to ethics and professionalism facing the contemporary neurologist.](https://pubmed-ncbi-nlm-nih-gov.proxy.kib.ki.se/25171931/) Neurology. 2014;83(14):1285-93. doi: 10.1212/WNL.0000000000000845.

--------------------------------

Borritz M, Rugulies R, Bjorner JB, Villadsen E, Mikkelsen OA, Kristensen TS. [Burnout among employees in human service work: design and baseline findings of the PUMA study.](https://pubmed-ncbi-nlm-nih-gov.proxy.kib.ki.se/16449044/) Scand J Public Health. 2006;34(1):49-58. doi: 10.1080/14034940510032275.

--------------------------------

Brulin E, Ekberg K, Landstad BJ, Lidwall U, Sjöström M, Wilczek A. [Money talks: performance-based reimbursement systems impact on perceived work, health and patient care for physicians in Sweden.](https://pubmed-ncbi-nlm-nih-gov.proxy.kib.ki.se/37484100/) Front Psychol. 2023;14:1216229. doi: 10.3389/fpsyg.2023.1216229.

--------------------------------

Budd J. Burnout related to electronic health record use in Primary Care. J Prim Care Community Health2023;14:21501319231166921.doi 10.1177/21501319231166921

--------------------------------

Coiera E, Ash J, Berg M. The unintended consequences of health information technology revisited. Yearb Med Inform 2016; 25 (01): 163–9.

--------------------------------

Contratto E, Romp K, Estrada C, Agne A, Willett L. Physician order entry clerical support improves physician satisfaction and productivity. South Med J. 2017;110(5):363-8.

--------------------------------

Cullati S, Semmer NK, Tschan F, Choupay G, Chopard P, Courvoisier DS. [When illegitimate tasks threaten patient safety culture: a cross-sectional survey in a tertiary hospital.](https://pubmed-ncbi-nlm-nih-gov.proxy.kib.ki.se/37744414/) Int J Public Health. 2023;68:1606078. doi: 10.3389/ijph.2023.1606078.

--------------------------------

Dale J, Potter R, Owen K, Parsons N, Realpe A, Leach J. R[etaining the general practitioner workforce in England: what matters to GPs? A cross-sectional study.](https://pubmed-ncbi-nlm-nih-gov.proxy.kib.ki.se/26475707/) BMC Fam Pract. 2015;16:140. doi: 10.1186/s12875-015-0363-1.

--------------------------------

DesRoches CM, Campbell EG, Rao SR, et al. Electronic health records in ambulatory care—a national survey of physicians. N Engl J Med 2008; 359 (1): 50–60.

--------------------------------

DiAngi YT, Stevens LA, Halpern-Felsher B, et al. Electronic health record (EHR) training program identifies a new tool to quantify the EHR time burden and improves providers’ perceived control over their workload in the EHR. JAMIA Open 2019; 2 (2): 222–30.

--------------------------------

Eckleberry-Hunt J, Kirkpatrick H, Taku K, Hunt R, Vasappa R. Relation between physicians' work lives and happiness South Med J.2016;109(4):207-12. doi: 10.14423/SMJ.0000000000000437.

-----------------

Holzer E, Tschan F, Kottwitz MU, Beldi G, Businger AP, Semmer NK. [The workday of hospital surgeons: what they do, what makes them satisfied, and the role of core tasks and administrative tasks; a diary study.](https://pubmed-ncbi-nlm-nih-gov.proxy.kib.ki.se/31412843/) BMC Surg. 2019;19(1):112. doi: 10.1186/s12893-019-0570-0.

-----------------

Kilponen K, Huhtala M, Kinnunen U, Mauno S, Feldt T. [Illegitimate tasks in health care: Illegitimate task types and associations with occupational well-being.](https://pubmed-ncbi-nlm-nih-gov.proxy.kib.ki.se/33829574/) J Clin Nurs. 2021;30(13-14):2093-106. doi: 10.1111/jocn.15767.

-----------------

Kruse CS, Mileski M, Dray G, Johnson Z, Shaw C, Shirodkar H. [Physician burnout and the electronic health record leading up to and during the first year of COVID-19: Systematic review.](https://pubmed-ncbi-nlm-nih-gov.proxy.kib.ki.se/35120019/) J Med Internet Res. 2022;24(3):e36200. doi: 10.2196/36200.

----------------------

Marx R, Kahn JG. [A Narrative review of slow medicine outcomes.](https://pubmed-ncbi-nlm-nih-gov.proxy.kib.ki.se/34772782/) J Am Board Fam Med. 2021;34(6):1249-1264. doi: 10.3122/jabfm.2021.06.210137.

----------------------

McGowan Y, Humphries N, Burke H, Conry M, Morgan K. [Through doctors' eyes: A qualitative study of hospital doctor perspectives on their working conditions.](https://pubmed-ncbi-nlm-nih-gov.proxy.kib.ki.se/23480457/) Br J Health Psychol. 2013;18(4):874-91. doi: 10.1111/bjhp.12037.

------------------------

Mishra P, Kiang JC, Grant RW. Association of medical scribes in primary care with physician workflow and patient experience. JAMA Intern Med 2018; 178 (11): 1467–72.

------------------------

Moy AJ, Schwartz JM, Chen R, Sadri S, Lucas E, Cato KD, Rossetti SC. [Measurement of clinical documentation burden among physicians and nurses using electronic health records: a scoping review.](https://pubmed-ncbi-nlm-nih-gov.proxy.kib.ki.se/33434273/) J Am Med Inform Assoc. 2021;28(5):998-1008. doi: 10.1093/jamia/ocaa325.

----------------------

Nedić O, Belkić K. Job stressors and burnout among nurses and primary-care physicians working at a dedicated outpatient respiratory center for patients with suspected or confirmed COVID-19. Am J Ind Med. 2023; 66 (6): 510-28.

--------------------------------

Otokiti AU, Craven CK, Shetreat-Klein A, Cohen S, Darrow B. Beyond getting rid of stupid stuff in the electronic health record (Beyond-GROSS): Protocol for a user-centered, mixed-method intervention to improve the electronic health record system. JMIR Res Protoc. 2021; 10(3): e25148 doi: 10.2196/25148

-------------------------------

Ozkula G, Durukan, E. [Burnout syndrome among physicians: The role of socio-demographic characteristics.](https://web-p-ebscohost-com.proxy.kib.ki.se/ehost/viewarticle/render?data=dGJyMPPp44rp2%2fdV0%2bnjisfk5Ie46bFRr6ixSrOk63nn5Kx95uXxjL6prUmxpbBIsKqeSbCwslC4prc4zsOkjPDX7Ivf2fKB7eTnfLuvs02xprZNs66ki%2bfau3nhq7VQs6quRa%2fc4lGrqrNQ4qO2frGoq33iqrdN4dquTN%2fYs1jw2%2bKB8Zzqeezdu33snOJ6u9nnhrCmpIzf3btZzJzfhrunsEu2p7VIr6%2bkfu3o63nys%2bSN6uLyffbq&vid=11&sid=ac578540-1fd9-458d-8f32-ed495cd04ab5@redis) Journal of Psychiatry & Neurological Sciences. 2017; 30(2): 136-44. DOI: 10.5350/DAJPN2017300207

----------------------

Portier G, Mathonnet M. [The workplace quality of life of university hospital digestive surgeons: Results of a 2019 nationwide survey.](https://pubmed-ncbi-nlm-nih-gov.proxy.kib.ki.se/34016570/) J Visc Surg. 2022;159(3):201-5. doi: 10.1016/j.jviscsurg.2021.03.010.

------------------------------

Scheepers R, Silkens M, van den Berg J, Lombarts K. [Associations between job demands, job resources and patient-related burnout among physicians: results from a multicentre observational study.](https://pubmed-ncbi-nlm-nih-gov.proxy.kib.ki.se/32973063/) BMJ Open. 2020;10(9):e038466. doi: 10.1136/bmjopen-2020-038466.

------------------------------

Seelandt JC, Tschan F, Keller S, Beldi G, Jenni N, Kurmann A, Candinas D, Semmer NK. [Assessing distractors and teamwork during surgery: developing an event-based method for direct observation.](https://pubmed-ncbi-nlm-nih-gov.proxy.kib.ki.se/25013008/) BMJ Qual Saf. 2014;23(11):918-29. doi: 10.1136/bmjqs-2014-002860.

------------------------------

Shanafelt TD, Dyrbye LN, Sinsky C, Hasan O, Satele D, Sloan J, et al. Relationship between clerical burden and characteristics of the electronic environment with physician burnout and professional satisfaction. Mayo Clin Proc 2016; 91(7): 836-48. doi.org/10.1016/j.mayocp.2016.05.007

------------------------------

Shoureshi P, Guerre M, Seideman CA, Callejas DG, Amling CL, Bassale S, et al.  [Addressing Burnout in urology: A qualitative assessment of interventions.](https://pubmed-ncbi-nlm-nih-gov.proxy.kib.ki.se/37145567/)

Urol Pract. 2022;9(1):101-7. doi: 10.1097/UPJ.0000000000000282.

------------------------------

Thun S, Halsteinli V, Løvseth L. [A study of unreasonable illegitimate tasks, administrative tasks, and sickness presenteeism amongst Norwegian physicians: an everyday struggle?](https://pubmed-ncbi-nlm-nih-gov.proxy.kib.ki.se/29871623/) BMC Health Serv Res. 2018;18(1):407. doi: 10.1186/s12913-018-3229-0.

------------------------------

Verret CI, Nguyen J, Verret C, Albert TJ, Fufa DT. [How do areas of work life drive burnout in orthopaedic attending surgeons, fellows, and residents?](https://pubmed-ncbi-nlm-nih-gov.proxy.kib.ki.se/32858718/) Clin Orthop Relat Res. 2021;479(2):251-62. doi: 10.1097/CORR.0000000000001457.

------------------------------

Werdecker L, Esch T. [Burnout, satisfaction and happiness among German general practitioners (GPs): A cross-sectional survey on health resources and stressors.](https://pubmed-ncbi-nlm-nih-gov.proxy.kib.ki.se/34143849/) PLoS One. 2021;18;16(6):e0253447.
