# Supplementary material for: Toward better prevention of physician burnout: insights from individual participant data using the MD-specific Occupational Stressor Index and organizational interventions
Source: Front Public Health. 2025 Mar 19;13:1514706. doi: 10.3389/fpubh.2025.1514706 (PMC11961930; doi:10.3389/fpubh.2025.1514706)
Supplement: Supplementary file 3 [file Data_Sheet_3.docx]

**SUPPLEMENT 3**

**Univariate Data from the Physician-specific Occupational Stressor Index (OSI) Questionnaire**

**among the Physicians with Individual Participant Data**

More complete data are presented herein concerning the work environment of the physicians including in the Individual Participant Data analysis. The order of presentation usually follows that of the Physician-Specific OSI Questionnaire.

Both the Physician-Specific OSI Questionnaire and the Physician-Specific OSI score sheet can be accessed at: Supplemental Digital Content, <http://links.lww.com/SMJ/A230> and <http://links.lww.com/SMJ/A231>, respectively.

Please note, the main headings of the OSI questionnaire are given to helping orient the respondent physician. These main headings are labeled with a capital letter, highlighted and bolded. Within a given main heading, all the queries are identified by that capital letter, followed by the number indicating the order of appearance in the questionnaire.

An example: for the main heading **C**. **WORK HOURS & SCHEDULING**, question C2: How many days do you usually work per week?

A single or several queries from the OSI questionnaire may be used to produce an OSI element, from which the scoring of the OSI is actually done. The OSI element is denoted by its acronym and number followed by the descriptive name in bolded capital letters.

An example: There are 5 quantitative questions concerning rest breaks from the physician-specific OSI questionnaire. These are: C7 Rest break frequency, C8 Rest break length, C9 True rest breaks free from work obligations and C10 Length of time without even a short break. The responses to these 5 questions are taken together to the score the OSI element: GH4 **LACK OF REST BREAKS**. GH4 indicates the general level (G), high demand aspect (H) and 4 as the 4^th^ OSI element within GH. There is also an open-ended item within C9: If not true rest breaks, why not?

The OSI model is formed from a two-dimensional matrix. The upper three rows indicate the level of information-transmission: input, decision making, and task performance. These levels are basic to cognitive ergonomics, and enable objective assessment of stressors and how they impact on the central nervous system. The fourth level is general. There are seven columns corresponding to the OSI aspects: underload, high demand, strictness, extrinsic time pressure, noxious exposures, threat avoidant vigilance, and conflict/uncertainty. Every element of the OSI is scored from 0 (absent) to 2 (strongly present). The sum of all the scores of all the OSI elements generates the total OSI. For more information about the theoretical basis and computation of the OSI, see [e.g. (1)]^[[1]](#endnote-1)^. See p. 9 of this Supplement for a list of acronyms.

To preserve confidentiality, insofar as any result pertained to five or fewer of the physicians included in this analysis of the individual participant data, we have avoided stating the exact number. Otherwise, all available data are presented. Insofar as the sum is less expected (usually 97), the remaining data are missing.

=========================================================================

**A. DURATION OF WORK AS PHYSICIANS & TYPE OF EMPLOYMENT**

=========================================================================

A3: **All 97 participants were employed full time as physicians**

A4: **Working years as a physician**

|  | Count | % Valid |
| --- | --- | --- |
| < 1 year | 11 | 11.3 |
| 1 to 5 years | 48 | 49.5 |
| 6 to 10 years | 19 | 19.6 |
| > 10 years | 19 | 19.6 |

======================================

**B. TYPE OF PRACTICE/SETTING**

=======================================

B1: **Level of training**

|  | Count | % Valid |
| --- | --- | --- |
| General practice | 21 | 21.6 |
| Resident | 64 | 66.0 |
| Attending physician specialist | 12 | 12.4 |

**Area of Practice**

| General practice/community medicine | 34 | 35.0 |
| --- | --- | --- |
| Surgical/anesthesia/emergency medicine | 32 | 33.0 |
| Internal medicine/pediatrics/psychiatry/dermatology/combined specialties | 31 | 32.0 |

B2: **All 97 participants were employed in the public sector**

B3. **Percentage of inpatients under one’s care**

|  | Count | % Valid |
| --- | --- | --- |
| < 10% | 46 | 47.4 |
| 10% ≤ x ≤ 50 | 28 | 28.9 |
| > 50% | 23 | 23.7 |

B4. **Percentage of patients with end stage/incurable disease under one’s care**

|  | Count | % Valid |
| --- | --- | --- |
| < 10% | 62 | 63.9 |
| 10% ≤ x ≤ 20 | 23 | 23.7 |
| 20% < x < 50 | 6 | 6.2 |
| ≥ 50% | 6 | 6.2 |

B5. **Percentage of patients in emergency status under one’s care**

|  | Count | % Valid |
| --- | --- | --- |
| < 10% | 59 | 60.8 |
| 10% ≤ x < 50 | 32 | 33.0 |
| ≥ 50% | 6 | 6.2 |

B6. **Nearly all 97 physicians worked only at the one institution which was their sole employment.**

=====================================

**C. WORK HOURS & SCHEDULING**

=====================================

C1: **Length of usual work hours/day**

| N | Mean | Standard deviation | Median | Interquartile range |
| --- | --- | --- | --- | --- |
| 97 | 13.3 | 2.5 | 12.0 | 4.0 |

C2: **Usual number of workdays/week**

| N | Mean | Standard deviation | Median | Interquartile range |
| --- | --- | --- | --- | --- |
| 97 | 5.88 | 1.1 | 6.0 | 1.0 |

**Usual total weekly hours (C1 x C2) :**

| N | Mean | Standard deviation | Median | Interquartile range |
| --- | --- | --- | --- | --- |
| 97 | 79.5 | 26.2 | 72 | 36 |

C4: **Calls/email outside work hours**: (0=never, 1=rarely, 2=occasionally, 3=frequently)

| N | Mean | Standard deviation | Median | Interquartile range |
| --- | --- | --- | --- | --- |
| 97 | 1.68 | 1.0 | 2.0 | 2.0 |

C18 : **Works at home for job:** 0=never, 0.5=rarely, 1.5=sometimes, 2=frequently

| N | Mean | Standard deviation | Median | Interquartile range |
| --- | --- | --- | --- | --- |
| 97 | 0.92 | 0.69 | 0.5 | 1.0 |

GH2: **LONG WORK HOURS**

0 Not more than 40h/week

0.5 40 to 42h/week

1 Frequently > 42h/week

2 Frequently > 48h/week

Calculate from C1-C2, add 0.5 points if C4=c or d (called or emailed at home)

or C18=c or d (works at home) and hours not included, to maximum 2 points,

| N | Mean | Standard deviation | Median | Interquartile range |
| --- | --- | --- | --- | --- |
| 97 | 1.93 | 0.22 | 2 | 0 |

---------------------------------

C5:GH6 **INSUFFICIENT PAID VACATION**

0 Over four weeks per year

1 Three to four weeks per year

1.5 Two weeks per year

2 Less than two weeks per year

Add 0.5 to 1 point if vacation is actually formal; work is performed during vacation to maximum 2 points.

| N | Mean | Standard deviation | Median | Interquartile range |
| --- | --- | --- | --- | --- |
| 97 | 1.09 | 0.81 | 1.0 | 2.0 |

C7: **Rest break frequency**

2=Never 1.5=Rarely 1=Occasionally 0=Frequently

| N | Mean | Standard Deviation | Median | Interquartile range |
| --- | --- | --- | --- | --- |
| 97 | 1.19 | 0.43 | 1.0 | 0.5 |

-

| C8: **Rest break length** | N | % |
| --- | --- | --- |
| Only short ( < 15 minutes) | 71 | 73.2 |
| At least some breaks > 30 minutes | 26 | 26.8 |

| C9: **True rest breaks free from work obligations** | N | % |
| --- | --- | --- |
| No | 65 | 67.0 |
| Yes | 32 | 33.0 |

C10: **Usual number of hours without even a short rest break**

| N | Mean | Standard Deviation | Median | Interquartile range |
| --- | --- | --- | --- | --- |
| 97 | 2.67 | 1.3 | 2.1 | 0 |

GH4: **LACK OF REST BREAKS**

0 C7 = frequently has rest breaks

1 C7 = occasionally has rest breaks

1.5 C7 = rarely has rest breaks

2 C7 =never has rest breaks

Add 0.5 points if C8=only short breaks, C9=not true rest breaks, if C10 > 2 hours, to a maximum of 2 points.

| N | Mean | Standard Deviation | Median | Interquartile range |
| --- | --- | --- | --- | --- |
| 97 | 1.78 | 0.42 | 2.0 | 0.38 |

---------------------------------

GH5: **NIGHT SHIFT WORK**

0 C3=regular work schedule AND C11=no night shift work

0.5 C3=irregular work schedule AND C11=no night shift work

1 C11=yes, night shift only

1.5 C11=yes, b (rotating night shift, up to four night shifts/month)

1.75 C11=yes, b (rotating night shift, at least once per week but not more than every fourth night)

2 C11=yes, b (rotating night shift, at least every third night)

Add 0.5 points if < 24 free time after working a rotating night shift, no guaranteed relief, up to maximum 2 points.

| N | Mean | Standard Deviation | Median | Interquartile range |
| --- | --- | --- | --- | --- |
| 97 | 1.53 | 0.80 | 2.0 | 0.5 |

---------------------------------

C16=GS4 **DIFFICULT TO TAKE TIME OFF**

0) Not at all 1.5) Somewhat

1) A little 2) Very much

| N | Mean | Standard Deviation | Median | Interquartile range |
| --- | --- | --- | --- | --- |
| 97 | 1.32 | 0.48 | 1.5 | 0.5 |

---------------------------------

C17= GS5 **WORK SCHEDULE INFLUENCE**

0) Complete, it is entirely up to me.

0.5) To a large extent, I mainly decide on my work schedule.

1.5) A little, but mainly my schedule depends on others, or is decided by others.

2) None at all, my schedule depends on others or is decided by others, and I have no say about it.

| N | Mean | Standard Deviation | Median | Interquartile range |
| --- | --- | --- | --- | --- |
| 97 | 1.49 | 0.45 | 1.5 | 0 |

==============================================================

**D. SALARY, POSSIBILITIES FOR ADVANCEMENT & RECOGNITION**

==============================================================

D1 = GU1 **FIXED PAY**

0) Salary is based on one’s own work: number of patients, interventions, hours worked etc.

1) Salary is based on group work: number of patients, interventions, hours worked etc.

2) Fixed pay, irrespective of the amount of work.

| N | Mean | Standard deviation | Median | Interquartile range |
| --- | --- | --- | --- | --- |
| 97 | 1.65 | 0.76 | 2.0 | 0 |

--------------------------------

D2 = GU2 **INADEQUATE PAY**

0) My pay covers substantially more than my basic needs and those of my family.

0.5) Covers a bit more than my basic needs and those of my family.

1.5) Just barely covers my basic needs and those of my family.

2) Totally inadequate to meet my basic needs and those of my family.

| N | Mean | Standard Deviation | Median | Interquartile range |
| --- | --- | --- | --- | --- |
| 97 | 0.67 | 0.57 | 0.5 | 1.0 |

--------------------------------

D3: **Possibilities for upgrade**

|  | Count | % Valid |
| --- | --- | --- |
| No | 18 | 18.6 |
| Yes | 79 | 81.4 |

D3: *If yes,* **is there support and encouragement to do so**?

0) Definitely yes.

0.5) Yes, to some extent.

1) Not really, but there is no active opposition to such efforts.

1.5) No, there is active opposition to such efforts.

2) No, this is viewed very unfavorably and inevitably arouses negative comments or jealousy

| N | Mean | Standard Deviation | Median | Interquartile range |
| --- | --- | --- | --- | --- |
| 78 | 0.42 | 0.38 | 0.5 | 0.5 |

---------------------------

D4 = GU4 **LACK OF RECOGNITION OF GOOD WORK**

Is good work recognized at your workplace?

0) Definitely yes 1.5) Not very much.

0.5) Yes, to some extent. 2) Not at all.

| N | Mean | Standard Deviation | Median | Interquartile range |
| --- | --- | --- | --- | --- |
| 97 | 0.42 | 0.49 | 0.5 | 0.5 |

===============================

**E. WORKING CONDITIONS**

===============================

E1= INOX1 **GLARE EXPOSURE**

0) Never 1) Occasionally (in the Operating Room or elsewhere)

0.5) Rarely 2) Frequently (in the Operating Room or elsewhere)

| N | Mean | Standard Deviation | Median | Interquartile range |
| --- | --- | --- | --- | --- |
| 97 | 0.52 | 0.56 | 0.5 | 1.0 |

---------------------------

E2= IAVOI2 **ENCOUNTERS VISUALLY DISTURBING SCENES**

0) Never 1) Occasionally

0.5) Rarely 2) Frequently

| N | Mean | Standard Deviation | Median | Interquartile range |
| --- | --- | --- | --- | --- |
| 97 | 0.84 | 0.60 | 0.5 | 0.5 |

E3: **Listens to emotionally disturbing accounts**

0) Never 1) Occasionally

0.5) Rarely 2) Frequently

| N | Mean | Standard Deviation | Median | Interquartile range |
| --- | --- | --- | --- | --- |
| 97 | 1.05 | 0.58 | 1.0 | 0.5 |

IAVOI3 **LISTENS TO EMOTIONALLY DISTURBING ACCOUNTS**

0) Never 1) Occasionally

0.5) Rarely 2) Frequently

Add 1 point if B4 = 1.5 or 2) (large percentage of patients with end-stage or incurable disease, 0.5 points if B4=1,

add 1 point if F3=1.5 or 2 (patient suicide, and 0.5 points if F3=0.5 (heard about patient suicide), to maximum of 2 points

| N | Mean | Standard Deviation | Median | Interquartile range |
| --- | --- | --- | --- | --- |
| 97 | 1.40 | 0.66 | 2.0 | 1.0 |

--

-----------------

E4 =ONOX1 **HEAVY LIFTING**

2) Regularly must lift patients or other heavy lifting ≥50 kg

1) Yes, but < 50kg

0) No, rarely does any heavy lifting during the workday.

| N | Mean | Standard Deviation | Median | Interquartile range |
| --- | --- | --- | --- | --- |
| 97 | 0.11 | 0.45 | 0.0 | 0 |

-----------------

E5 = ONOX2 **VIBRATION EXPOSURE**

0) Only very rarely or no vibration exposure

0.5) Uses vibrating hand-tools, up to 5 hours/week

1)Uses vibrating hand-tools, > 5 hours/week

| N | Mean | Standard Deviation | Median | Interquartile range |
| --- | --- | --- | --- | --- |
| 97 | 0.06 | 0.21 | 0.0 | 0 |

--------------------------

GNOX1 **HEAT EXPOSURE**

E6

0) It rarely or never gets hotter than 25° C (77° F) at work.

0.5) It rarely or never gets hotter than 30° C (86° F) at work.

1) It occasionally or often gets hotter than 30° C (86° F).

Add 0.5 points if inadequate ventilation (E13)

| N | Mean | Standard Deviation | Median | Interquartile range |
| --- | --- | --- | --- | --- |
| 97 | 0.61 | 0.60 | 0.5 | 1.0 |

--------------------------------------

GNOX2 **COLD EXPOSURE**

E7

0) It rarely or never gets colder than 20° C (68° F) at work.

0.5) It rarely or never gets colder than 18° C (64° F) at work.

1) The heating system is poor, with temperatures <18° C (64° F).

Add 0.5 points if drafty (E14)

| N | Mean | Standard Deviation | Median | Interquartile range |
| --- | --- | --- | --- | --- |
| 97 | 0.27 | 0.35 | 0.0 | 0.5 |

--------------------------------------

E8 = GNOX3 **EXPOSURE TO FUMES OR DUST**

0=rarely or never

1=at least occasionally

| N | Mean | Standard Deviation | Median | Interquartile range |
| --- | --- | --- | --- | --- |
| 97 | 0.29 | 0.46 | 0.0 | 1.0 |

--------------------------------------

E9: **Radiation exposure**

|  | Count | % Valid |
| --- | --- | --- |
| Yes | 29 | 29.9 |
| No | 68 | 70.1 |

E9: **Radiation badge for those with exposure?**

|  | Count | % Valid |
| --- | --- | --- |
| No | 19 | 65.5 |
| Yes | 10 | 34.5 |

---------------------------------------

E10: **Acute hazards**

E10a: **Threat of violence**

|  | Count | % Valid |
| --- | --- | --- |
| Yes | 37 | 38.1 |
| No | 60 | 61.9 |

E10b: **Infection risk**

|  | Count | % Valid |
| --- | --- | --- |
| Yes | 84 | 86.6 |
| No | 13 | 13.4 |

-

OVAOIT: **HAZARDOUS TASK PERFORMANCE**

1 E10= No acute hazards reported

2 E10 = a-d Acute hazards present

##### Add 1 for Radiation exposure, E9 to a maximum of 2 points

| N | Mean | Standard Deviation | Median | Interquartile range |
| --- | --- | --- | --- | --- |
| 97 | 1.94 | 0.24 | 2.0 | 0 |

v--------------------------------------

E11: **Shares an office**

|  | Count | % Valid |
| --- | --- | --- |
| Yes | 68 | 70.1 |
| No | 29 | 29.9 |

E11: **Number of persons sharing an office** (among those who share an office)

| N | Mean | Standard Deviation | Median | Interquartile range |
| --- | --- | --- | --- | --- |
| 67 | 4.94 | 3.0 | 4 | 3 |

E11: **Cramped office**

|  | Count | % Valid |
| --- | --- | --- |
| Yes | 40 | 41.2 |
| No | 57 | 58.8 |

E11: **Own desk for those who share an office**

|  | Count | % Valid |
| --- | --- | --- |
| No | 51 | 79.7 |
| Yes | 13 | 20.3 |

E11: **Has to look for an empty office to interview or examine patients**

|  | Count | % Valid |
| --- | --- | --- |
| Yes | 56 | 58.9 |
| No | 39 | 41.1 |

E11: **Percent time in the office**

| N | Mean | Standard Deviation | Median | Interquartile range |
| --- | --- | --- | --- | --- |
| 92 | 34.5 | 33.0 | 25 | 55.5 |

E12: **Window in office**

|  | Count | % Valid |
| --- | --- | --- |
| Windowless office | 10 | 10.3 |
| Yes, but no direct view outside | 26 | 26.8 |
| Yes, direct view outside | 61 | 62.9 |

GS2: **CONFINED, WINDOWLESS &/OR POORLY VENTILATED WORK AREA**

Works in >1 location (E11part IV < 50% in office (v81<50)) subtract 1 point to minimum 0

0.5 Spends over 50% of time in a non-confined work area, with a direct window (E12=a) AND (E11 indicates non-confined work area)

1 Spends over 50% of time in a non-confined work area, with an indirect window E12=b) AND (E11 indicates non-confined work area)

1.5 Non-confined work area without a window E12=c) AND (E11 indicates non-confined work area-shares with at most 1 other colleagues)

1.5 Confined or crowded space with an indirect window (E12=b) AND (E11 indicates very crowded work area or 2+ colleagues in an office)

2 Windowless and confined/crowded space (E12=c) AND (E11 indicates very crowded work area or 2+ colleagues in an office)

Add 0.5 points if E13=no, inadequate ventilation, to a maximum of 2 points.

| N | Mean | Standard Deviation | Median | Interquartile range |
| --- | --- | --- | --- | --- |
| 97 | 1.48 | 0.77 | 2.0 | 1 |

GS3: **LACKS AUTONOMOUS WORKSPACE**

0 E11 (Has own desk/workspace and has own office)

1 E11 (Has own desk or workspace but shares an office)

2 E11 (No autonomous workspace)

##### Add 1 point to maximum of 2 if (E11) (V80=1) seeks free office space to talk with or examine patients

| N | Mean | Standard Deviation | Median | Interquartile range |
| --- | --- | --- | --- | --- |
| 96 | 1.40 | 0.91 | 2.0 | 2 |

================================

**F. MISHAPS AT WORK**

===============================

F1=GAVOI1: **EXPERIENCED ACCIDENT OR INJURY AT WORK**

0 F1 = No 1 F1 = Yes, if not serious 2 F1 = Yes, if serious

| N | Mean | Standard Deviation | Median | Interquartile range |
| --- | --- | --- | --- | --- |
| 97 | 0.14 | 0.35 | 0.0 | 0 |

----------------------

F2=GAVOI2 **WITNESSED ACCIDENT OR INJURY AT WORK**

0 F2 = d (never heard about or witnessed a serious accident at work)

0.5 F2 = c (heard about but never witnessed a serious accident at work)

1 F2 = b (witnessed serious accident at work)

2 F2 = a (witnessed fatal accident at work)

| N | Mean | Standard Deviation | Median | Interquartile range |
| --- | --- | --- | --- | --- |
| 97 | 0.13 | 0.33 | 0.0 | 0 |

----------------------

F3: **Patient suicide**

2 F3 = a Yes, this has happened on several occasions.

1.5 F3 = b Yes, I have had one or two such patients.

0.5 F3 = c No, but it has happened to colleague(s) with whom I work.

0 F3 = d No, it has never happened to me or to colleague(s) with whom I work

| N | Mean | Standard Deviation | Median | Interquartile range |
| --- | --- | --- | --- | --- |
| 97 | 0.21 | 0.53 | 0.0 | 0 |

------------------------------------------

F4: **Suicide among colleagues or staff at work**

**Any suicides (attempts or completed) of colleagues or staff at work**

|  | Count | % Valid |
| --- | --- | --- |
| Yes | 24 | 24.7 |
| No | 73 | 75.3 |

If yes, **how many times has a suicide attempt or completed suicide occurred?**

| N | Mean | Standard Deviation | Median | Interquartile range |
| --- | --- | --- | --- | --- |
| 23 | 1.74 | 0.75 | 2 | 1.0 |

If yes, **was the person known to you?**

|  | Count | % Valid |
| --- | --- | --- |
| Yes | 17 | 70.8 |
| No | 7 | 29.2 |

If yes, **did you work directly with the person or persons?**

|  | Count | % Valid |
| --- | --- | --- |
| Yes | 9 | 39.1 |
| No | 14 | 60.9 |

If yes, **did any of these result in an actual suicide?**

|  | Count | % Valid |
| --- | --- | --- |
| Yes | 16 | 69.6 |
| No | 7 | 30.4 |

GAVOI4: **ATTEMPTED/COMPLETED SUICIDE OF PATIENT**(S) **AND/OR PERSON**(S) **AT WORK**

0 F3 = d AND F4 = no: No patient suicide attempt for respondent nor to colleagues. No suicide attempt among colleagues or staff.

0.5 F3 = c Patient suicide attempt to colleagues, but not known to respondent. No suicide attempt among colleagues or staff.

1 F3 = c Patient completed suicide to colleagues, but not known to respondent OR F4 = One suicide attempt at work (V93=1), person not known to respondent.

1.5 F3= b One or two of respondent’s patients have attempted suicide OR F4 >1 suicide attempt at work, persons not known to respondent

2 F3 = a Several of respondent’s patients have attempted suicide (V90=2) OR F4 Suicide attempt at work with person known to respondent.

Add 1 point to 2 maximum if ANY completed suicide.

| N | Mean | Standard Deviation | Median | Interquartile range |
| --- | --- | --- | --- | --- |
| 97 | 0.63 | 0.91 | 0.0 | 2.0 |

-----------------

F5: **Has testified in court as a physician**

|  | Count | % Valid |
| --- | --- | --- |
| Yes | 16 | 16.5 |
| No | 81 | 83.5 |

F7: **Official complaint against the physician**

|  | Count | % Valid |
| --- | --- | --- |
| Yes | 7 | 7.2 |
| No | 90 | 92.8 |

GAVOI3: **TESTIFYING/LITIGATION/COMPLAINTS**

0 F5 = No

1 F5 = Yes, As expert witness,

1.5 F5 = Yes, Testified about a colleague or staff member or official complaint to workplace

2 F5 = Yes, Defendant in a malpractice case

##### Add 0.5 points if publicized on mass media or if a complaint brought to higher body to maximum 2 points.

| N | Mean | Standard Deviation | Median | Interquartile range |
| --- | --- | --- | --- | --- |
| 97 | 0.28 | 0.55 | 0 | 0 |

-----------------

F10 = GAVOI5: **SYSTEM IN PLACE AT WORK IN CASE OF NON-MEDICAL EMERGENCIES**

0) Yes, and I know that it functions properly.

1) Yes, but I do not know how well it actually functions.

2) No, there is not a functioning system in place in case of non-medical emergencies.

| Code | Count | % Valid |
| --- | --- | --- |
| 2 | 21 | 21.65 |
| 1 | 55 | 56.7 |
| 0 | 21 | 21.65 |

========================================

**G. TIME PRESSURE AT WORK**

========================================

G1 = GEP1 **DEADLINE PRESSURE**

**Do you have a deadline by which a given job or task must be completed?**

0) Never 1) Occasionally

0.5) Rarely 2) Frequently

| N | Mean | Standard Deviation | Median | Interquartile range |
| --- | --- | --- | --- | --- |
| 97 | 1.53 | 0.61 | 2.0 | 1.0 |

-----------------

GEP2: **SPEED-UP WITH EMERGENCY WORK TAKEN INTO ACCOUNT**

0 G2=a (rarely or never) 1 G2=b (certain periods of the month or year)

1.5 G2=c (at least weekly but not daily) 2 G2=d (daily)

##### Add 0.5 points if B5=c, add 0.25 points if B5=b to a maximum of 2, to account for emergency work.

| N | Mean | Standard Deviation | Median | Interquartile range |
| --- | --- | --- | --- | --- |
| 97 | 1.45 | 0.56 | 1.5 | 1.0 |

-----------------

G3: **With regard to your workload and time constraints**:

0) It is always possible to complete everything.

0.5) It is usually possible to complete everything.

1) It is sometimes impossible to complete everything, even with maximal effort.

2) It is often objectively impossible to complete everything, even with maximal effort.

| N | Mean | Standard Deviation | Median | Interquartile range |
| --- | --- | --- | --- | --- |
| 97 | 0.81 | 0.42 | 0.5 | 0.5 |

-----------------

OCNFL1**: CONFLICTING TASKS IN TIME AND SPACE**

0 G3 It is always possible to complete everything.

0.5 G3 It is usually possible to complete everything.

1 G3 It is sometimes impossible to complete everything, even with maximal effort.

2 G3 It is often objectively impossible to complete everything, even with maximal effort.

Add 0.5 points for J14 =no special time set aside for non-clinical tasks, J7= cares for newly admitted patients during shift, to max. 2 points

| N | Mean | Standard Deviation | Median | Interquartile range |
| --- | --- | --- | --- | --- |
| 97 | 1.56 | 0.43 | 1.5 | 0.75 |

=======================================================================================================================

**H. PROBLEMS/RESTRICTIONS/CONSTAINTS & INFLUENCE AT WORK**

=======================================================================================================================

| The degree of influence over: | Major (0) | Some (0.5) | Little (1.5) | None (2) |
| --- | --- | --- | --- | --- |
| H1. Number of patients under your care/ outpatient scheduling |  |  |  |  |
| H2. Which clinical tasks or procedures you perform |  |  |  |  |
| H3. Whether and how much you will take on other, non-clinical duties |  |  |  |  |
| H4. Which colleagues and staff you work with |  |  |  |  |
| H5. Planning and policy of your institution (including those regarding indications for medical procedures and for hospital admissions) |  |  |  |  |

H1: **Influence over number of patients under your care/outpatient scheduling**

| N | Mean | Standard Deviation | Median | Interquartile range |
| --- | --- | --- | --- | --- |
| 97 | 0.92 | 0.73 | 0.5 | 1.0 |

**___________________________**

H2 : **Clinical task choice influence**

| N | Mean | Standard Deviation | Median | Interquartile range |
| --- | --- | --- | --- | --- |
| 97 | 0.76 | 0.71 | 0.5 | 1.5 |

**___________________________**

H3 **Non-clinical task choice influence**

| N | Mean | Standard Deviation | Median | Interquartile range |
| --- | --- | --- | --- | --- |
| 97 | 1.06 | 0.66 | 1.5 | 1.0 |

**___________________________**

H4 **Colleagues & staff choice influence**

| N | Mean | Standard Deviation | Median | Interquartile range |
| --- | --- | --- | --- | --- |
| 97 | 1.02 | 0.67 | 0.5 | 1.0 |

**___________________________**

H5 = GS8: **PLANNING/POLICY INFLUENCE**

| N | Mean | Standard Deviation | Median | Interquartile range |
| --- | --- | --- | --- | --- |
| 97 | 1.43 | 0.67 | 1.5 | 1.5 |

**___________________________**

H7*.* **Is your work/clinical judgment is criticized by those who oversee your work??**

2) Often 1) Sometimes 0.5) Rarely 0) Never

| N | Mean | Standard Deviation | Median | Interquartile range |
| --- | --- | --- | --- | --- |
| 97 | 0.84 | 0.51 | 1.0 | 0.5 |

-------------------

H10 = OCNFL2: **PROBLEMS/DEFICIENCIES HINDER PATIENT CARE**

0) a) Never 1) c) Occasionally

0.5) b) Rarely 2) d) Frequently

| N | Mean | Standard Deviation | Median | Interquartile range |
| --- | --- | --- | --- | --- |
| 97 | 0.76 | 0.36 | 1.0 | 0.5 |

***Specified problems that hinder patient care*:**

H10a: **Lack of supplies**

|  | Count | % Valid |
| --- | --- | --- |
| Yes | 41 | 42.3 |

-------------------------

H10b: **Lack of hospital beds**

|  | Count | % Valid |
| --- | --- | --- |
| Yes | 24 | 24.7 |

-------------------------

H10c: **Understaffing**

|  | Count | % Valid |
| --- | --- | --- |
| Yes | 44 | 45.4 |

-------------------------

H10d: **Administrative constraints in ordering supplies**

|  | Count | % Valid |
| --- | --- | --- |
| Yes | 27 | 27.8 |

-------------------------

H103e: **Language barriers**

|  | Count | % Valid |
| --- | --- | --- |
| Yes | 31 | 32.0 |

-------------------------

H103f: **Infrastructural problems**

|  | Count | % Valid |
| --- | --- | --- |
| Yes | 11 | 11.3 |

-------------------------

H103g: **Tenuous patient transport**

|  | Count | % Valid |
| --- | --- | --- |
| Yes | 6 | 6.2 |

-------------------------

H103h: **Delay or inability to obtain medical records**

|  | Count | % Valid |
| --- | --- | --- |
| Yes | 12 | 12.4 |

-------------------------

H103i: **Difficulty in obtaining laboratory results**

|  | Count | % Valid |
| --- | --- | --- |
| Yes | 23 | 23.7 |

-------------------------

H11 = OCNFL3 **INTERRUPTIONS FROM PEOPLE HAMPER TASK PERFORMANCE**

0) Never 1) Occasionally

0.5) Rarely 2) Frequently

| N | Mean | Standard Deviation | Median | Interquartile range |
| --- | --- | --- | --- | --- |
| 97 | 0.87 | 0.60 | 0.5 | 0.5 |

===================================================================================================

**I. INTERPERSONAL INTERACTIONS & SOCIAL CLIMATE**

=====================================================================================================

I1: GCNFL2 **LACKS HELP WITH CLINICAL DIFFICULTIES**

Can you get help for difficult cases and/or clinical dilemmas?

0) Yes, I can almost always count on such help. 1.5) I can’t really count on getting such help.

0.5) Yes, more often than not 2) Rarely or never do I get the help, which I need.

| N | Mean | Standard Deviation | Median | Interquartile range |
| --- | --- | --- | --- | --- |
| 97 | 0.14 | 0.26 | 0.0 | 0.5 |

----------------------------------

I2**: Social climate: In general, how is the social climate at your workplace**?

0) Excellent, we all get along very well together and misunderstanding are very rare.

0.5) Good, most the time we get along well, with few misunderstandings and tensions.

1) Fair, we have our ups-and-downs, and sometimes there are misunderstandings and tensions.

2) Poor, there is a great deal of tension and conflict.

| N | Mean | Standard Deviation | Median | Interquartile range |
| --- | --- | --- | --- | --- |
| 97 | 0.41 | 0.40 | 0.5 | 0.5 |

----------------------------------

I3: **Knowledge display atmosphere: When obliged to display knowledge and/or skills in front of colleagues and/or supervisors**

0) The atmosphere is constructive and conducive to growth and learning

0.5) There is some tension. Oversights and/or lack of knowledge will be noticed and commented upon. If these are of major importance, there may be adverse consequences for me.

1) These occasions are highly unpleasant. Even the slightest oversight or lack of knowledge inevitably becomes a point of ridicule and/or chastisement.

| N | Mean | Standard Deviation | Median | Interquartile range |
| --- | --- | --- | --- | --- |
| 97 | 0.11 | 0.21 | 0.0 | 0 |

----------------------------------

GCNFL1: **EMOTIONALLY CHARGED WORK ATMOSPHERE**

0 I2 = (no) AND I3=0 (Constructive learning atmosphere)

0.5 I2 =0.5

1 I2 =1 (occasionally)

2 I2 =2 (great deal of tension)

Add 0.5 points if I3 = 0.5) (some tension with display of knowledge) and 1 point if I3=1 (high tension with display of knowledge) to a maximum of 2 points.

| N | Mean | Standard Deviation | Median | Interquartile range |
| --- | --- | --- | --- | --- |
| 97 | 0.52 | 0.49 | 0.5 | 1.0 |

-------------------

I4= GCNFL4 **ABUSE OF POWER/VIOLATIONS OF NORMS OF BEHAVIOR**

*(for example, blocking career development, discrimination, mobbing, sexual harassment)*

0) Never 0.5) Rarely 1.5) Occasionally 2) Frequently

| N | Mean | Standard Deviation | Median | Interquartile range |
| --- | --- | --- | --- | --- |
| 97 | 0.21 | 0.45 | 0.0 | 0 |

-------------------

I5=GCNFL5: **CAN WORK-RELATED GRIEVANCES BE TAKEN TO A RESPONSIBLE BODY FOR RESOLUTION**?

0) Yes, this can be done in an efficient and confidential manner.

1) In principle, yes, but this is not effective and/or cannot be done confidentially.

2) No, there is no possibility to redress grievances at work.

| N | Mean | Standard Deviation | Median | Interquartile range |
| --- | --- | --- | --- | --- |
| 97 | 0.58 | 0.63 | 1.0 | 1.0 |

=============================

**J. WORKLOAD & ACTIVITIES**

==============================

J1: **Handles patients who are severely disturbed**

Frequently (2), Occasionally (1) Rarely/Never (0)

| N | Mean | Standard Deviation | Median | Interquartile range |
| --- | --- | --- | --- | --- |
| 97 | 1.05 | 0.62 | 1.0 | 0 |

---------------------------------------------------------

J2: **Handles patients who cannot give a history**

Frequently (2), Occasionally (1) Rarely/Never (0)

| N | Mean | Standard Deviation | Median | Interquartile range |
| --- | --- | --- | --- | --- |
| 97 | 0.97 | 0.70 | 1.0 | 1.0 |

J3: **Number of inpatients** **under the physician’s direct care at one time**

0) None 3) Eleven to twenty

1.5) One to five 4) Over twenty

2) Six to ten

| N | Mean | Standard Deviation | Median | Interquartile range |
| --- | --- | --- | --- | --- |
| 95 | 1.53 | 1.36 | 1.5 | 3 |

J4: **Number of patients in Intensive care unit/coronary care unit**:

0) None 3) Six to ten

1.5) One to two 4) Over ten

2) Three to five

| N | Mean | Standard Deviation | Median | Interquartile range |
| --- | --- | --- | --- | --- |
| 95 | 0.70 | 0.85 | 0 | 1.5 |

J5: **Number of outpatients per shift**

0) None 3) Twenty-one to thirty

1.5) One to ten 4) Thirty-one to forty

2) Eleven to twenty 5) Over forty

| N | Mean | Standard Deviation | Median | Interquartile range |
| --- | --- | --- | --- | --- |
| 97 | 3.37 | 1.62 | 4.0 | 3.0 |

---------------------------------------------------------

J8: **Simultaneous attention:**  **Does it happen during your workday that several people seek your attention at the same time?** *(Including on the telephone)*

2) Yes, many times each day.

1.5) Yes, a few times each day.

1) Yes, but only once or twice each day.

0) No, rarely or never.

| N | Mean | Standard Deviation | Median | Interquartile range |
| --- | --- | --- | --- | --- |
| 97 | 1.49 | 0.55 | 1.5 | 1.0 |

J9. **If people simultaneously seek your attention, how many do so**?

0) Rarely more than two 1) Usually two, but sometimes more 2) Usually three or more

| N | Mean | Standard Deviation | Median | Interquartile range |
| --- | --- | --- | --- | --- |
| 97 | 0.80 | 0.69 | 1.0 | 1.0 |

------------------------------------------------------------------------------------------------------------

J10=OU3: **DURING WORK HOURS, MUST BE PHYSICALLY AT WORK, BUT THERE IS NOTHING TO DO**

**Includes time when waiting and cannot proceed with other work**

0) Never 0.5) Rarely 1) Occasionally 2) Frequently

| N | Mean | Standard Deviation | Median | Interquartile range |
| --- | --- | --- | --- | --- |
| 97 | 0.44 | 0.44 | 0.5 | 0.5 |

**Supervising**

J11a: **Supervises physicians at same or higher level**

|  | Count | % Valid |
| --- | --- | --- |
| Yes | 18 | 18.6 |

------------------------------------------------------------------------------------------------------------

J11b: **Supervises physicians with less training**

|  | Count | % Valid |
| --- | --- | --- |
| Yes | 51 | 52.6 |

------------------------------------------------------------------------------------------------------------

J11c: **Supervises medical students**

|  | Count | % Valid |
| --- | --- | --- |
| Yes | 56 | 57.7 |

------------------------------------------------------------------------------------------------------------

J11d: **Supervises other health professionals**

|  | Count | % Valid |
| --- | --- | --- |
| Yes | 48 | 49.5 |

J12: **Number supervised**

| N | Mean | Standard Deviation | Median | Interquartile range |
| --- | --- | --- | --- | --- |
| 97 | 4.24 | 8.56 | 2.0 | 4.0 |

CH3 (supervising): **DECISIONS AFFECT THE WORK OF OTHERS**

1 J11-J12=no direct supervision (some always implicit for physicians)

1.5 J11-J12=supervises one to two others

2 J11-J12 =supervises three or more others

| N | Mean | Standard Deviation | Median | Interquartile range |
| --- | --- | --- | --- | --- |
| 97 | 1.63 | 0.37 | 1.5 | 0.5 |

------------------------------------------------------------------------------------------------------------

J13: **Other duties besides clinical work**

|  | Count | % Valid |
| --- | --- | --- |
| Yes | 89 | 91.8 |

*Among those with other duties besides clinical work:*

J13a:**Teaching in small groups**

|  | Count | % Valid |
| --- | --- | --- |
| Yes | 65 | 73.0 |

J13b: **Lecturing to larger groups**

|  | Count | % Valid |
| --- | --- | --- |
| Yes | 19 | 21.3 |

J13c: **Research**

|  | Count | % Valid |
| --- | --- | --- |
| Yes | 62 | 69.7 |

J13d:**Administrative duties**

|  | Count | % Valid |
| --- | --- | --- |
| Yes | 36 | 40.4 |

-------------------------------

J14: **Separate time for non-clinical duties**

|  | Count | % Valid |
| --- | --- | --- |
| No | 68 | 76.4 |
| Yes | 21 | 23.6 |

*If no separate time, when are these other duties performed?*

J14a:: **Interspersed with clinical work**

|  | Count | % Valid |
| --- | --- | --- |
| Yes | 53 | 77.9 |

----------------------------------

J14b:: **Outside work hours**

|  | Count | % Valid |
| --- | --- | --- |
| Yes | 36 | 52.9 |

================

J15: **Publishing pressure Are you under pressure to publish/present new findings or results at Congresses/meetings outside your Institution?**

0.5) Yes, if I fail to do so in sufficient quantity, my career will suffer and I may even lose my position

0.25) Yes, but there are no major adverse consequences if I fail to do so.

0) No, such activity is entirely up to my own initiative and choice.

| N | Mean | Standard Deviation | Median | Interquartile range |
| --- | --- | --- | --- | --- |
| 97 | 0.17 | 0.20 | 0 | 0.25 |

--------------------------------

**Task-specific queries**:

J16: **Performs non-invasive diagnostic procedures**

|  | Count | % Valid |
| --- | --- | --- |
| Yes | 88 | 90.7 |

-------------------------------

J17: **Performs invasive procedures**

|  | Count | % Valid |
| --- | --- | --- |
| Yes | 59 | 60.8 |

-------------------------------

J18: **Performs surgical interventions**

|  | Count | % Valid |
| --- | --- | --- |
| Yes | 40 | 41.2 |

-------------------------------

J19: **Performs tasks outside the realm of a physician/work of other personnel**

|  | Count | % Valid |
| --- | --- | --- |
| Yes | 28 | 28.9 |

-------------------------------

J20=GCNFL7: **PERFORMS TASKS THAT SEEM POINTLESS**

|  | Count | % Valid |
| --- | --- | --- |
| Yes | 20 | 20.6 |

============================================

**K. RECENT CHANGES AT WORK**

============================================

K1: **An increase in workday length**

|  | Count | % Valid |
| --- | --- | --- |
| Yes | 58 | 59.8 |

K2: **An increase in time pressure/deadlines**

|  | Count | % Valid |
| --- | --- | --- |
| Yes | 42 | 43.3 |

K3: **An increase in responsibility**

|  | Count | % Valid |
| --- | --- | --- |
| Yes | 60 | 61.9 |

K6 **Recent promotion**

|  | Count | % Valid |
| --- | --- | --- |
| Yes | 12 | 12.4 |

***Acronyms and Abbreviations***

| AVOI | Symbolic aversiveness/avoidance/disaster potential (OSI aspect) | NOX | Noxious physical exposures (OSI aspect) |
| --- | --- | --- | --- |
| C | Central decision making (OSI level) | O | Output (OSI level) |
| CNFL | Conflict/uncertainty (OSI aspect) | OSI | Occupational Stressor Index |
| EP | Extrinsic time pressure (OSI aspect) | S | Strictness (OSI aspect) |
| G | General (OSI level) | T | Total |
| H | High demand (OSI aspect) | U | Underload (OSI aspect) |
| I | Input (OSI level) |  |  |

1. Cited reference [(1)] herein: Belkić, K. & Savić, C. *Job stressors and Mental Health: A Proactive Clinical Perspective*. (World Scientific, 2013). [↑](#endnote-ref-1)
